# Parkinson’s disease mutant Miro1 causes mitochondrial dysfunction and dopaminergic neuron loss

**DOI:** 10.1093/brain/awaf051

**Published:** 2025-02-06

**Authors:** Axel Chemla, Giuseppe Arena, Ginevra Sacripanti, Kyriaki Barmpa, Alise Zagare, Pierre Garcia, Vyron Gorgogietas, Paul Antony, Jochen Ohnmacht, Alexandre Baron, Jaqueline Jung, Frida Lind-Holm Mogensen, Alessandro Michelucci, Anne-Marie Marzesco, Manuel Buttini, Thorsten Schmidt, Anne Grünewald, Jens C Schwamborn, Rejko Krüger, Cláudia Saraiva

**Affiliations:** Luxembourg Centre for Systems Biomedicine (LCSB), University of Luxembourg, L-4362 Esch-sur-Alzette, Luxembourg; Luxembourg Centre for Systems Biomedicine (LCSB), University of Luxembourg, L-4362 Esch-sur-Alzette, Luxembourg; Luxembourg Centre for Systems Biomedicine (LCSB), University of Luxembourg, L-4362 Esch-sur-Alzette, Luxembourg; Luxembourg Centre for Systems Biomedicine (LCSB), University of Luxembourg, L-4362 Esch-sur-Alzette, Luxembourg; Luxembourg Centre for Systems Biomedicine (LCSB), University of Luxembourg, L-4362 Esch-sur-Alzette, Luxembourg; Luxembourg Centre for Systems Biomedicine (LCSB), University of Luxembourg, L-4362 Esch-sur-Alzette, Luxembourg; Luxembourg Center of Neuropathology (LCNP), Laboratoire National de Santé, L-3555, Dudelange, Luxembourg; Luxembourg Centre for Systems Biomedicine (LCSB), University of Luxembourg, L-4362 Esch-sur-Alzette, Luxembourg; Luxembourg Centre for Systems Biomedicine (LCSB), University of Luxembourg, L-4362 Esch-sur-Alzette, Luxembourg; Transversal Translational Medicine, Luxembourg Institute of Health (LIH), L-1445 Strassen, Luxembourg; Luxembourg Centre for Systems Biomedicine (LCSB), University of Luxembourg, L-4362 Esch-sur-Alzette, Luxembourg; Institute of Medical Genetics and Applied Genomics, University of Tübingen, 72076 Tübingen, Germany; Faculty of Science, Technology and Medicine, University of Luxembourg, L-4365 Esch-sur-Alzette, Luxembourg; Department of Cancer Research, Luxembourg Institute of Health (LIH), L-1210 Luxembourg, Luxembourg; Department of Cancer Research, Luxembourg Institute of Health (LIH), L-1210 Luxembourg, Luxembourg; Luxembourg Centre for Systems Biomedicine (LCSB), University of Luxembourg, L-4362 Esch-sur-Alzette, Luxembourg; Luxembourg Centre for Systems Biomedicine (LCSB), University of Luxembourg, L-4362 Esch-sur-Alzette, Luxembourg; Luxembourg Center of Neuropathology (LCNP), Laboratoire National de Santé, L-3555, Dudelange, Luxembourg; Institute of Medical Genetics and Applied Genomics, University of Tübingen, 72076 Tübingen, Germany; Luxembourg Centre for Systems Biomedicine (LCSB), University of Luxembourg, L-4362 Esch-sur-Alzette, Luxembourg; Institute of Neurogenetics, University of Lübeck, 160 Lübeck, Germany; Luxembourg Centre for Systems Biomedicine (LCSB), University of Luxembourg, L-4362 Esch-sur-Alzette, Luxembourg; Luxembourg Centre for Systems Biomedicine (LCSB), University of Luxembourg, L-4362 Esch-sur-Alzette, Luxembourg; Transversal Translational Medicine, Luxembourg Institute of Health (LIH), L-1445 Strassen, Luxembourg; Parkinson Research Clinic, Centre Hospitalier de Luxembourg, L-1210, Luxembourg, Luxembourg; Luxembourg Centre for Systems Biomedicine (LCSB), University of Luxembourg, L-4362 Esch-sur-Alzette, Luxembourg

**Keywords:** p.R272Q Miro1, neurodegeneration, calcium homeostasis, α-synuclein, patient-specific iPSC-derived models, knock-in mice

## Abstract

The complex and heterogeneous nature of Parkinson's disease (PD) is still not fully understood. However, increasing evidence supports mitochondrial impairment as a major driver of neurodegeneration. Miro1, a mitochondrial GTPase encoded by the *RHOT1* gene, is involved in mitochondrial transport, mitophagy and mitochondrial calcium buffering, and is therefore essential for maintaining mitochondrial homeostasis. Recently, Miro1 has been linked genetically and pathophysiologically to PD, further supported by the identification of heterozygous variants of Miro1 in patients.

Herein, we used patient-derived cellular models alongside knock-in mice to investigate Miro1-dependent pathophysiological processes and molecular mechanisms underlying neurodegeneration in PD.

Experimental work performed in induced pluripotent stem cell (iPSC)-derived models, including midbrain organoids and dopaminergic neuronal cell cultures from a PD patient carrying the p.R272Q Miro1 mutation as well as healthy and isogenic controls, indicated that the p.R272Q Miro1 mutation leads to increased oxidative stress, disrupted mitochondrial bioenergetics and altered cellular metabolism. These changes were accompanied by increased α-synuclein levels and a significant reduction of dopaminergic neurons. Moreover, the p.R272Q Miro1 mutation—located in the calcium-binding domain of the GTPase—disrupted calcium homeostasis, resulting in calcium-dependent activation of calpain proteases and the subsequent cleavage of α-synuclein. Knock-in mice expressing p.R285Q Miro1 (the murine orthologue of the human p.R272Q mutation) displayed accumulation of phosphorylated α-synuclein in the striatum and a significant loss of dopaminergic neurons in the substantia nigra pars compacta, accompanied by behavioural alterations.

These findings demonstrate that mutant Miro1 is sufficient to comprehensively model PD-relevant phenotypes *in vitro* and *in vivo*, reinforcing its pivotal role in PD pathogenesis.

## Teaser

Mutations in the gene encoding Miro1, a mitochondrial GTPase, have been identified in patients with Parkinson’s disease. Using neuronal cell cultures and midbrain organoids, Chemla *et al.* show that a mutation in Miro1 causes mitochondrial dysfunction and dopaminergic neuron loss, further supporting its role in Parkinson’s disease pathology.

## Introduction

Parkinson's disease (PD) is the fastest-growing neurodegenerative disorder worldwide.^1^ PD patients show a massive loss of dopaminergic neurons in the substantia nigra pars compacta (SNpc) and the presence of α-synuclein-rich aggregates (i.e. Lewy bodies) in the surviving cells, culminating in the well-described motor symptoms.^2^ Despite its heterogeneity, alterations in essential cellular processes, such as mitochondrial dysfunction, oxidative stress, calcium dysregulation, impaired autophagy and mitophagy, protein misfolding and apoptosis, are commonly observed in PD.^2^

Miro1 (mitochondrial Rho GTPase protein) is a conserved element of the mitochondrial motor/adaptor complex. It is composed of a C-terminal transmembrane domain, which binds to the outer mitochondrial membrane, and two EF-hand calcium-binding domains flanked by two GTPase domains.^3^ Miro1 plays a fundamental role in regulating mitochondrial dynamics, calcium homeostasis and mitophagy.^4^ A pathological stabilization of physiological Miro1, in which its degradation induced by mitochondrial depolarization is impaired, was observed in fibroblasts^5^ and induced pluripotent stem cell (iPSC)-derived neurons^6^ from sporadic and monogenic PD patients. Moreover, Miro1 was found to physically or functionally interact with proteins encoded by well-established PD-causative genes, such as PINK1, Parkin, LRRK2 and α-synuclein, suggesting a possible converging role of Miro1 in controlling different cellular activities and pathways relevant for neurodegeneration in PD.^5,7-10^ Previously, we identified four PD patients carrying distinct heterozygous mutations in the *RHOT1* gene encoding Miro1,^11,12^ all presenting mitochondria-related alterations.^11-13^

Herein, we dissected the importance and potential pathways of Miro1 in PD pathogenesis by focusing on patient-specific p.R272Q Miro1 mutant models and the *in vivo* p.R285Q orthologue Miro1 mutation, which is located in the N-terminal EF-hand domain of the protein and is responsible for calcium sensing.^4^

Using iPSC-derived dopaminergic neurons and midbrain organoids from a p.R272Q Miro1 patient and both isogenic and sex/age-matched controls, we demonstrated that the Miro1 mutation altered mitochondrial bioenergetics, impaired calcium homeostasis, promoted α-synuclein accumulation and cleavage via calpain activation and caused loss of dopaminergic neurons. *In vivo*, degeneration of dopaminergic neurons and accumulation of phosphorylated α-synuclein, accompanied by behavioural deficits was observed in aged p.R285Q Miro1 knock-in mice. Our findings indicate that the p.R272Q Miro1 mutation is sufficient to recapitulate relevant PD phenotypes *in vitro* and *in vivo*, reinforcing the role of Miro1 in the pathogenesis of PD.

## Materials and methods

### Midbrain organoids

#### Generation

Neuroepithelial stem cells (NESC), derived from human iPSC,^14^ were used to generate midbrain organoids^15,16^ from five different cell lines (Table 1 and Supplementary material, ‘Methods’ section). Unless stated otherwise, *n* consists of the number of cell lines, independent organoid derivation (batches) and independently cultured organoids within each batch.

**Table 1:** Description of cell lines used in *in vitro* experiments

ID	Diagnosis	Genotype	Sex	Age of sampling	Age of onset	Reference
Ctrl1	Healthy	wt/wt	Female	72	–	Berenguer-Escuder *et al*.^11^
Ctrl2	Healthy	wt/wt	Female	68	–	Zagare *et al*.^17^
Ctrl3	Healthy	wt/wt	Female	63	–	Zagare *et al*.^17^
PD-R272Q	PD	Miro1 p.R272Q/wt	Female	78	70	Chemla *et al*.^18^
iCtrl	PD background/Healthy genotype	wt/wt	Female	78	70	Chemla *et al*.^18^

Ctrl = control; iCtrl = isogenic control; wt = wild-type.

#### Single-cell RNA sequencing

Thirty-day-old embedded midbrain organoids from Ctrl2, PD-R272Q and iCtrl were used (GEO: GSE237133). Libraries were constructed using 6000 cells per condition and sequenced on the Illumina NovaSeq 6000 using a 2 × 150-base pair approach (90 GB depth) (Supplementary material, ‘Methods’ section).

#### Western blotting

Three embedded organoids per cell line per batch were pooled, snap-frozen and protein extracted using 100 µl RIPA buffer (Abcam, Cat. No. ab156034) supplemented with protease and phosphatase inhibitors (Roche, Cat. No. 11697498001; Merck, Cat. No. 524629) as previously described^19^ with a slight modification. Herein, membranes were revealed using a STELLA 8300 imaging system (Raytest) after incubation with a chemiluminescent substrate (Life Technologies, Cat. No. 34580). Primary and secondary antibodies used are listed in Table 2. Proteins of interest were quantified using ImageJ software (Wayne Rasband; RRID SCR_003070).

**Table 2:** List of antibodies used for Western blotting and immunostaining experiment in midbrain organoids, dopaminergic neurons and mice

Antibody	Species	Company	Cat. No.	RRID	WB	IF	Model
**Primary antibodies**							
α-Syn (2A7; aa 61–95)	rabbit	Novus Biologicals	NBP1-05194	AB_1555287	–	1:1000	MO
α-Syn (C42; aa 15–23)	mouse	BD Transduction	610 787	AB_398107	1:1000	–	DaN Mouse
α-Syn P-S129 (11A5)	mouse	Prothena	1347	Non-commercial	1:1000	–	Mouse
α-Syn P-S129	rabbit	Abcam	ab51253	AB_869973	–	1:1000	Mouse
α-Syn (33) oligomer specific	rabbit	Merck	ABN 2265	AB_2910172	1:750	–	DaN
β-Actin	mouse	Cell Signaling	3700S	AB_2242334	1:50 000 1:20 000 1:20 000	–	MO DaN Mouse
CDK5 (EP715Y)	rabbit	Abcam	ab40773	AB_726779	1:1000	–	DaN
DAT	rat	Millipore	ab369	AB_2190413	–	1:1000	Mouse
FTL	rabbit	Proteintech	10727-1-AP	AB_2278673	1:500	–	MO
MAP2	chicken	Abcam	ab92434	AB_2138147	–	1:1500	MO
MnSOD	rabbit	Abcam	ab13533	AB_300434	1:750	–	Mouse
p62	mouse	BD Transduction	610 833	AB_398152	1:1000	–	Mouse
OXPHOS (total)	mouse	Abcam	ab110413	AB_2629281	1:100	–	Mouse
TH	rabbit	Santa Cruz	sc-14007	AB_671397	1:1000	–	MO
TH	rabbit	Abcam	ab112	AB_297840	–	1:1000	MO
TH	rabbit	Millipore	ab152	AB_390204	–	1:1000	Mouse
TH	chicken	Abcam	ab76442	AB_1524535	–	1:1000 or 1:750	Mouse
TOM20	rabbit	Cell Signaling	42 406	AB_2687663	1:1000	–	MO Mouse
TOM20	Rabbit	Santa Cruz	SC11415	AB_2207533	–	1:500	Mouse
TUJ1	mouse	BioLegend	801 201	AB_2313773	1:50 000	1:1000	MO
VDAC	rabbit	Cell Signaling	4661	AB_10557420	1:1000	–	MO
Vinculin (E1E9V)	rabbit	Cell Signaling	13 901	AB_2728768	1:1000	–	DaN Mouse
**Secondary antibodies**							
Anti-chicken IgG (H + L) AF-488	goat	Molecular probes	A-11039	AB_142924	–	1:1000	Mouse
Anti-chicken IgY (H + L) AF-647	goat	Thermo Fisher Scientific	A-21449	AB_10374876	–	1:1000	MO
Anti-mouse IgM AF-568	goat	Thermo Fisher Scientific	A-21043	AB_2535712	–	1:1000	MO
Anti-rabbit IgG (H + L) AF-488	goat	Thermo Fisher Scientific	A-11034	AB_2576217	–	1:1000	MO Mouse
Anti-rabbit IgG AF-647	goat	Thermo Fisher Scientific	A-27040	AB_2536101	–	1:1000	Mouse
Anti-rat IgG (H + L) AF-647	goat	Molecular Probes	A21247	AB_141778	–	1:1000	Mouse
HRP anti-rabbit	donkey	Cytiva	NA934	AB_772206	1:1000	–	MO
HRP anti-rabbit	goat	Thermo Fisher Scientific	A24537	AB_2536005	1:5000	–	DaN Mouse
HRP anti-mouse	sheep	Cytiva	NA931	AB_772210	1:1000	–	MO
HRP anti-mouse	goat	Thermo Fisher Scientific	A24524	AB_2535993	1:5000	–	DaN Mouse

α-Syn = α-synuclein; aa = amino acid; CDK5 = cyclin-dependent kinase 5; DaN = dopaminergic neurons; DAT = dopamine transporter; FTL = ferritin light chain; IF = immunofluorescence; HRP = horseradish peroxidase; MAP2 = microtubule-associated protein 2; MnSOD = manganese superoxide dismutase; MO = midbrain organoids; OXPHOS = oxidative phosphorylation; RRID = Research Resource Identifier; TH = tyrosine hydroxylase; TUJ1 = class III beta-tubulin; VDAC = voltage-dependent anion channel; WB = western blotting.

#### Immunofluorescence staining

Organoid staining was done using a modified published protocol^20^ in 70 µm sections. Blocking consisted of 5% goat serum (Thermo Fisher Scientific, Cat. No. 10000C) and 0.5% Triton X-100 (Carl Roth, Cat. No. 3051.3) in phosphate buffered saline (PBS). Table 2 summarizes the antibodies used. Terminal deoxynucleotidyl transferase dutp nick-end labelling (TUNEL) assay was performed using the *In Situ* Cell Death Detection Kit, TMR red (Merck, Cat. No. 12156792910) in combination with TH staining. For this, secondary antibodies (Table 2) and Hoechst-33342 (Invitrogen, Cat. No. 62249; 1:10 000) were diluted in the TUNEL kit components and incubated for 1 h at room temperature. Image acquisition and analysis were done following a published pipeline^21,22^ on a 20× objective from the Yokogawa CV8000 high content screening confocal microscope. At least one section from two organoids per cell line from three batches were analysed.

#### Mito stress test

A Seahorse XF Cell Mito Stress Test (Agilent) was performed in 35-day-old non-embedded organoids using Seahorse XFe96 Spheroid FluxPak (Agilent, Cat. No. 102905-100) according to the manufacturer's instructions (Supplementary material, ‘Methods’ section).

#### Flow cytometry

Non-embedded 30-day-old organoids were dissociated to assess reactive oxygen species (ROS) and mitochondrial membrane potential (MMP) by analysing 10 000 events on the flow cytometer BD LSRFortessa (BD Biosciences) and FlowJo software (v.10.8.1; RRID SCR_008520) (Supplementary material, ‘Methods’ section).

#### Metabolomics

Polar intracellular metabolites were analysed in embedded organoids using hydrophilic interaction liquid chromatography and mass spectrometry (Supplementary material, ‘Methods’ section).

#### Lactate dehydrogenase assay

A lactate dehydrogenase (LDH)-Glo Cytotoxicity Assay (Promega, Cat. No. J2380) was used in 30-day-old non-embedded organoids according to the manufacturer's instructions. Relative levels of LDH were calculated by normalizing LDH luminescence to the organoid area.

### Dopaminergic neurons

#### Generation of dopaminergic neurons

iPSC-derived NESC^14^ (Ctrl1, PD-R272Q, and iCtrl; Table 1) were seeded onto Geltrex-coated 6-well plates at a density of 3 × 10^6^ cells/well. Cells were kept in N2B27 media supplemented with 1 µM purmorphamine (Sigma-Aldrich, Cat. No. SML0868-25 mg), 200 µM ascorbic acid and 100 ng/ml fibroblast growth factor 8b (FGF8b, Peprotech 10–25) for 8 days. Between Days 8 and 10, FGF8b was removed and purmorphamine reduced to 0.5 µM. Finally, from Day 10 onwards, differentiating neurons were culture in maturation media. For statistical purposes, the number of independent derivations (*n*) was considered.

#### Bulk RNA sequencing

Thirty-day-old dopaminergic neurons were used for RNA extraction and subsequent sequencing (GEO: GSE238129; Supplementary material, ‘Methods’ section).

#### Imaging

Live imaging of dopaminergic neurons was performed to measure intracellular ROS, MMP, and calcium influx (Supplementary material, ‘Methods’ section) using a Yokogawa CV8000 microscope, with a 60× or 20× objective, under controlled CO_2_ and temperature.

#### Seahorse

OCR were measured in whole cells following the Agilent Seahorse Mito Stress Test instructions (Supplementary material, ‘Methods’ section).

#### Western blotting

Western blotting was performed as previously described, after cell lysis in either sodium dodecyl sulfate (SDS)^23^ or Radio-Immunoprecipitation Assay (RIPA)^24^ buffer. Proteins were quantified using the Pierce BCA assay kit, according to the manufacturer's instructions, and then resolved by SDS-PAGE. Protein bands were detected with the respective antibodies (Table 2) using enhanced chemiluminescence (ECL) detection reagent (Sigma-Aldrich, Cat. No. GERPN2232 or Amersham, Cat. No. RPN2235) on the STELLA imaging system or Odyssey XF Imager (Li-Cor), respectively. Densitometric analysis was performed using the ImageJ software.

#### Calpain activity

Neurons were incubated with 100 μM of calpain substrates *N*-succinyl-Leu-Tyr-7-amido-4-methylcoumarin (Suc-LY-AMC, Enzo, Cat. No. ALX-260-054) or *N*-succinyl-Leu-Leu-Val-Tyr-7-amido-4-methylcoumarin (Suc-LLVY-AMC, Enzo, Cat. No. BML-P802-0005) in assay buffer (20 mM Tris-HCl pH 7.5, 5 mM MgCl_2,_ 0.1 mM EDTA, 1 mM DTT, 2.5 mM CaCl_2_) after being washed once. Fluorescence was measured at 37°C in a Cytation5M plate reader (BioTek, excitation 380 nm, emission 460 nm). Cells pre-treated for 4 h with 20 μμ of the calpain inhibitor MDL-28170 (Santa Cruz, Cat. No. 88191-84-8) were used as control. Calpain activity was expressed as fluorescent intensity per μg of total protein.

### Mice

Generation of heterozygous (wt/R285Q) and homozygous (R285Q/R285Q) knock-in p.R285Q Miro1 mutant mice (B6.Miro1^tmR285QHmgu^) was done using CRISPR/Cas9-mediated gene editing in C57BL/6N mouse zygotes (Supplementary material, ‘Methods’ section).

#### Rotarod behaviour test

The analysis was performed in a red light illumination room during animals’ nighttime. Mice were placed on the rod, which accelerated from 4 to 40 rpm over a duration of 300 s. If a mouse fell within the first 10 s of a trial, the measurement was repeated. Per test day, three trials were carried out with a minimum of 15 min rest in between. The mean latency to fall per mouse was calculated as the mean value of the three trials. The Rotarod was performed twice with the same female mice at ages 20 and 21 months, with no training prior to the first trial. One female was excluded based on its significantly higher weight.

#### Brain processing

Mice were deeply anaesthetized with 150 mg/kg ketamine + 1 mg/kg medetomidine and transcardiacally perfused with PBS. Brains were longitudinally divided into the two hemispheres. One hemisphere was dissected into striatum and midbrain and stored at −80°C, while the other was fixed in 4% PFA for 48 h at 4°C and then stored in 0.2% sodium azide PBS. Young (3 to 6-month-old) and aged (15-month-old) mice were analysed. Immunocytochemistry using serial 50 µm thick parasagittal free-floating sections was done (Supplementary material, ‘Methods’ section).^25^ Brain tissues were homogenized using a Retsch TissueLyser MM200 for 30 s at 25 Hz in the presence of one iron bead, and protein lysates were processed for western blotting as described earlier in the ‘Western blotting’ section (Table 2).

### Data analysis and statistics

Data visualization and statistical analyses were performed using GraphPad Prism (version 10.1.2) and RStudio (version R 4.3.0) software. Data were expressed as mean ± standard error of the mean (SEM) or median with maximum/minimum. For midbrain organoids, LDH and TUNEL, outlier removal was performed with the Rosner Test (EnvStats package, RStudio). Two-way ANOVA was used for experiments involving two categorical variables (i.e. cell status and treatment). After the Shapiro test, normally distributed data were analysed using ANOVA with a *post hoc* Tukey’s Honest Significant Difference test or unpaired *t*-test, while the non-parametric Kruskal–Wallis or Mann–Whitney tests were used for non-normal data. *P* < 0.05 was considered to represent statistical significance.

### Ethical approval

Ethical approvals have been obtained from the Luxembourg National Research Ethics Committee for human-derived lines (DiMo-PD CNER #201411/05 and ERP 18-082 ivPD) and animal studies (Règlement grand-ducal du 11 janvier 2013 in line with the European Directive 2010/63/EU.w).

## Results

### p.R272Q Miro1 mutant midbrain organoids revealed transcriptomic deregulation of PD-related pathways

**Figure 1:**
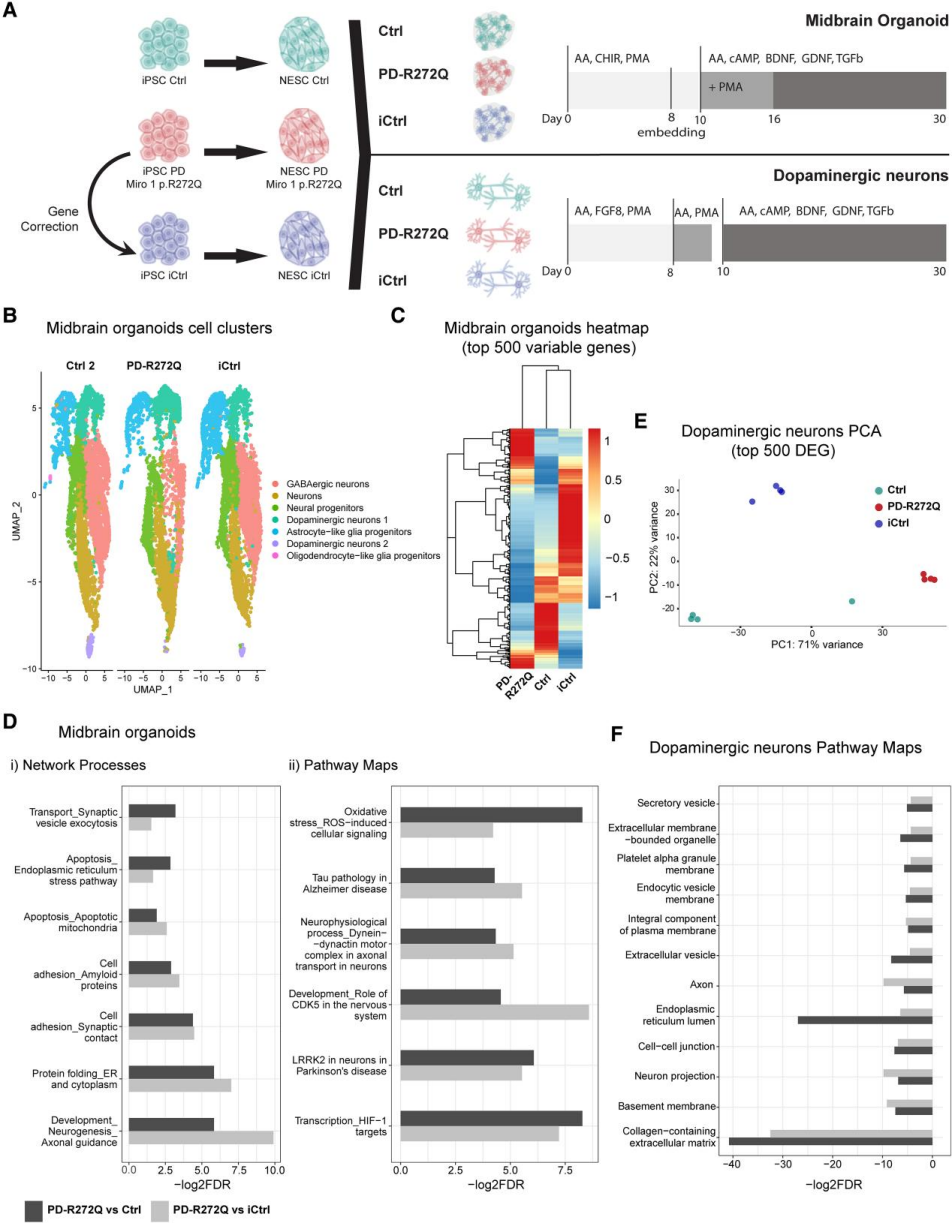
**Parkinson's disease-related pathways were deregulated in p.R272Q Miro1 mutant midbrain organoids and dopaminergic neurons.** (**A**) Schematic representation of the *in vitro* models and culture conditions used. (**B**) UMAP visualization of healthy control (Ctrl 2), p.R272Q Miro1 mutant (PD-R272Q) and isogenic control (iCtrl) organoids single-cell RNA sequencing (scRNAseq) data showed seven unique cell clusters. Dots are colour-coded by cell cluster and represent individual cells. (**C**) Heat map displaying the top 500 differentially expressed genes (DEG) showed a separation between PD-R272Q organoids and controls. (**D**) Graphics depict the most PD-relevant deregulated network processes (**i**) and pathways (**ii**) between PD-R272Q and Ctrl or iCtrl organoids from the top 25 most deregulated ones (Supplementary Fig. 2). (**E**) Principal component analysis (PCA) plot shows separation at PC1 in PD-R272Q dopaminergic neurons compared with Ctrl and iCtrl, based on the top 500 DEG identified from bulk RNAseq analysis. (**F**) Graphic displaying the deregulated gene ontology terms of PD-R272Q dopaminergic neurons versus healthy or isogenic controls. (**B**–**F**) Significance was considered when *P*-adjusted value <0.05. FDR = false discovery rate; PD = Parkinson’s disease; UMAP = Uniform Manifold Approximation and Projection.

Miro1 relevance in PD pathology was assessed using two different patient-specific iPSC-derived models: midbrain organoids and dopaminergic neuronal cultures. We analysed iPSC obtained from (i) a PD patient carrying the *RHOT1* c.815G>A mutation (NM_001033568; p.R272Q Miro1, PD-R272Q); (ii) the corresponding gene-edited line where the point mutation was corrected by CRISPR/Cas9 (isogenic control, iCtrl)^18^; and (iii) control lines from age- and sex-matched healthy individuals (Ctrl) (Fig. 1A and Table 1).

Midbrain organoids are 3D complex structures with diverse cell types, including functional dopaminergic neurons, and defined spatial orientations mimicking the human midbrain,^15, 26^ which are capable of modelling PD phenotypes in a robust manner.^20^  ^, 27^ Midbrain organoids derived from one healthy individual (Ctrl2), PD-R272Q, and iCtrl were analysed using single-cell RNA sequencing (scRNAseq). The Seurat integration workflow identified seven different cellular clusters (Fig. 1B) based on the combination of the La Manno *et al*.^28^ gene list and the expression of cell and maturity-specific markers (Supplementary Fig. 1A and B). UMAP representation showed the presence of midbrain-relevant cell types within the organoid model, including the presence of a neural progenitor cluster, and two dopaminergic neuron clusters: dopaminergic neurons 1 and dopaminergic neurons 2, the latter expressing higher levels of tyrosine hydroxylase (TH; Fig. 1B and Supplementary Fig. 1B). Hierarchical clustering of the top 500 variable genes showed that PD-R272Q organoids were clustered separately from healthy and isogenic controls, suggesting a significant influence of the p.R272Q Miro1 mutation on the transcriptome (Fig. 1C). Then, the computed differentially expressed genes (DEG) were used to perform pathway enrichment analysis between PD-R272Q and either Ctrl or iCtrl organoids. The most relevant shared dysregulated processes and pathways (Fig. 1D) from the top 25 (Supplementary Fig. 2A) showed significant deregulation of processes related to neurogenesis, synapse contacts and exocytosis, endoplasmic reticulum (ER) and mitochondrial apoptosis. Pathway maps also showed deregulation of ROS, transcription of HIF-1 targets (important for iron homeostasis and oxidative stress defense^29^), LRRK2 in PD neurons and dynein-dynactin motor complex in axonal transport in neurons. Deregulation of these pathways is commonly seen in PD.^2^ Remarkably, the main transcriptomic alterations induced by mutant Miro1 seemed to be driven by the dopaminergic neuron clusters (Supplementary Fig. 2B).

The transcriptomic profile of iPSC-derived dopaminergic neurons was also assessed using bulk RNAseq (Supplementary Fig. 3). Principal component analysis (PCA) showed a clear separation between PD-R272Q and controls (Fig. 1E). Gene ontology (GO) and enriched terms analysed in PD-R272Q versus Ctrl or iCtrl conditions showed deregulation in neuronal projections, axons, secretory vesicles, ER and lysosomal compartments (Fig. 1F). These results further support specific p.R272Q Miro1-dependent transcriptome alterations within dopaminergic neurons.

### p.R272Q Miro1 mutation caused mitochondrial dyshomeostasis

**Figure 2:**
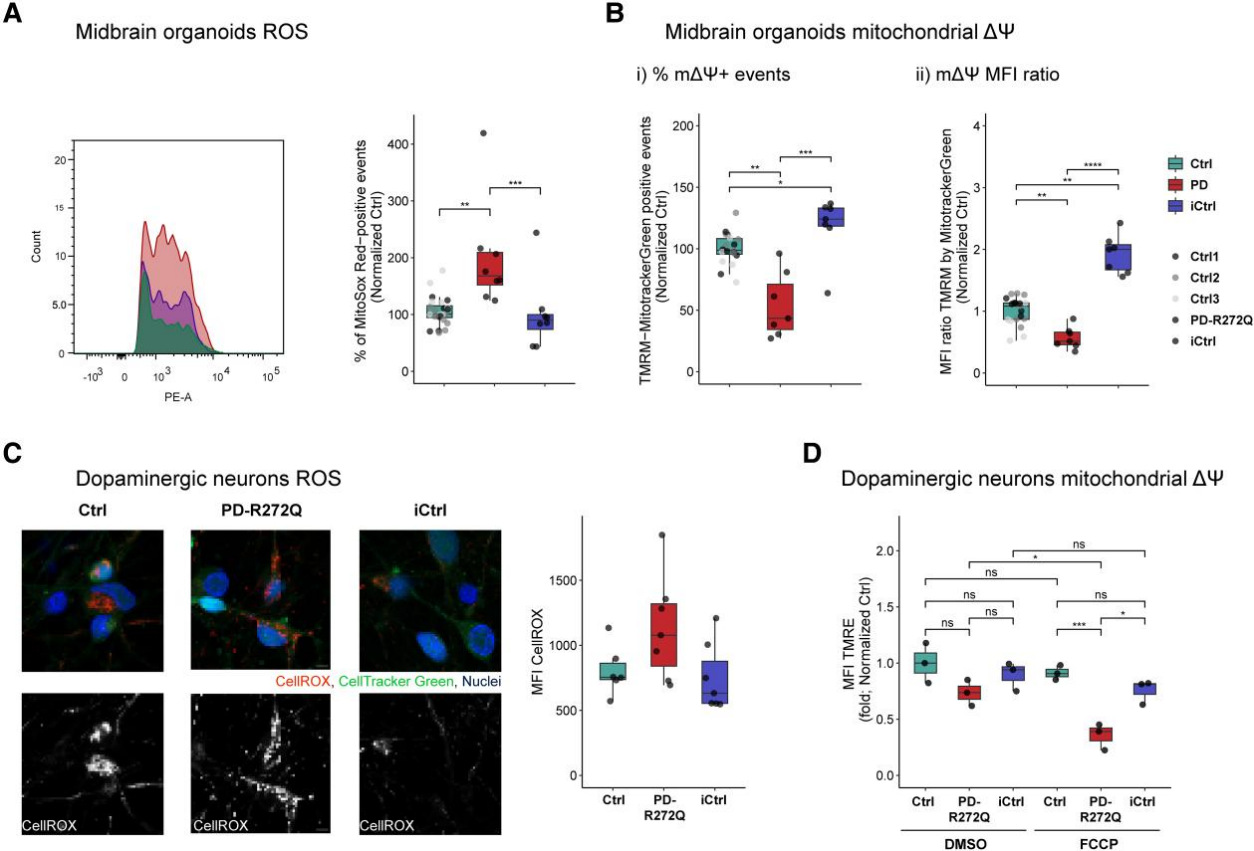
**p.R272Q Miro1 mutation increased ROS and impaired mitochondrial membrane potential *in vitro*.** (**A**) Flow cytometry representation (*left*) and quantification (*right*) of the percentage of MitoSox Red-positive events. *n* = 8–24 from six independent derivations. (**B**) Evaluation of mitochondrial membrane potential (MMP or mΔΨ) using the specific marker TMRM by flow cytometry in organoids. [**B**(**i**)] Percentage of double-positive events for TMRM and MitoTracker Green. [**B**(**ii**)] Mean fluorescent intensity (MFI) ratio between TMRM and MitoTracker Green within the double-positive events. *n* = 5–21 from five independent derivations. (**C**) Evaluation of cellular ROS in dopaminergic neurons using live imaging. *Left*: Representative images of dopaminergic neurons containing CellRox Deep Red (ROS marker; red or white), CellTracker Green (cellular marker; green) and Hoechst (blue, nuclei) in Ctrl, PD-R272Q and iCtrl. Scale bar: 20 µm. *Right*: Graphs depicting the intracellular CellRox MFI values normalized to iCtrl. *n* = 6–7 independent derivations. (**D**) MMP evaluation in dopaminergic neurons, in the absence (DMSO) or presence of FCCP, by live imaging quantification of mitochondrial TMRE MFI signal. *n* = 3–4 independent derivations. All data are presented as median with maximum/minimum or mean ± standard error of the mean. *P < 0.05, **P < 0.01, ***P < 0.001 using non-parametric multiple comparison Kruskal–Wallis test (**A** and **B**), one-way ANOVA with a *post hoc* Tukey’s Honest Significant Difference test (**C**) or two-way ANOVA (**D**). Ctrl = control; iCtrl = isogenic control; PD = Parkinson’s disease; ROS = reactive oxygen species.

Pathway enrichment analysis on midbrain organoids scRNAseq data revealed deregulation of ROS-related genes (Supplementary Fig. 4A), pointing to a possible impairment of mitochondrial homeostasis in PD-R272Q organoids. For example, *PRKCB*, encoding protein kinase C beta type, negatively correlates with mitochondrial energetic state and autophagy^30^; *UBL5 is* important for coping with mitochondrial stress^31^; and *FTL* is essential for iron metabolism and iron-induced stress.^29^ MitoSOX Red was used to measure superoxide levels via flow cytometry. Mutant Miro1 organoids showed a significant increase in the number of MitoSOX-positive events compared with healthy and isogenic controls (Fig. 2A), despite the similar mitochondrial content (Supplementary Figs 4B and C). Accordingly, Ferritin light chain (FTL) protein levels were decreased in mutant organoids (Supplementary Fig. 4D), although individual variability was observed. MMP was assessed by the percentage and the mean fluorescent intensity (MFI) of the TMRM-positive signal within the total mitochondria (MitoTracker Green signal). Mutant p.R272Q Miro1 organoids showed a significant reduction in the percentage of mitochondria with intact MMP and an overall lower MMP in comparison to controls (Fig. 2B), despite scRNAseq data showing an increased expression of mitochondrial genes (Supplementary Fig. 4E). The isogenic control showed a significantly higher MMP compared to healthy controls, which could reflect the slight increase in the expression of mitochondrial genes and/or inter-individual variability.

Mitochondria-related phenotypes were also assessed in dopaminergic neurons using image-based assays. Intracellular ROS were quantified by the MFI of the CellROX Deep Red-positive cells from total cells (CellTracker Green-positive signal). No significant changes were observed due to the high variability, but PD-R272Q neurons showed a tendency to have higher ROS compared to iCtrl (*P* = 0.07; Fig. 2C). For MMP measures, MFI of TMRE (a marker of intact MMP) within the total mitochondrial signal was quantified in basal conditions (DMSO, dimethyl sulfoxide) and under mitochondrial depolarization (FCCP). No significant differences were observed in basal conditions. However, in response to mitochondrial depolarization, PD-R272Q neurons showed a reduced ability to cope with stress and displayed a significantly reduced MMP compared with both controls (Fig. 2D). Altogether, our results showed that despite some influence of the patient's genetic background, the p.R272Q Miro1 mutation seems to increase susceptibility to mitochondrial damage, which might impact mitochondrial energy production.

### p.R272Q Miro1-induced mitochondrial stress prompted bioenergetic deficits

**Figure 3:**
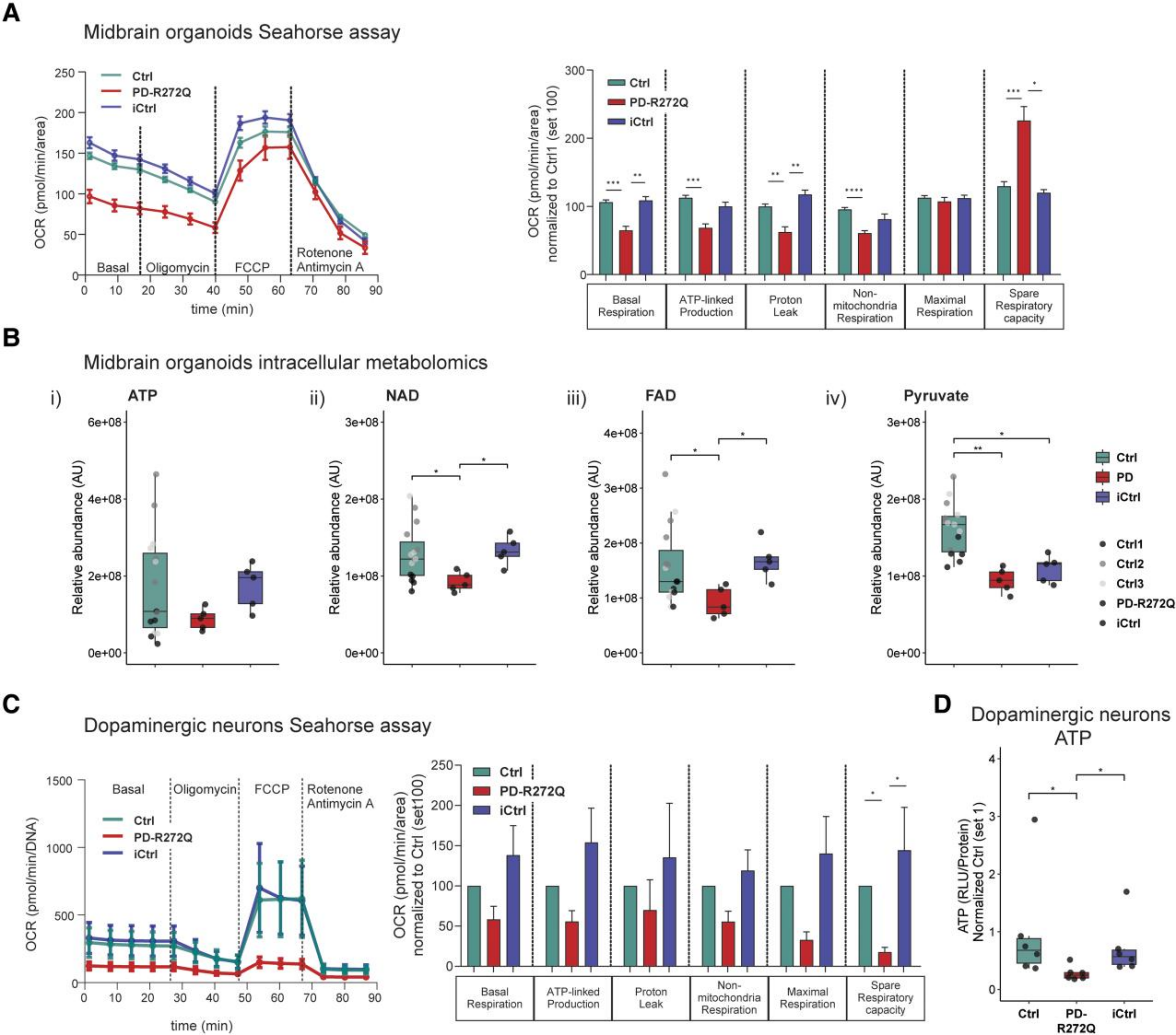
**p.R272Q Miro1 mutation caused mitochondrial bioenergetic deficits *in vitro*.** (**A**) Assessment of mitochondrial function in p.R272Q Miro1 mutant (PD-R272Q), healthy (Ctrl) and isogenic (iCtrl) midbrain organoids using Seahorse Mito Stress Test from Agilent. *Left*: Representative graphic depicts oxygen consumption rate (OCR) over time (minutes) under a specific set of drugs: oligomycin, FCCP, and antimycin A and rotenone. *Right*: Bar graph shows quantification of the different parameters calculated from the assay. Ctrl *n* = 27, PD *n* = 9, iCtrl *n* = 5 from 5–9 independent derivations. (**B**) Relative abundance of (**i**) ATP, (**ii**) NAD, (**iii**) FAD and (**iv**) pyruvate intracellular metabolites in midbrain organoids upon targeted liquid chromatography-mass spectrometry. *n* = 5–15 from two independent derivations. (**C**) OCR by time (*left*) and feature quantification (*right*) from Seahorse Mito Stress Test in dopaminergic neuronal cultures. *n* = 4 independent derivations. (**D**) Intracellular ATP levels in dopaminergic neurons. *n* = 6 independent derivations. All data are represented as mean ± standard error of the mean or median with maximum/minimum. *P < 0.05, **P < 0.01, ***P < 0.001, ****P < 0.0001 using non-parametric multiple comparison Kruskal–Wallis test. PD = Parkinson’s disease.

The influence of the p.R272Q Miro1 mutation on organoids’ mitochondrial respiration was analysed using Seahorse technology. As shown in Fig. 3A (left panel), the pattern of OCR curves was significantly different between PD-R272Q organoids and both controls. In particular, PD-R272Q organoids showed significantly lower basal respiration and proton leak. PD-R272Q organoids also displayed significantly lower ATP-linked production and non-mitochondrial respiration compared to Ctrl but not to iCtrl, suggesting a partial contribution of the patient's genetic background. However, when treated with the mitochondrial uncoupler FCCP, PD-R272Q organoids were still able to respond similarly to control organoids, shown by the comparable levels of maximal respiration and higher spare respiratory capacity observed (Fig. 3A, right panel). These results suggest a certain adaptability of mutant midbrain organoids to stress conditions, most probably driven by non-dopaminergic neuronal cell types (e.g. astrocytes). Likewise, organoids’ metabolomic analysis did not show a significant difference in ATP levels [Fig. 3B(i)], indicating potentially compensatory mechanisms from non-mitochondrial metabolic pathways. Nevertheless, PD-R272Q organoids showed significant lower relative abundance of the oxidized forms of co-factors NAD and FAD [Fig. 3B(ii and iii)] essential for mitochondrial function as well as pyruvate [Fig. 3B(iv)], a key metabolite in glucose metabolism that feeds the tricarboxylic acid cycle.

We further assessed the specific role of the p.R272Q mutation in iPSC-derived dopaminergic neurons, which showed a tendency towards a decrease of all OCR-related parameters compared to controls, albeit not significant (Fig. 3C). Interestingly, dopaminergic neurons displayed a significant reduction in spare respiratory capacity (Fig. 3C, right panel), indicative of their inability to produce energy via oxidative phosphorylation under high-energy demand. This was further supported by the significant decrease of intracellular ATP levels in comparison with both controls (Fig. 3D). Despite not reaching statistical significance, a consistent tendency towards a decrease in NAD(H) and NADP(H) co-factors abundance was observed in PD-R272Q neurons (Supplementary Fig. 5A). Furthermore, these bioenergetic alterations are supported by an increased uptake of pyruvate, glutamate, and glycine from the extracellular medium of PD-R272Q neurons (Supplementary Fig. 5B). Interestingly, these energy alterations seemed to be more pronounced between PD-R272Q versus iCtrl than PD-R272Q versus Ctrl, indicating a p.R272Q Miro1 mutation-dependent effect.

Overall, these results support mitochondria-related bioenergetic defects caused by the p.R272Q Miro1 mutation.

### Mutant dopaminergic neurons showed α-synuclein accumulation via calcium-dependent calpain activation

**Figure 4:**
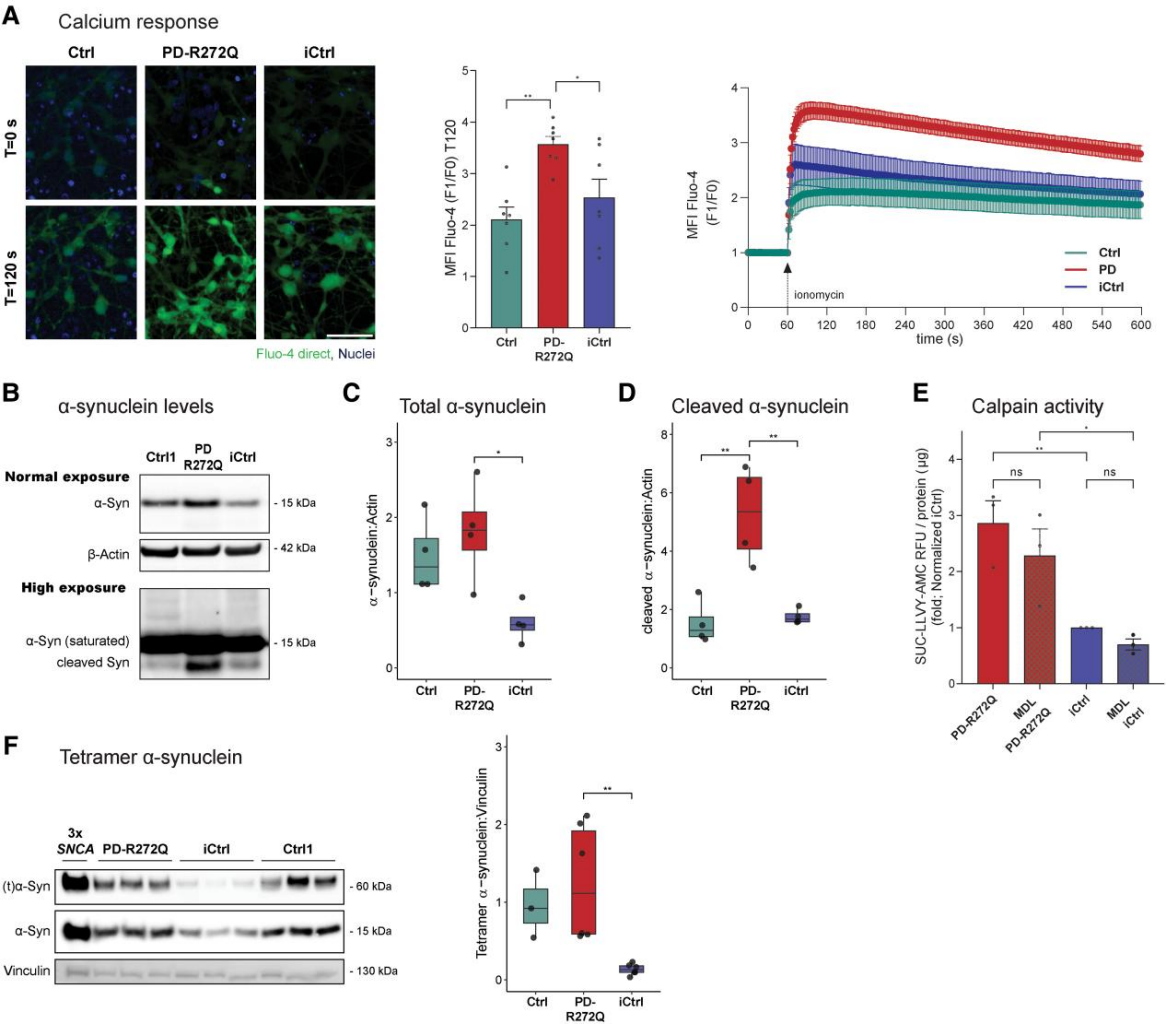
**p.R272Q Miro1 mutation promoted increased levels of α-synuclein toxic-prone forms via calcium-dependent activation of calpain**. (**A**) *Left*: Representative images of dopaminergic neurons containing Fluo-4 direct (green) and Hoechst (blue, nuclei) before (0 s) and after (120 s) ionomycin injection. Scale bar: 50 µm. *Middle*: Mean fluorescence intensity (MFI) of Fluo-4 direct signal (F1) divided by its basal signal (F0) at 120 s (T120). *Right*: Graphical representation of F1/F0 MFI through time assessed by live imaging. *n* = 7 independent derivations. (**B**) Representative image of monomeric α-synuclein (Syn, 15 kDa) and housekeeping protein β-actin (42 kDa) western blotting in dopaminergic neurons at non-saturated exposure (*top bands*), and a representative image of lower molecular weight α-synuclein band (<15 kDa) after high exposure time (*bottom band*). (**C**) Graphic showing quantification of monomeric α-synuclein (15 kDa). *n* = 4 independent derivations. (**D**) Quantification of α-synuclein cleaved species (<15 kDa). *n* = 4 independent derivations. (**E**) Calpain activity in PD-R272Q and iCtrl neurons measured using the *N*-succinyl-Leu-Leu-Val-Tyr-7-amido-4-methylcoumarin (Suc-LLVY-AMC) fluorescence probe in the presence or absence of the reversible calpain inhibitor MDL-28170 (MDL). Data represented as relative fluorescent units per µg of protein, normalized to iCtrl. *n* = 3 independent derivations. (**F**) *Left*: Representative image of high molecular weight form of α-synuclein, possible tetramer form [60 kDa, (t)α-syn], and housekeeping protein vinculin (130 kDa) western blotting in dopaminergic neurons. A qualitative control based on lysates of patient-specific induced pluripotent stem cell (iPSC)-derived dopaminergic neurons carrying a triplication in the *SNCA* gene (3x*SNCA*) is shown in the first lane. *Right*: Quantification of 60 kDa (t)α-syn. *n* = 3–6 independent derivations. Data presented as median with maximum/minimum. *P < 0.05, **P < 0.01, using one-way ANOVA with a *post hoc* Tukey’s Honest Significant Difference test (**A**–**D** and **F**) or two-way ANOVA (**E**). Full membranes are displayed in the Supplementary material, ‘Western blotting data’ section. Ctrl = control; iCtrl = isogenic control; PD = Parkinson’s disease.

Recent reports have pointed to defective calcium homeostasis in p.R272Q Miro1 dopaminergic neurons.^13,32^ Calcium dysregulation is a primary feature underlying α-synuclein aggregation and toxicity, which can precede oxidative stress and mitochondrial damage.^33,34^ To assess the neuronal response to increased calcium levels, Fluo-4 Direct MFI was measured over time in the presence of ionomycin. In this setting, PD-R272Q dopaminergic neurons showed a higher increase in the F1/F0 ratio compared to both controls throughout the entire assay duration (Fig. 4A and Supplementary Fig. 6A), indicative of defective calcium handling capacity. Transcriptomic data showed upregulation of *SNCA* transcript in PD-R272Q neurons (Supplementary Fig. 6B). In accordance, α-synuclein protein levels were significantly higher in PD-R272Q dopaminergic neurons compared to iCtrl but not to Ctrl (Fig. 4B and C and Supplementary Fig. 6C). Remarkably, we observed a fast-migrating α-synuclein band, possibly corresponding to a cleaved form of α-synuclein, present at significantly higher levels in PD-R272Q compared to healthy and isogenic controls (Fig. 4B and D). These results suggest that p.R272Q Miro1 interferes with α-synuclein structural forms and/or protein levels. To better understand the possible mechanisms behind p.R272Q Miro1-dependent α-synuclein accumulation, we investigated calpains’ enzymatic activity. Calpain protease activity promotes α-synuclein cleavage in the presence of calcium.^35^ We observed a significant increase in calpain activity in PD-R272Q neurons, as revealed by the SUC-LLVY-AMC (Fig. 4E) and SUC-LLV-AMC probes (Supplementary Fig. 6E), and by the significant increase of CDK5 protein levels, a downstream target of calpain (Supplementary Fig. 6F). Accumulation of the 60 kDa α-synuclein, possibly a tetramer form,^36,37^ was also observed in PD-R272Q neurons compared to the iCtrl, but not the Ctrl (Fig. 4F). Taken together, these findings are indicative of a calcium-induced calpain-mediated cleavage of α-synuclein in p.R272Q Miro1 neurons, which together with accumulation of oligomeric forms of α-synuclein, might contribute to mitochondrial damage.

### Increased dopaminergic neuronal cell death in p.R272Q Miro1 midbrain organoids

**Figure 5:**
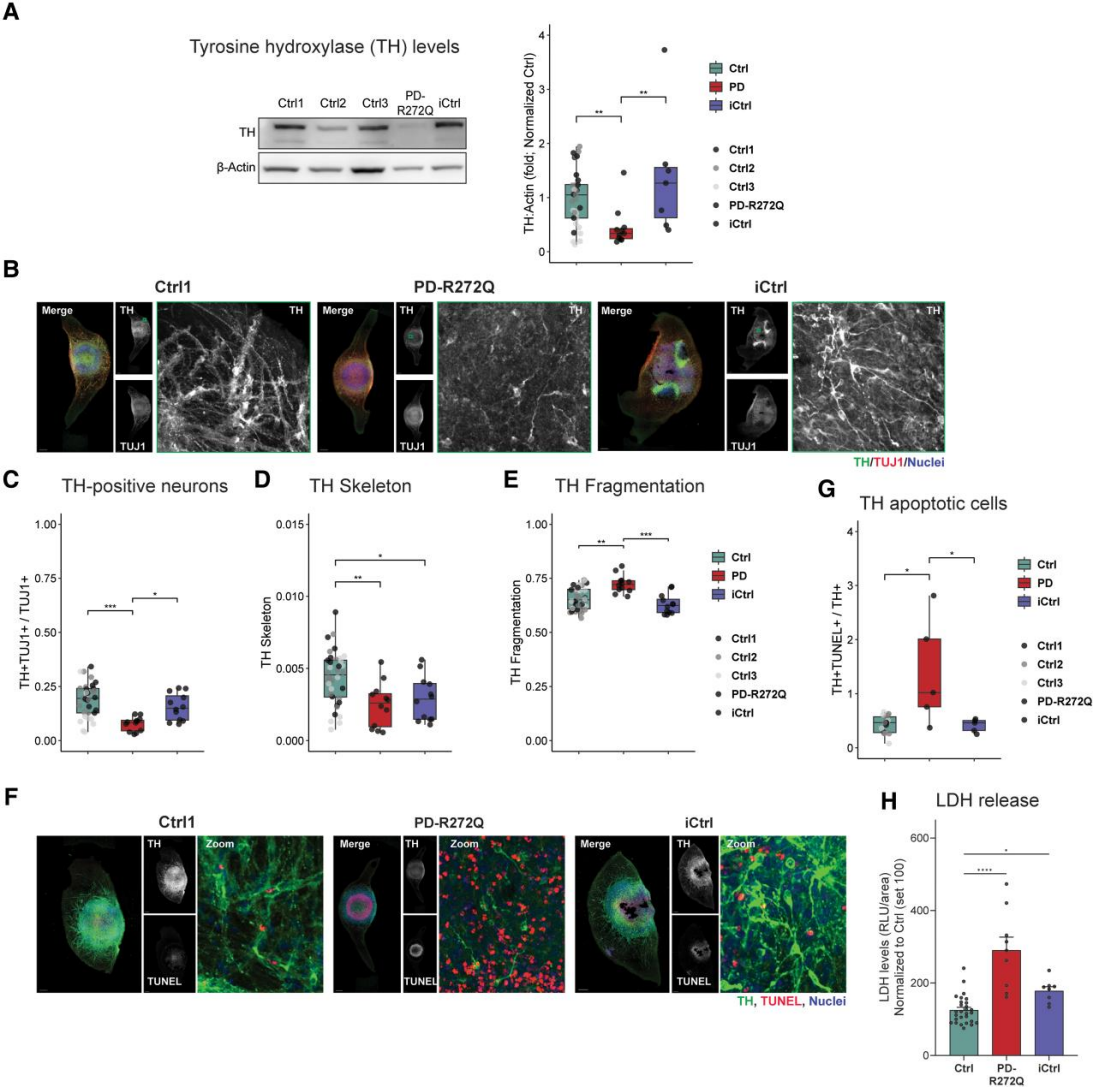
**p.R272Q Miro1 mutant midbrain organoids showed signs of dopaminergic neuron loss.** (**A**) Tyrosine hydroxylase (TH) representative image (*right*) and quantification (*left*) evaluated in midbrain organoids by western blotting (TH: 60 kDa; β-actin: 42 kDa). Ctrl *n* = 30, PD-R272Q *n* = 10, iCtrl *n* = 6 from 6 (iCtrl) or 10 (Ctrl, PD) independent derivations. Full membranes in the Supplementary material, ‘Western blotting data’ section. (**B**) Immunofluorescent analysis of TH-positive (TH+) signal, total neural marker TUJ1 and nuclei (Hoechst) in midbrain organoids. Scale bar: 200 µm. (**C**) Graphic displays the quantification of the total volume occupied by the TH and TUJ1 double-positive signal within the total TUJ1. (**D** and **E**) Immunofluorescent TH-based morphometric features (**D**) 3D skeleton and (**E**) fragmentation index. (**C** and **D**) *n* = 12–36 from five independent derivations. (**F**) Immunofluorescent identified TH+ neurons (green) undergoing apoptosis (TUNEL assay, red). Nuclei are shown in blue. Scale bar: 200 µm. (**G**) Box plot depicts the volume of TH neurons undergoing apoptosis (TH+, TUNEL+) normalized by the total TH. *n* = 7–16 organoids from three independent derivations. (**H**) Quantification of the relative abundance of lactate dehydrogenase (LDH) released into midbrain organoids media. *n* = 8–28 from three independent derivations. All data are presented as median with maximum/minimum or mean ± standard error of the mean. *P < 0.05, **P < 0.01, ***P < 0.001, ****P < 0.0001 using non-parametric multiple comparison Kruskal–Wallis test (**A**, **E**, **G** and **H**) or one-way ANOVA with a *post hoc* Tukey’s Honest Significant Difference test (**C** and **D**). Ctrl = control; iCtrl = isogenic control; PD = Parkinson’s disease; TUNEL = terminal deoxynucleotidyl transferase dUTP nick-end labelling.

The loss of dopaminergic neurons is a key hallmark of PD. Immunoblotting analyses showed a significant reduction of the dopaminergic marker TH in PD-R272Q midbrain organoids compared with both controls (Fig. 5A). Similar results were obtained by confocal microscopy, upon quantification of the TH-positive (TH+) signal within the total neuronal population (TUJ1+; Fig. 5B and C). High content image morphometric analysis^21,22^ revealed that, in mutant organoids, TH+ neurons have a reduced average length (skeleton) compared to Ctrl, but not to iCtrl (Fig. 5B and D), meaning that TH+ PD-R272Q neurons have on average a smaller cytoskeleton. Moreover, the neurite fragmentation index of TH+ neurons, which is an early indicator of neurodegeneration,^38^ was significantly increased in PD-R272Q organoids (Fig. 5B and E). In line, scRNAseq analysis showed clear deregulation of genes related to apoptosis within the dopaminergic neuron clusters (Supplementary Fig. 7A). The presence of LDH in the organoid media and the TUNEL apoptotic assay were used to assess cell viability (Fig. 5F–H). Thirty-day-old PD-R272Q organoids showed a significant increase in LDH abundance (Fig. 5H) and higher levels of DNA fragmentation (Fig. 5F and G) within the dopaminergic neuron population, compared to both controls. Notably, 20-day-old PD-R272Q organoids had similar total neurons (MAP2), TH+ cells and TH+ apoptotic neurons than both controls, although TH fragmentation was significantly augmented compared with Ctrl (Supplementary Fig. 7B–E). Altogether, these findings suggested a selective loss of TH+ neurons in Miro1 mutant organoids at Day 30, mediated, at least in part, by apoptosis.

### Aged p.R285Q Miro1 knock-in mice showed dopaminergic neuronal degeneration and behavioural alterations

**Figure 6:**
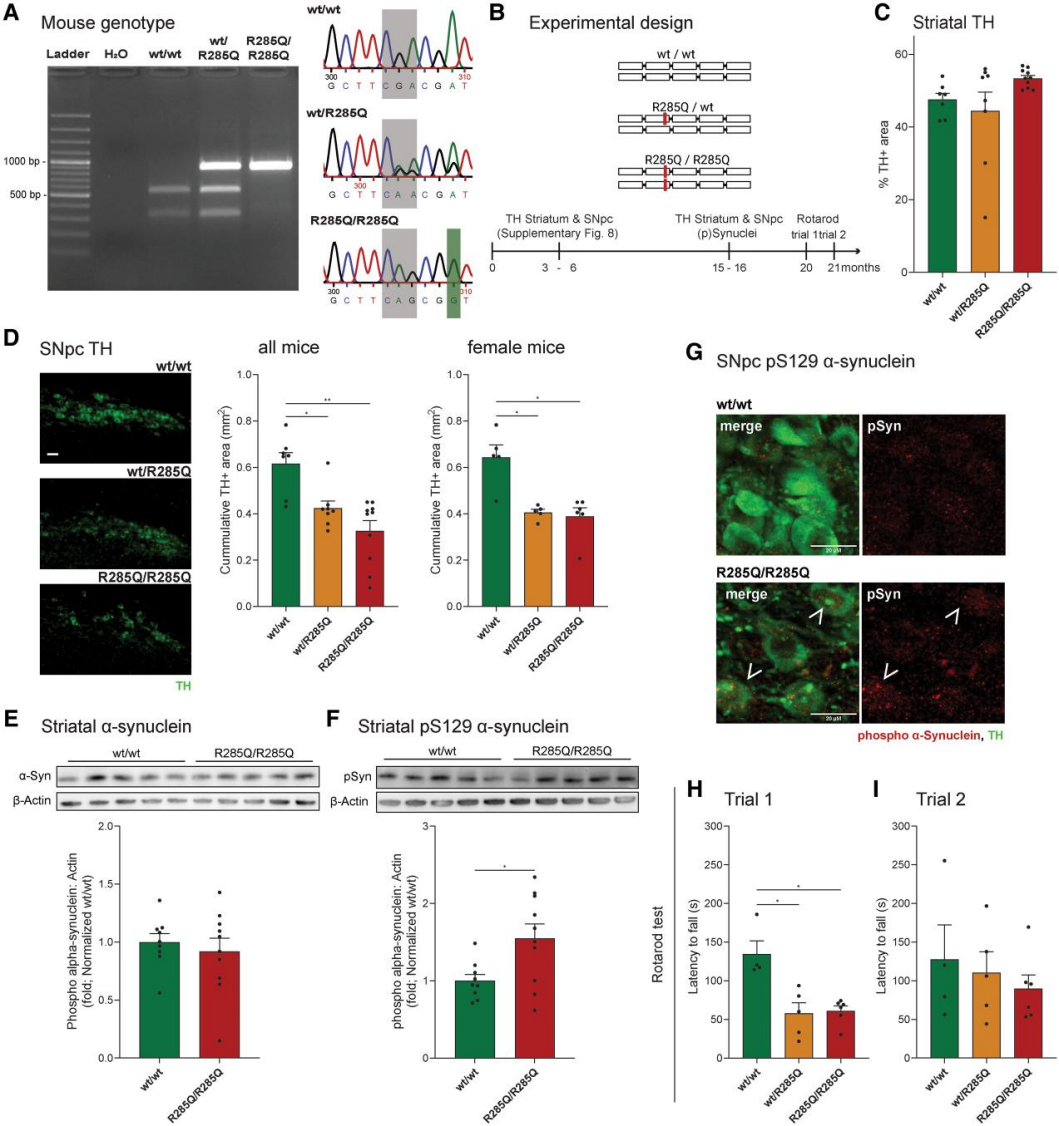
**p.R285Q Miro1 mutant aged mice presented dopaminergic neuron loss and behaviour alterations.** (**A**) Agarose gel (*left*) and DNA sequencing (*right*) confirming the successful integration of the Miro1 point mutation. (**B**) Scheme illustrating the timeline of the experiments and the different mouse genotypes: wild-type mice (wt/wt), heterozygous (wt/R285Q) and homozygous (R285Q/R285Q). (**C**) Graphic depicts the percentage of striatal tyrosine hydroxylase-positive (TH+) area. (**D**) *Left*: Representative image of substantia nigra par compacta (SNpc) TH immunoreactivity. Scale bar: 50 µm. Quantification of the area occupied by TH in SNpc in 15-month-old mice (*middle*) or female only (*right*). (**C** and **D**) wt/wt: *n* = 2 males + 5 females; wt/R285Q: *n* = 3 males + 5 females; R285/R285Q: *n* = 4 males + 6 females. (**E** and **F**) Western blotting quantification (*bottom*) and representative image (*top*) of monomeric α-synuclein (**E**) and phosphorylated (p)S129 α-synuclein (**F**) in the striatum of female mice. wt/wt *n* = 9, R285Q/R285Q *n* = 10. Full membranes in the Supplementary material, ‘Western blotting data’ section. (**G**) Representative confocal images of pS129 α-synuclein (red) and TH-positive (green) signal in the SNpc of female wild-type (*top*) and homozygous (*bottom*) mice. Arrows depict pS129 α-synuclein intracellular inclusions. Scale bar: 20 µm. (**H** and **I**) Bar graph displays female mice latency to fall in seconds upon the first (**H**) and second (**I**) trials of the Rotarod behaviour test at 20 and 21 months, respectively. wt/wt: *n* = 4; wt/R285Q: *n* = 5; R285/R285Q: *n* = 6. Graphs are represented as mean ± standard error of the mean; each point represents one mouse. *P < 0.05, **P < 0.01 using non-parametric multiple comparison Kruskal–Wallis test (**C**, **G** and **H**) or unpaired *t*-test (**D** and **E**).

The Miro1 protein is evolutionarily conserved among species.^4^ The R285 amino acid in mice is the equivalent of the human R272 residue found mutated in the PD patient. Thus, to study the potential contribution of mutant Miro1 to PD pathogenesis *in vivo*, we generated a knock-in mouse model by introducing the corresponding p.R285Q Miro1 mutation using CRISPR/Cas9 technology (Supplementary Fig. 8A). Successful generation of knock-in mice was validated by PCR and DNA sequencing analysis in wild-type animals (wt/wt), as well as in heterozygous (wt/R285Q) and homozygous (R285Q/R285Q) mutant mice (Fig. 6A). Miro1 mutation did not impact mouse bodyweight (Supplementary Fig. 8B). In young mice (3 to 6-month-old; Fig. 6B), immunohistochemical analysis showed similar amount of TH+ terminals and dopamine transporter (DAT) in the striatum, and similar TH levels in the SNpc of the three groups (Supplementary Fig. 8C–E). In 15-month-old mice, striatal TH (Fig. 6C), DAT levels and dopamine (Supplementary Fig. 8F–G) were also unaffected. However, 15-month-old mutant mice showed a significant decrease in the SNpc TH area, reflecting dopaminergic neuron loss in both heterozygous and homozygous mice, an effect that reached significance in females (Fig. 6D and Supplementary Fig. 8H), but not in males, which could be due to a sample size limitation or sex-specific, i.e. genetic or hormonal, differences. Total levels of α-synuclein in the striatum of old mice did not differ among the different genotypes (Fig. 6E). Nevertheless, old homozygous mutant mice showed significantly more phosphorylated S129 α-synuclein (Fig. 6F). The presence of phosphorylated S129 α-synuclein inclusions was also qualitatively observed via immunofluorescence in the mice SNpc (Fig. 6G). Assessment of mitochondrial protein TOM20 in SNpc TH+ neurons showed no significant alterations in any genotype or age (Supplementary Fig. 9A and B). Nevertheless, a tendency to accumulate TOM20 in old homozygous mice was observed. Following on this, striatal mitochondrial-related protein levels were assessed by western blotting (Supplementary Fig. 9C–F). TOM20 levels were also slightly increased in Miro1 homozygous mice (*P* = 0.055; Supplementary Fig. 9C), whereas the mitochondrial matrix MnSOD protein was significantly upregulated (*P* = 0.044; Supplementary Fig. 9D). However, when assessing the expression of mitochondrial OXPHOS complex subunits, no significant differences were observed (Supplementary Fig. 9F), indicating a marginal impact of Miro1 mutation on mitochondrial mass and respiratory chain composition. Notably, autophagy-related protein p62 was significantly downregulated in the R285Q/R285Q mice (Supplementary Fig. 9E), suggesting an active autophagy/mitophagy, which could be interpreted as a compensatory mechanism Miro1 mutant neurons put in place to eliminate dysfunctional mitochondria.

Twenty-month-old mice were monitored for water and food consumption as well as global activity, using Phenomaster cages, and no significant differences were observed (Supplementary Fig. 8I–K). Then, the Rotarod test was used to evaluate spontaneous motor activity^39^ and/or anterograde procedural memory^40^ in 20- (trial 1) and 21- (trial 2) month-old female mice. Heterozygous and homozygous p.R285Q Miro1 mice displayed a significantly reduced latency to fall compared to wild-type mice in their first trial (Fig. 6H), but not in the second trial (Fig. 6I), which is indicative of compromised motor-learning processes. Taken together, the characterization of Miro1 mutant mice suggested a pathological role of p.R285Q (human p.R272Q) point mutation in PD etiopathogenesis.

## Discussion

PD is not only a clinically but also genetically heterogenous disease. Besides rare Mendelian mutations in monogenic forms and common variants in genes associated with the common sporadic form of PD, there is growing evidence pointing to an important role of low-frequency variants with substantial effect in PD pathogenesis.^41^ Recently, a large study investigating the burden of rare coding genetic variants in PD patients based on their predicted functional impact identified *RHOT1* as one of the genes with the highest burden (3rd of 2500 genes).^42^ This strongly supports a role for rare variants in the *RHOT1* gene, encoding Miro1, in the genetic susceptibility to PD, including those previously identified by us.^11,12^ Furthermore, a common molecular signature, where pathological stabilization of Miro1 caused impaired mitochondrial clearance, has been identified in both familial (*PINK1*, *PRKN*, *SNCA*, *LRRK2*) and sporadic PD cases,^5,7,8^ highlighting Miro1 as a potential convergent player, as well as a potential molecular biomarker in PD.^43^ Due to limited pedigree sizes in central Europe, the genetic impact of *RHOT1* variants was not accessible for validation via classical Mendelian co-segregation patterns, urging the need for large-scale sequencing approaches^42^ and functional characterization in disease-relevant models. Herein, a novel knock-in mouse model in combination with patient-based iPSC-derived models, including a gene-corrected control, allowed for dissecting neuronal phenotypes related to the p.R272Q Miro1 mutation and their impact on PD pathogenesis. We demonstrated that p.R272Q Miro1 mutation causes mitochondrial dysfunction, leading to dopaminergic neuron loss, both *in vitro* and *in vivo*. Importantly, we unveiled Miro1-dependent cellular processes and molecular signatures, providing convincing evidence supporting its role in neurodegeneration in PD.

*In vitro*, transcriptomic analyses in Miro1 p.R272Q mutant versus healthy or isogenic controls showed a mutation-specific deregulation on PD pathways, which are also known to be altered in sporadic and familial PD, such as LRRK2 and Tau, oxidative stress, iron homeostasis and apoptosis.^44-46^ For example, *UBL5* (ubiquitin-related) and *FTL* (iron-related) have previously been linked to Miro1-mediated mitophagy regulation^4^ and to an iron-calcium-Miro axis,^47^ respectively. The iron-calcium-Miro1 axis hypothesis states that elevated iron levels in PD lead to mitochondrial calcium overflow, which might be preceded by reduced calcium sensing Miro1 capability.^47^ Reduction of FTL protein levels in mutant organoids further supports possible calcium and ROS alterations. Miro1 is not only able to sense cytosolic calcium^48^ but also oxidative stress,^49,50^ with impairments in Miro1 leading to mitophagy alterations and bioenergetics deficits.^47,49,50^ Accordingly, we observed high levels of ROS and a reduced number of functional mitochondria in the PD-R272Q condition. Moreover, mitochondrial respiration and energy production were impaired in Miro1 mutant midbrain organoids and dopaminergic neurons (i.e. OCR, NAD and FAD abundance as well as metabolic alterations). The altered metabolism aligned to the increased mitochondrial gene expression in mutant organoids further suggest an attempt of PD-R272Q neurons and organoids to compensate for ATP and/or NAD diminished levels. Moreover, the low abundance of oxidized co-factors NAD and FAD point to possible reoxidation impairments, which support disruptions in the electron transport chain. Notably, PD-R272Q dopaminergic neurons showed a tendency to present lower NAD and NADH abundance, suggesting energy deficits might come from the low availability of these species rather than their imbalance. NAD depletion has been reported in ageing and neurodegeneration models, with implications on the accumulation of damaged mitochondria, mitochondria bioenergetics, oxidative stress and neuronal survival.^51^ Furthermore, mitochondrial metabolic impairments and a lower NAD pool were reported in iPSC-derived neural precursors obtained from idiopathic PD patients,^17^ with NAD+ supplementation showing neuroprotective properties in PD patients.^52,53^ Concomitantly, a recent study in 20-day-old iPSC-derived dopaminergic neurons from a healthy individual in which the heterozygous p.R272Q Miro1 mutation had been introduced artificially showed altered mitochondrial morphology and mitochondrial respiration, including basal respiration, as well as calcium deregulation.^32^ Herein, patient-specific PD-R272Q dopaminergic neurons also showed calcium handling dysregulation,^13^ which might contribute to the observed mitochondrial defects. Importantly, depending on the model (organoids or dopaminergic neurons), phenotypes varied slightly (e.g. seahorse spare respiratory capacity, and MMP impairment observed in organoids already at a basal state). These differences could be explained by the cellular complexity of midbrain organoids, assuming a potential role, albeit different, of Miro1 in other cell types, such as astrocytes and/or GABAergic neurons, which are less energy-demanding than dopaminergic neurons.^54, 55^ Potential compensatory mechanisms from non-mitochondrial metabolic pathways, including fatty acid oxidation in astrocytes might justify energetic differences observed under stress conditions. Notwithstanding, the Miro1 EF-hand calcium-binding domain is required for activity-driven mitochondria positioning at tripartite synapses, therefore alterations in Miro1 might affect gliotransmission (astrocyte neurotransmitter release).^56^ In GABAergic neurons, Miro1 loss also leads to defective mitochondrial trafficking and distribution.^57^ Of note, p.R272Q Miro1 isogenic control showed in diverse assays an intermediate or differentiated response when compared to healthy controls, suggesting that additional factors defining the individual ‘genetic background’ may influence the occurrence of particular phenotypes.^58^

α-Synuclein has recently been implicated in mechanisms regulating mitochondrial quality control and dynamics.^59^ Moreover, α-synuclein accumulation and calcium deregulation were demonstrated to be early and persistent pathological phenotypes observed in patient-specific PD neurons carrying *SNCA* mutation.^34^ In this study, PD-R272Q dopaminergic neurons showed individual-specific monomeric and oligomeric α-synuclein upregulation. Previously, a positive correlation between Miro1 and α-synuclein levels was shown in the SNpc of sporadic PD patients,^8^ further supporting a possible functional link between both proteins. This link might be related to altered mitochondrial quality control mechanisms^59^ or Miro1-dependent calcium deregulation since disruption in intracellular calcium buffering promotes α-synuclein aggregation.^60^ Calcium binding to the NAC domain of α-synuclein leads to its exposure, promoting the formation of aggregates.^61^ On top, increased intracellular calcium levels have been shown to increase calpain activity, a calcium-dependent protease family capable of cleaving α-synuclein at different residues.^35^ Herein, we showed a p.R272Q Miro1 mutation-specific increase in calpain activity, most likely due to the observed mutation-dependent calcium deregulation. In addition, PD-R272Q neurons display a significant increase of a protein band compatible with cleaved α-synuclein compared with healthy and isogenic controls. This suggests a calcium-induced calpain-mediated cleavage of α-synuclein that is not, or only partially, responsible for formation of the observed oligomeric α-synuclein. Low molecular weight truncated forms of α-synuclein have been found upregulated in the brain of Lewy body dementia patients,^62^ and described as relevant for spreading and aggregation.^63^ Others showed that calcium-dependent calpain activation results in higher generation of α-synuclein cleaved forms, promoting the formation of α-synuclein high molecular species and adoption of the aggregation-prone β-sheet conformation, culminating in elevated levels of aggregated α-synuclein.^35, 64, 65^ These results unveil a novel molecular mechanism linking Miro1 and pathological α-synuclein accumulation based on altered calcium homeostasis due to Miro1 mutation and consequent calpain activation. Of note, pathological calpain activation also interferes with other important pathways, promoting, for example, mitochondrial dysfunction and apoptosis.^66^ Altogether, these alterations might result in cell demise. Indeed, p.R272Q Miro1 mutant organoids showed a specific loss of TH+ dopaminergic neurons at Day 30 of culture compared with healthy and isogenic controls. The dopaminergic neuron population within midbrain organoids produces dopamine and has pacemaker activity.^15^ These features can justify the higher vulnerability of the dopaminergic neuron population to stress and energy loss driven by the p.R272Q Miro1 mutation within organoids, modelling the main hallmark of PD.^2^ Likewise, the loss of TH+ neurons in the midbrain organoid model has also been described in familial forms of PD, including *LRRK2*, *PINK1* and *GBA* mutations.^19, 20, 27^

*In vivo*, we generated the first Miro1 mutant knock-in mouse model expressing physiological levels of mutant p.R285Q Miro1, an orthologue of the human PD-linked variant. p.R285Q Miro1 mice presented significant dopaminergic neuronal loss in the SNpc of aged animals, corroborating the midbrain organoid findings. Old homozygous mice also showed increased levels of mitochondrial proteins TOM20 and MnSOD, and reduction of p62, an autophagy/mitophagy-related protein, indicating a possible alteration in the mitochondrial quality control system. The role of p62 on mitochondrial function extends beyond mitophagy. Knock-down of p62 in iPSC-derived neurons resulted in mitochondrial gene expression and impaired mitochondrial respiration under stress conditions, without affecting the overall autophagy/mitophagy process.^63^ Of note, these mice presented elevated levels of phosphorylated S129 α-synuclein, a post-translational modification associated with α-synuclein toxicity and aggregation,^67^ which affect mitochondrial function.^59^ However, additional studies are needed to dissect the molecular determinants linking decreased p62 levels to phosphorylated S129 α-synuclein accumulation, TH loss and behaviour defects in this mouse model. Mutant p.R285Q mice spent significantly less time on the rod during the first trial of the Rotarod task, which might be due to difficulties in performing precise movements^39^ or impaired anterograde procedural memory.^40^ Dodson and colleagues^68^ showed that SNpc dopaminergic neurons stop firing at movement onset, leading to slight impairment in motor precision in humanized *SNCA* overexpression and *SNCA* knockout mice models. PD patients show decreased implicit learning abilities concerning new motor tasks (anterograde procedural memory), while their motor performance improves over time on pursuit rotor-motor task tests.^40, 69^ Similarly, rotarod differences in p.R285Q Miro1 mice were dissipated on the second trial, further supporting involvement of learning processes or compensatory mechanisms related to motor deficits. Notably, the behavioural changes observed in our aged p.R285Q mice were accompanied by a reduced number of dopaminergic neurons in the SNpc but not in the density of their terminals in the striatum. Soto et al.^70^ recently reported age-related locomotor impairments in PINK1 knock-out rats dependent on SNpc, but not striatal, altered dopamine signaling. It is also important to notice that complete emulation of human PD characteristics might fall short due to species differences.

PD has been modelled by toxin-based acute challenges that, while recapitulating acute SNpc neuronal loss, do not replicate the typical chronic progressive neurodegeneration responsible for the pathological features of the human condition.^71^ Other PD mouse models, including single knock-out (e.g. *DJ-1, PINK1*, *PRKN*), triple *PINK1*/*PRKN*/*DJ-1* knockouts, or *LRKK2*-R1441G transgenic mice, showed no dopaminergic neurodegeneration albeit their motor coordination was altered.^71-74^ LRRK2 knock-in mice have also been used to model PD, showing subtle differences in striatal dopamine content and/or behaviour defects.^75^ However, dopaminergic degeneration was only observed in *LRKK2*-G2019S knock-in mice when combined with *SNCA*-A53T overexpression.^76^ Herein, we showed that physiological levels of p.R285Q Miro1 are sufficient to cause behaviour impairments and SNpc TH loss in mice in an age-dependent manner.

Notwithstanding, the study conclusions are limited by the fact only one p.R272Q Miro1 patient is accessible worldwide, and by the focus on one timepoint *in vitro*. Longitudinal studies might dissect the causes and consequences of Miro1 mutation. In-depth studies on the interplay between Miro1 and α-synuclein are needed to dissect genetic and/or species background-dependent effects from mutation-specific mechanisms. Moreover, confirmation of certain phenotypes, namely in terms of calcium response and affinity, mitochondria movement and mitophagy in all three models would allow us to better establish a direct mechanistic link between mitochondrial dysfunction and neuronal loss. Additionally, using electrophysiology and imaging in midbrain organoids and *in vivo*, coupled with the characterization of other PD hallmarks such as neuroinflammation and astrocytic involvement, could provide a more comprehensive view of p.R272Q Miro1-induced PD pathogenesis.

## Conclusion

The presented *in vitro* and *in vivo* models demonstrate that p.R272Q Miro1 mutation is sufficient to cause dopaminergic neuron loss, likely mediated through the alteration of mitochondrial status. Mechanistically, we demonstrated that p.R272Q Miro1 mutation disrupts intracellular calcium regulation, leading to calpain-dependent accumulation of α-synuclein. This accumulation may exacerbate mutation-dependent mitochondrial damage, ultimately resulting in the loss of dopaminergic neurons. Moreover, we emphasize the significance of rare pathogenic variants, such as those found in *RHOT1*, in PD pathogenesis, further demonstrating the importance of genetic variance in PD susceptibility. Altogether, these findings support Miro1 as a potential convergent biomolecule and drug target in both familial and sporadic cases of PD, highlighting its relevance for modelling PD phenotypes and developing new disease-modifying therapies.

## Supplementary Material

awaf051_Supplementary_Data

## Data Availability

All data, including raw and processed are publicly available at https://doi.org/10.17881/vm4y-sv50. New generated scripts are available on GitLab: https://gitlab.lcsb.uni.lu/dvb/saraiva_2023.
