# Supplementary material for: Parkinson’s disease mutant Miro1 causes mitochondrial dysfunction and dopaminergic neuron loss
Source: Brain. 2025 Feb 6;148(10):3607–22. doi: 10.1093/brain/awaf051 (PMC12493065; doi:10.1093/brain/awaf051)
Supplement: awaf051_Supplementary_Data [file awaf051_supplementary_data.zip › brain-2024-01618-File010.pdf]

Supplementary Figures

Parkinson's disease mutant Miro1 causes mitochondrial dysfunction and dopaminergic neuron loss

Axel Chemla[1], Giuseppe Arena[1], Ginevra Sacripanti[1], Kyriaki Barmpa[1], Alise Zagare[1], Pierre Garcia[1,2], Vyron Gorgogietas[1], Paul Antony[1], Jochen Ohnmacht[3], Alexandre Baron[1], Jaqueline Jung[4], Frida Lind-Holm Mogensen[3,5], Alessandro Michelucci[3], Anne-Marie Marzesco[1,§], Manuel Buttini[1,2], Thorsten Schmidt[4], Anne Grünewald[1,6], Jens C. Schwamborn[1,*], Rejko Krüger[1,3,7,*], Cláudia Saraiva[1,*]

Affiliations:

[1]Luxembourg Centre for Systems Biomedicine (LCSB), University of Luxembourg, L-4362 Esch-sur-Alzette, Luxembourg

[2]Luxembourg Center of Neuropathology (LCNP), Laboratoire National de Santé, L-3555, Dudelange, Luxembourg

[3] Luxembourg Institute of Health (LIH), L-1445 Luxembourg, Luxembourg

[4]Institute of Medical Genetics and Applied Genomics, University of Tübingen, 72076 Tübingen, Germany

[5]Faculty of Science, Technology and Medicine, University of Luxembourg, L-4365 Esch-sur-Alzette, Luxembourg

[6]Institute of Neurogenetics, University of Lübeck, 160 Lübeck, Germany

[7]Centre Hospitalier de Luxembourg, L-1210, Luxembourg, Luxembourg

[§] Current address: University Medical Center Hamburg-Eppendorf, Center for Molecular Neurobiology Hamburg, 20246 Hamburg, Germany

[*] Senior authors

Correspondence to: jens.schwamborn@uni.lu (JCS), rejko.krueger@lih.lu (RK) and claudia.saraiva@uni.lu (CS)

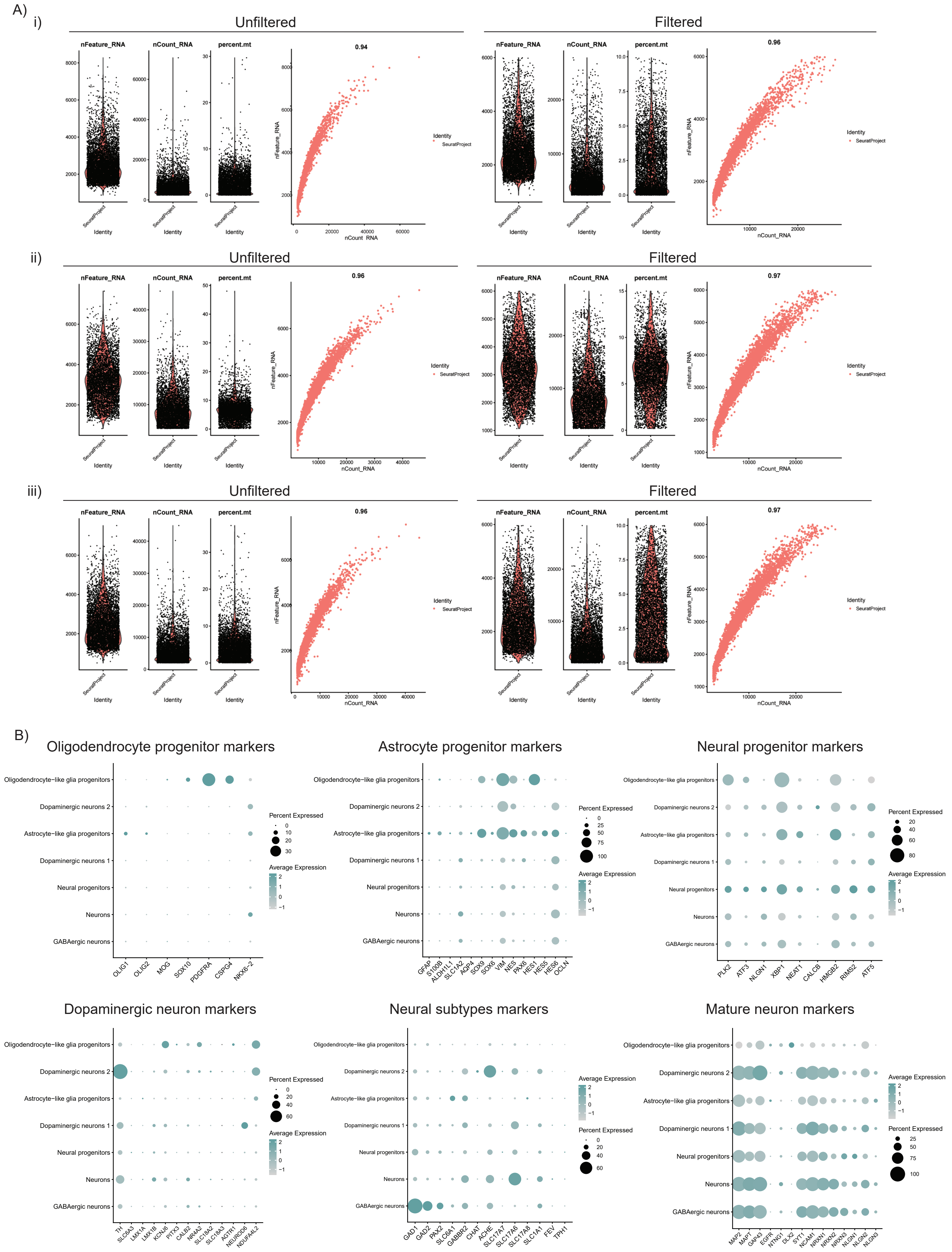
A)
i)
Unfiltered
Filtered
nFeature_RNA
nCount_RNA
percent.mt
0.94
0.96
Identity
SeuratProject
nFeature_RNA
nCount_RNA
ii)
Unfiltered
Filtered
0.96
0.97
iii)
Unfiltered
Filtered
0.96
0.97
B)
Oligodendrocyte progenitor markers
Astrocyte progenitor markers
Neural progenitor markers
Dopaminergic neuron markers
Neural subtypes markers
Mature neuron markers
Oligodendrocyte−like glia progenitors
Dopaminergic neurons 2
Astrocyte−like glia progenitors
Dopaminergic neurons 1
Neural progenitors
Neurons
GABAergic neurons
Percent Expressed
Average Expression
OLIG1 OLIG2 MOG SOX10 PDGFRA CSPG4 NKX6−2
GFAP S100B ALDH1L1 SLC1A2 AQP4 SOX9 SOX6 VIM NES PAX6 HES1 HES5 HES6 OCLN
PLK2 ATF3 NLGN1 XBP1 NEAT1 CALCB HMGB2 RIMS2 ATF5
TH SLC6A3 LMX1A LMX1B KCNJ6 PITX3 CALB2 NR4A2 SLC18A2 SLC18A3 AGTR1 NEUROD6 NDUFA4L2
GAD1 GAD2 PAX2 SLC6A1 GABBR2 CHAT ACHE SLC17A7 SLC17A6 SLC17A8 SLC1A1 FEV TPH1
MAP2 MAPT GAP43 EGFR NTNG1 DLX2 SYT1 NCAM1 NRXN1 NRXN2 NRXN3 NLGN1 NLGN2 NLGN3

Supplementary Figure 1 Midbrain organoid single-cell RNA sequencing quality control and cell cluster markers. (**A**) Plots representing quality control metrics including the gene number (nFeature_RNA), total molecules number (nCount_RNA), mitochondrial gene percentage, and correlation between the number of genes and total molecules before (unfiltered; left) and after (filtered; right) applying the quality control thresholding in (**i**) healthy midbrain organoids (Ctrl2), (**ii**) p.R272Q Miro1 mutant organoids (PD-R272Q), and (**iii**) isogenic control (iCtrl). (**B**) Expression levels of cell-specific markers for oligodendrocyte progenitors, glia progenitors, neural progenitors, dopaminergic neurons, other neuronal subtypes and mature neurons, respectively, in the different midbrain organoids clusters identified.

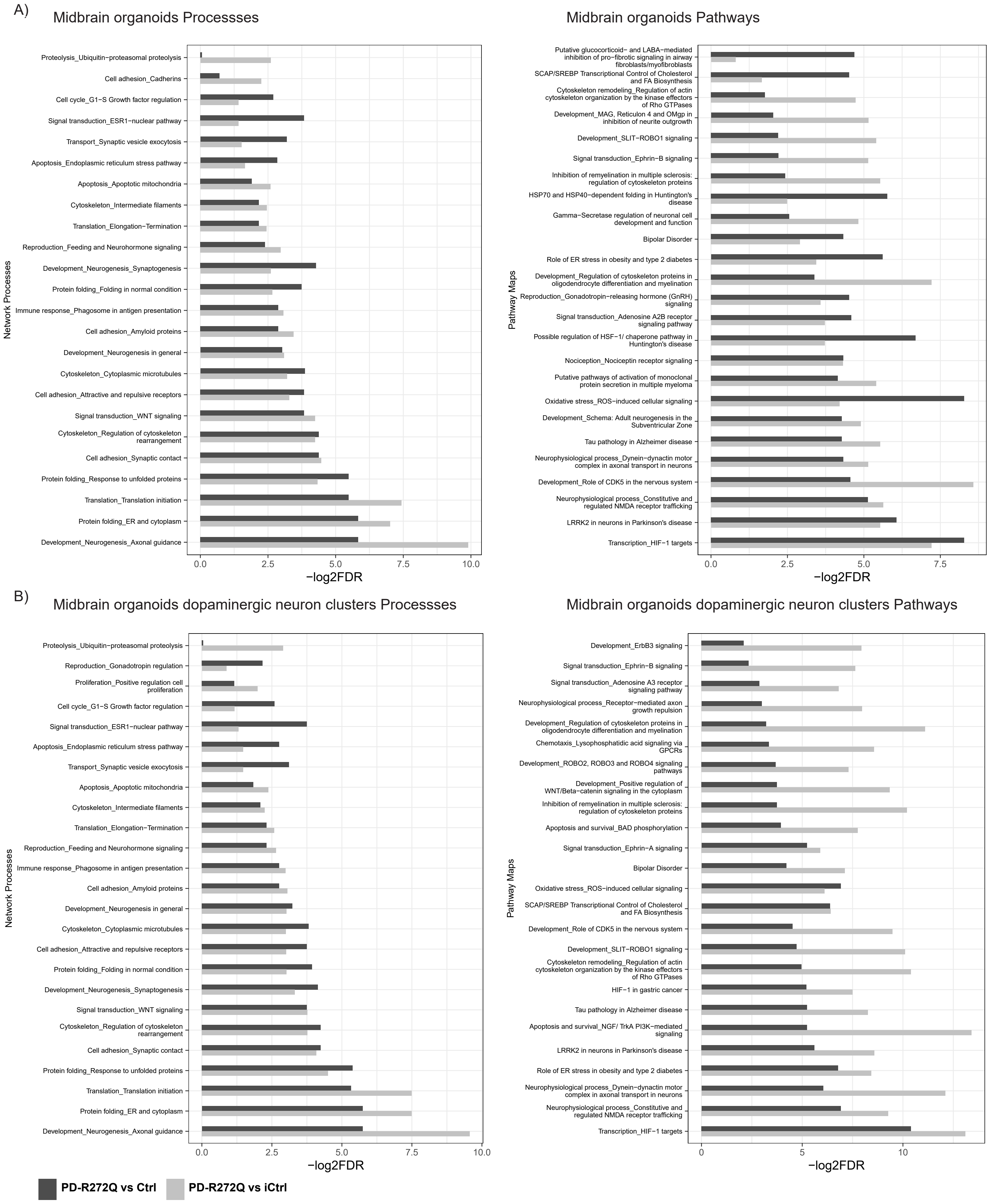
A)
Midbrain organoids Processses
Proteolysis_Ubiquitin−proteasomal proteolysis
Cell adhesion_Cadherins
Cell cycle_G1−S Growth factor regulation
Signal transduction_ESR1−nuclear pathway
Transport_Synaptic vesicle exocytosis
Apoptosis_Endoplasmic reticulum stress pathway
Apoptosis_Apoptotic mitochondria
Cytoskeleton_Intermediate filaments
Translation_Elongation−Termination
Reproduction_Feeding and Neurohormone signaling
Development_Neurogenesis_Synaptogenesis
Protein folding_Folding in normal condition
Immune response_Phagosome in antigen presentation
Cell adhesion_Amyloid proteins
Development_Neurogenesis in general
Cytoskeleton_Cytoplasmic microtubules
Cell adhesion_Attractive and repulsive receptors
Signal transduction_WNT signaling
Cytoskeleton_Regulation of cytoskeleton rearrangement
Cell adhesion_Synaptic contact
Protein folding_Response to unfolded proteins
Translation_Translation initiation
Protein folding_ER and cytoplasm
Development_Neurogenesis_Axonal guidance
Network Processes
0.0
2.5
5.0
7.5
10.0
−log2FDR
Midbrain organoids Pathways
Putative glucocorticoid− and LABA−mediated inhibition of pro−fibrotic signaling in airway fibroblasts/myofibroblasts
SCAP/SREBP Transcriptional Control of Cholesterol and FA Biosynthesis
Cytoskeleton remodeling_Regulation of actin cytoskeleton organization by the kinase effectors of Rho GTPases
Development_MAG, Reticulon 4 and OMgp in inhibition of neurite outgrowth
Development_SLIT−ROBO1 signaling
Signal transduction_Ephrin−B signaling
Inhibition of remyelination in multiple sclerosis: regulation of cytoskeleton proteins
HSP70 and HSP40−dependent folding in Huntington's disease
Gamma−Secretase regulation of neuronal cell development and function
Bipolar Disorder
Role of ER stress in obesity and type 2 diabetes
Development_Regulation of cytoskeleton proteins in oligodendrocyte differentiation and myelination
Reproduction_Gonadotropin−releasing hormone (GnRH) signaling
Signal transduction_Adenosine A2B receptor signaling pathway
Possible regulation of HSF−1/ chaperone pathway in Huntington's disease
Nociception_Nociceptin receptor signaling
Putative pathways of activation of monoclonal protein secretion in multiple myeloma
Oxidative stress_ROS−induced cellular signaling
Development_Schema: Adult neurogenesis in the Subventricular Zone
Tau pathology in Alzheimer disease
Neurophysiological process_Dynein−dynactin motor complex in axonal transport in neurons
Development_Role of CDK5 in the nervous system
Neurophysiological process_Constitutive and regulated NMDA receptor trafficking
LRRK2 in neurons in Parkinson's disease
Transcription_HIF−1 targets
Pathway Maps
0.0
2.5
5.0
7.5
−log2FDR
B)
Midbrain organoids dopaminergic neuron clusters Processses
Proteolysis_Ubiquitin−proteasomal proteolysis
Reproduction_Gonadotropin regulation
Proliferation_Positive regulation cell proliferation
Cell cycle_G1−S Growth factor regulation
Signal transduction_ESR1−nuclear pathway
Apoptosis_Endoplasmic reticulum stress pathway
Transport_Synaptic vesicle exocytosis
Apoptosis_Apoptotic mitochondria
Cytoskeleton_Intermediate filaments
Translation_Elongation−Termination
Reproduction_Feeding and Neurohormone signaling
Immune response_Phagosome in antigen presentation
Cell adhesion_Amyloid proteins
Development_Neurogenesis in general
Cytoskeleton_Cytoplasmic microtubules
Cell adhesion_Attractive and repulsive receptors
Protein folding_Folding in normal condition
Development_Neurogenesis_Synaptogenesis
Signal transduction_WNT signaling
Cytoskeleton_Regulation of cytoskeleton rearrangement
Cell adhesion_Synaptic contact
Protein folding_Response to unfolded proteins
Translation_Translation initiation
Protein folding_ER and cytoplasm
Development_Neurogenesis_Axonal guidance
Network Processes
0.0
2.5
5.0
7.5
10.0
−log2FDR
Midbrain organoids dopaminergic neuron clusters Pathways
Development_ErbB3 signaling
Signal transduction_Ephrin−B signaling
Signal transduction_Adenosine A3 receptor signaling pathway
Neurophysiological process_Receptor−mediated axon growth repulsion
Development_Regulation of cytoskeleton proteins in oligodendrocyte differentiation and myelination
Chemotaxis_Lysophosphatidic acid signaling via GPCRs
Development_ROBO2, ROBO3 and ROBO4 signaling pathways
Development_Positive regulation of WNT/Beta−catenin signaling in the cytoplasm
Inhibition of remyelination in multiple sclerosis: regulation of cytoskeleton proteins
Apoptosis and survival_BAD phosphorylation
Signal transduction_Ephrin−A signaling
Bipolar Disorder
Oxidative stress_ROS−induced cellular signaling
SCAP/SREBP Transcriptional Control of Cholesterol and FA Biosynthesis
Development_Role of CDK5 in the nervous system
Development_SLIT−ROBO1 signaling
Cytoskeleton remodeling_Regulation of actin cytoskeleton organization by the kinase effectors of Rho GTPases
HIF−1 in gastric cancer
Tau pathology in Alzheimer disease
Apoptosis and survival_NGF/ TrkA PI3K−mediated signaling
LRRK2 in neurons in Parkinson's disease
Role of ER stress in obesity and type 2 diabetes
Neurophysiological process_Dynein−dynactin motor complex in axonal transport in neurons
Neurophysiological process_Constitutive and regulated NMDA receptor trafficking
Transcription_HIF−1 targets
Pathway Maps
0
5
10
−log2FDR
PD-R272Q vs Ctrl
PD-R272Q vs iCtrl

Supplementary Figure 2 Deregulated process and pathways in midbrain organoids. (**A**) Top 25 most deregulated process (left) and pathways (right) in p.R272Q Miro1 mutant midbrain organoids (PD-R272Q) compared with healthy control (Ctrl) or isogenic control (iCtrl) (PD-R272Q vs Ctrl; PD-R272Q vs iCtrl) based on differential expression gene analysis of the single-cell RNA sequencing data. **(B**) Top 25 most deregulated process (left) and pathways (right) in PD-R272Q vs Ctrl and PD-R272Q vs iCtrl within the midbrain organoids' dopaminergic neuron clusters (dopaminergic neurons 1 and dopaminergic neurons 2).

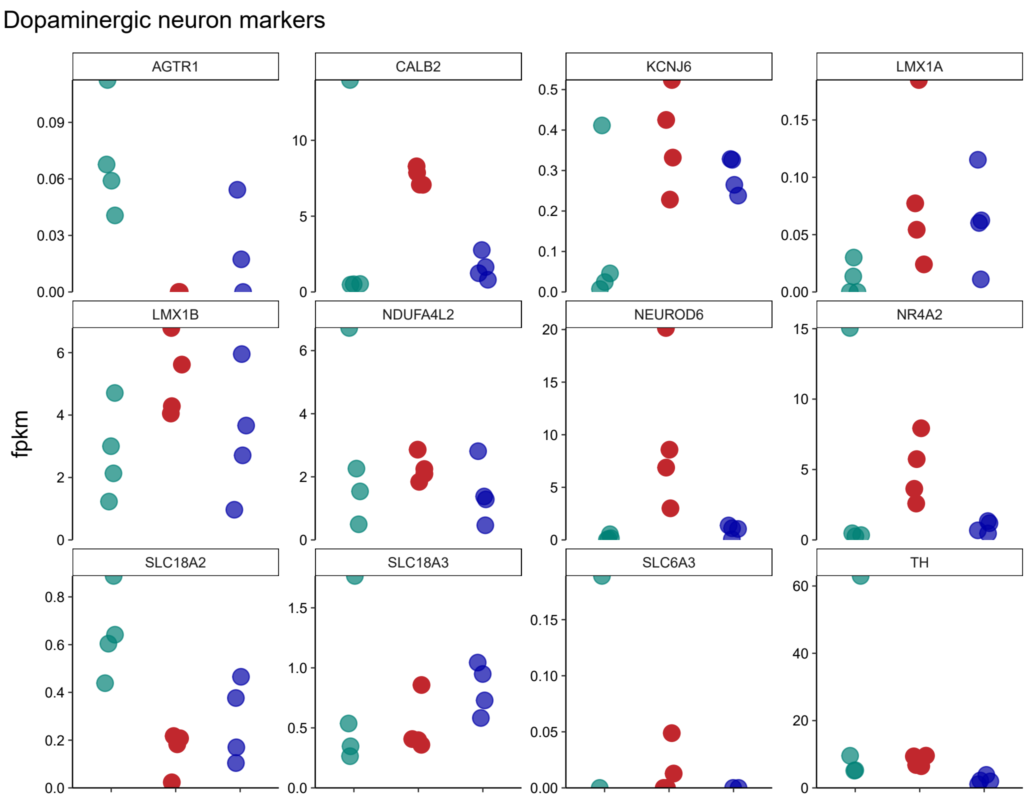
Dopaminergic neuron markers
AGTR1
CALB2
KCNJ6
LMX1A
LMX1B
NDUFA4L2
NEUROD6
NR4A2
SLC18A2
SLC18A3
SLC6A3
TH
fpkm

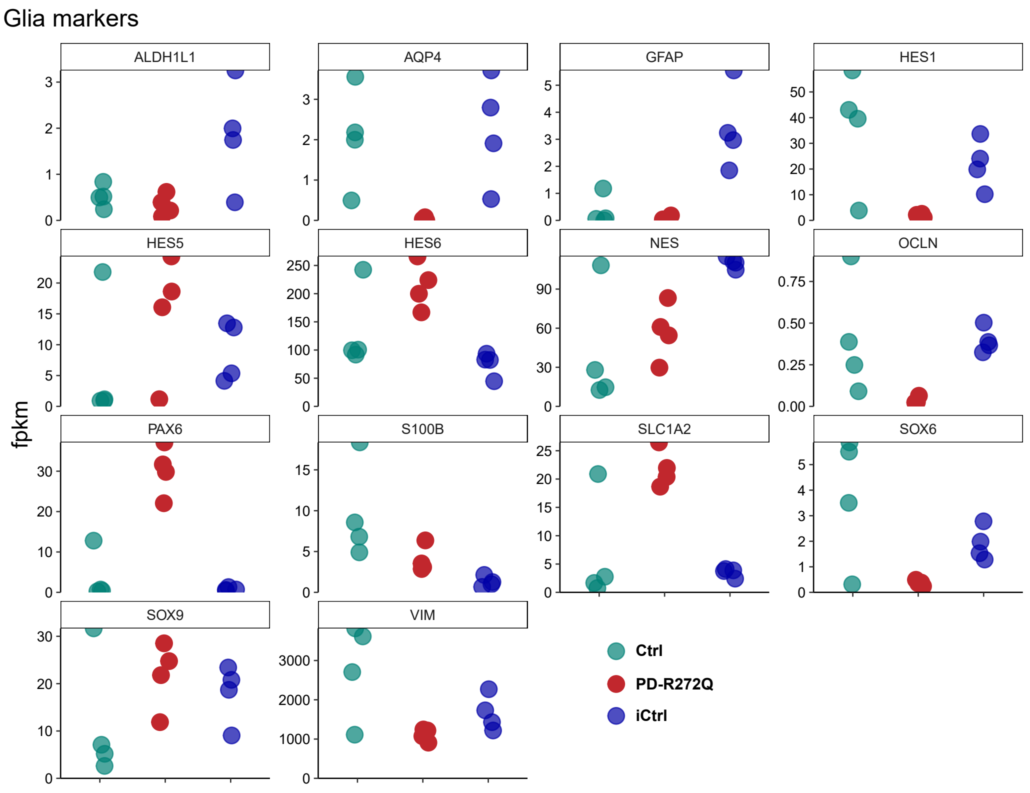
Glia markers
ALDH1L1
AQP4
GFAP
HES1
HES5
HES6
NES
OCLN
PAX6
S100B
SLC1A2
SOX6
SOX9
VIM
fpkm
Ctrl
PD-R272Q
iCtrl

Supplementary Figure 3 Dopaminergic neurons bulk RNA sequencing. Expression levels in fpkm (fragments per kilobase per million mapped fragments) of dopaminergic neuronal markers (top) and glial markers (bottom) expressed by dopaminergic neurons in the bulk RNA sequencing dataset.

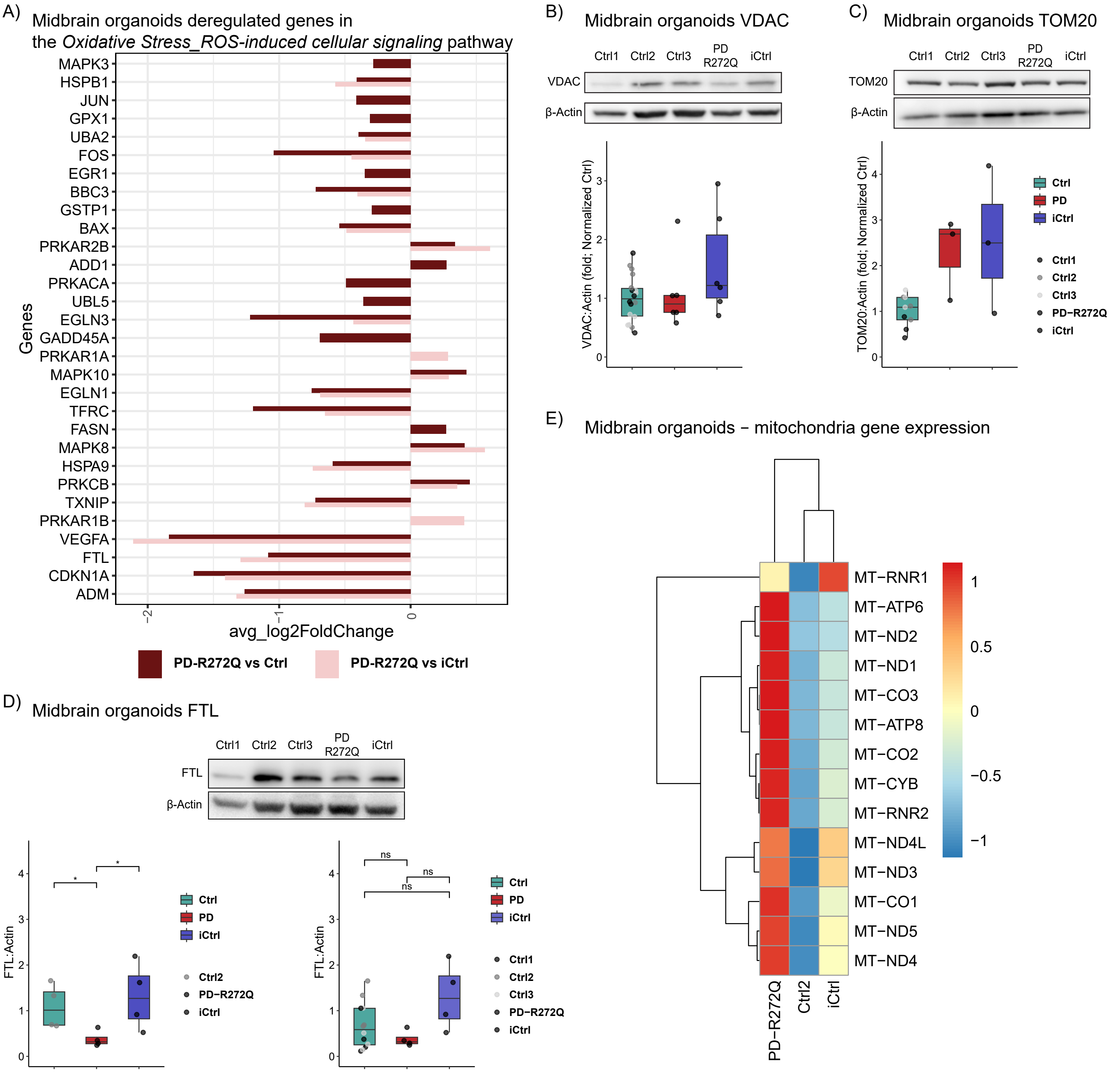
A)
Midbrain organoids deregulated genes in
the Oxidative Stress_ROS-induced cellular signaling pathway
MAPK3
HSPB1
JUN
GPX1
UBA2
FOS
EGR1
BBC3
GSTP1
BAX
PRKAR2B
ADD1
PRKACA
UBL5
EGLN3
GADD45A
PRKAR1A
MAPK10
EGLN1
TFRC
FASN
MAPK8
HSPA9
PRKCB
TXNIP
PRKAR1B
VEGFA
FTL
CDKN1A
ADM
Genes
-2
-1
0
avg_log2FoldChange
PD-R272Q vs Ctrl
PD-R272Q vs iCtrl
B)
Midbrain organoids VDAC
Ctrl1
Ctrl2
Ctrl3
PD R272Q
iCtrl
VDAC
β-Actin
VDAC:Actin (fold; Normalized Ctrl)
0
1
2
3
C)
Midbrain organoids TOM20
Ctrl1
Ctrl2
Ctrl3
PD R272Q
iCtrl
TOM20
β-Actin
TOM20:Actin (fold; Normalized Ctrl)
0
1
2
3
4
Ctrl
PD
iCtrl
Ctrl1
Ctrl2
Ctrl3
PD−R272Q
iCtrl
D)
Midbrain organoids FTL
Ctrl1
Ctrl2
Ctrl3
PD R272Q
iCtrl
FTL
β-Actin
FTL:Actin
0
1
2
3
4
*
*
Ctrl
PD
iCtrl
Ctrl2
PD−R272Q
iCtrl
FTL:Actin
ns
ns
ns
Ctrl
PD
iCtrl
Ctrl1
Ctrl2
Ctrl3
PD−R272Q
iCtrl
E)
Midbrain organoids − mitochondria gene expression
MT−RNR1
MT−ATP6
MT−ND2
MT−ND1
MT−CO3
MT−ATP8
MT−CO2
MT−CYB
MT−RNR2
MT−ND4L
MT−ND3
MT−CO1
MT−ND5
MT−ND4
PD−R272Q
Ctrl2
iCtrl
1
0.5
0
−0.5
−1

Supplementary Figure 4 Midbrain organoids oxidative stress deregulated genes and mitochondrial protein levels. (**A**) Fold change of the significant differentially expressed genes contributing to the deregulation of the *'oxidative stress ROS-induced cellular signaling'* pathway in midbrain organoids comparing p.R272Q Miro1 midbrain organoids (PD-R272Q) and healthy (Ctrl) or isogenic (iCtrl) controls (i.e. PD-R272Q vs Ctrl and PD-R272Q vs iCtrl). (**B**) Box plot showing organoids Western blotting analysis of the outer mitochondrial membrane protein VDAC (32 kDa) normalized to the housekeeping protein β-actin (42 kDa). n = 6-18 from 6 independent derivations. Representative images displayed on top. (**C**) Top: Representative images of Western blotting against the outer mitochondrial membrane protein TOM20 (18 kDa) and β-actin (42 kDa). Bottom: TOM20 quantification normalized for Ctrl condition. n = 3-9 from 3 independent derivations. (**D**) Top: Representative images of Western blotting against the ferritin light chain (FTL) protein (19 kDa) and β-actin (42 kDa). Boxplot with FTL quantification using only Ctrl2 (same as scRNAseq; left side) or all the 3 healthy controls (right side). n = 4-12 from 4 independent derivations. (**B, C, D**) Full membranes at Supplementary Western blotting data. Data are presented as median with max/min. Statistical analysis was performed using non-parametric multiple comparison Kruskal-Wallis test, $*P < 0.05$. (**E**) Heatmap showing gene expression differences between Ctrl, PD-R272Q and iCtrl in terms of expression of mitochondrial encoded genes, obtained using the single-cell RNA sequencing data.

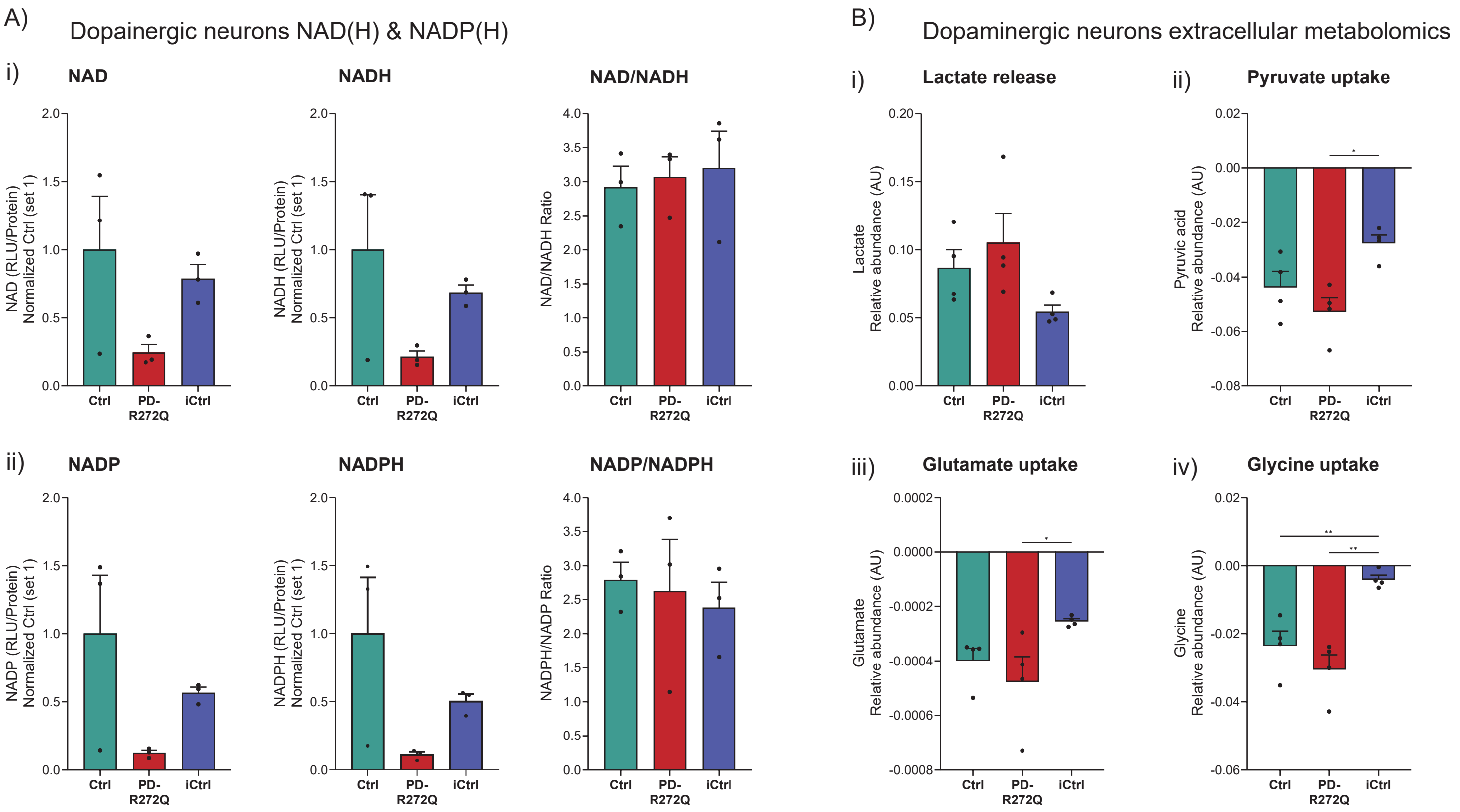

A)
Dopainergic neurons NAD(H) & NADP(H)
i)
NAD
NADH
NAD/NADH
ii)
NADP
NADPH
NADP/NADPH
NAD (RLU/Protein) Normalized Ctrl (set 1)
NADH (RLU/Protein) Normalized Ctrl (set 1)
NAD/NADH Ratio
NADP (RLU/Protein) Normalized Ctrl (set 1)
NADPH (RLU/Protein) Normalized Ctrl (set 1)
NADPH/NADP Ratio
Ctrl
PD-R272Q
iCtrl
B)
Dopaminergic neurons extracellular metabolomics
i)
Lactate release
ii)
Pyruvate uptake
iii)
Glutamate uptake
iv)
Glycine uptake
Lactate Relative abundance (AU)
Pyruvic acid Relative abundance (AU)
Glutamate Relative abundance (AU)
Glycine Relative abundance (AU)

Supplementary Figure 5 Dopaminergic neurons co-factors and metabolomic alterations. (**A**) Intracellular NAD(H) and ratio (**Ai**) as well as NADP(H) and their ratio (**Aii**) relative quantification in dopaminergic neurons. $N = 3$ independent derivations. (**B**) Relative quantification of polar metabolites in dopaminergic neuron extracellular media using gas chromatography - mass spectrometry (GC-MS). Graphs showed the release of (**i**) lactate and uptake of (**ii**) pyruvate, (**iii**) glutamate, (**iv**) glycine metabolites. $N = 4$ independent derivations. Data are presented mean ± SEM, $*P < 0.05$, $**P < 0.01$ using non-parametric multiple comparison Kruskal-Wallis test (**A**, **Biv**) or ANOVA with post-hoc Tukey HSD test (**Bi-iii**).

A) Calcium response

i) Statistics

Time (min)	Ctrl vs PD-R272Q	iCtrl vs PD-R272Q	Ctrl vs iCtrl
0 to 66	ns	ns	ns
68 to 70	*	ns	ns
72 to 230	**	ns	ns
232 to 438	**1	*2	ns
440 to 492	*	*3	ns
494 to 600	*	ns4	ns

*p < 0.05, **p < 0.01, ns non-significant;

[1] min 434 p=0.0101, min 430 p=0.0103, min 426 p=0.0104, min 422 p=0.0108;

[2] min 428 p=0.0511, min 420 p=0.0502, min 340 p=0.0501, min 296 p=0.0515, min 290 p=0.0509, min 268 p=0.0504, min 266 p=0.0529, min 262 p=0.051;

[3] min 490 p=0.0525, min 484 p=0.0516, min 482 p=0.0504, min 478 p=0.0511, min 476 p=0.051, min 470 p=0.0502, min 460 p=0.0517, min 458 p=0.056, min 446 p=0.0515;

[4] min 534 p=0.0489, min 516 p=0.0485, min 512 p=0.0487, min 504 p=0.0495, min 500 p=0.0489, min 498 p=0.0492.

ii) Area under the curve

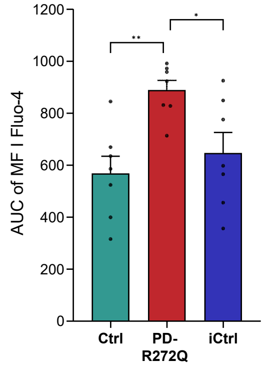

B) *SNCA* expression

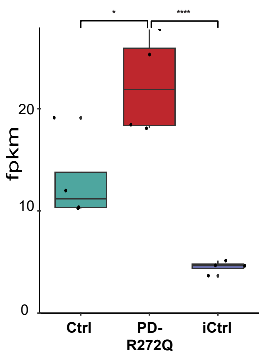

C) Total α-Synuclein (Vinculin)

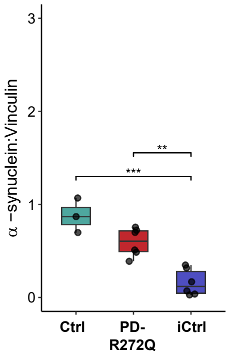

D) Calpain activity

i) Suc-LLV-AMC

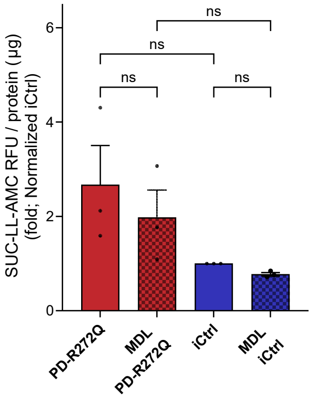

ii) Suc-LLV-AMC (no calcium)

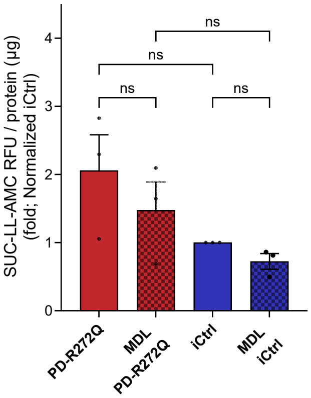

E) CDK5

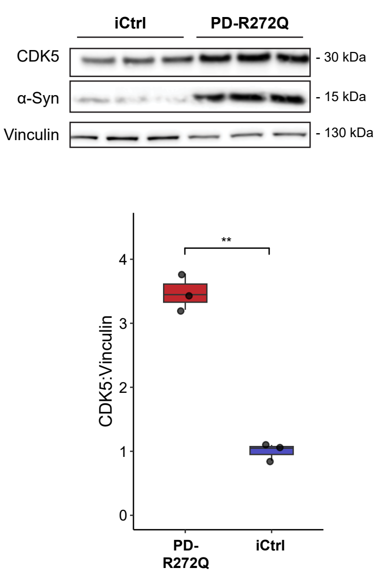

Supplementary Figure 6 p.R272Q Miro1 mutant-related alterations promoting α-synuclein accumulation *in vitro*. (**Ai**) Statistical significance values referring to **Fig. 4A right** for all timepoints analyzed in the calcium experiment done in dopaminergic neurons using two-way ANOVA. (**Aii**) Graphic depicting the calculated area under the curve (AUC) from the mean fluorescence intensity (MFI) of Fluo-4 calcium measurements in dopaminergic neurons. (**B**) *SNCA* expression levels in dopaminergic neurons obtained from bulk RNA sequencing transcriptomic data. (**C**) Quantification of Western blotting for monomeric α-synuclein normalized by vinculin. N = 3-6 independent derivations. (**E**) Calpain activity in PD-R272Q and iCtrl neurons measured using the N-succinyl-Leu-Tyr-7-amido-4-methylcoumarin (Suc-LLV-AMC) fluorescence probe treated or not with the reversible calpain inhibitor MDL-28170 (MDL) in the presence (**Ei**) or absence (**Eii**) of calcium. Data represented as relative fluorescent units per µg of protein, normalized to iCtrl. N = 3 independent derivations. (**F**) Top: Representative image of monomeric α-synuclein (α-Syn, 15 kDa), the calpain-downstream target CDK5 (30 kDa), and housekeeping protein vinculin (130 kDa) Western blotting in p.R272Q Miro1 mutant (PD-R272Q) and isogenic control (iCtrl) dopaminergic neurons. Full membranes at Supplementary Western blotting data. Bottom: Western blotting quantification of CDK5 protein. All data are represented as mean ± SEM or median with max/min, $*P < 0.05$, $**P < 0.01$, $***P < 0.001$ using ANOVA with post-hoc Tukey HSD test (**Aii-C**), two-way ANOVA (**D**) or unpaired T test (**E**).

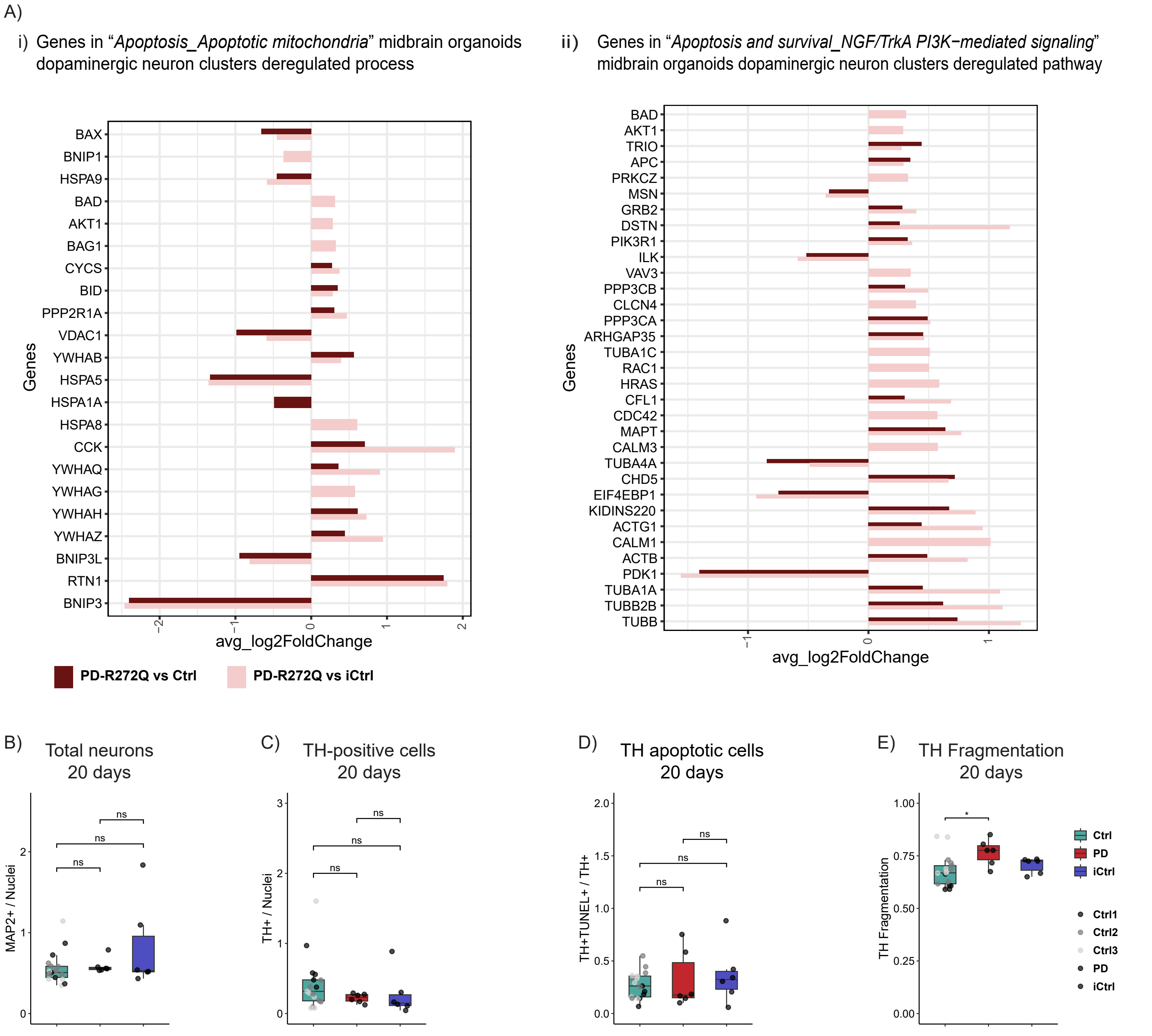
A)
i) Genes in "Apoptosis_Apoptotic mitochondria" midbrain organoids dopaminergic neuron clusters deregulated process
Genes
BAX
BNIP1
HSPA9
BAD
AKT1
BAG1
CYCS
BID
PPP2R1A
VDAC1
YWHAB
HSPA5
HSPA1A
HSPA8
CCK
YWHAQ
YWHAG
YWHAH
YWHAZ
BNIP3L
RTN1
BNIP3
-2
-1
0
1
2
avg_log2FoldChange
ii) Genes in "Apoptosis and survival_NGF/TrkA PI3K−mediated signaling" midbrain organoids dopaminergic neuron clusters deregulated pathway
Genes
BAD
AKT1
TRIO
APC
PRKCZ
MSN
GRB2
DSTN
PIK3R1
ILK
VAV3
PPP3CB
CLCN4
PPP3CA
ARHGAP35
TUBA1C
RAC1
HRAS
CFL1
CDC42
MAPT
CALM3
TUBA4A
CHD5
EIF4EBP1
KIDINS220
ACTG1
CALM1
ACTB
PDK1
TUBA1A
TUBB2B
TUBB
-1
0
1
avg_log2FoldChange
PD-R272Q vs Ctrl
PD-R272Q vs iCtrl
B)
Total neurons
20 days
MAP2+ / Nuclei
0
1
2
ns
ns
ns
C)
TH-positive cells
20 days
TH+ / Nuclei
0
1
2
3
ns
ns
ns
D)
TH apoptotic cells
20 days
TH+TUNEL+ / TH+
0.0
0.5
1.0
1.5
2.0
ns
ns
ns
E)
TH Fragmentation
20 days
TH Fragmentation
0.00
0.25
0.50
0.75
1.00
*
Ctrl
PD
iCtrl
Ctrl1
Ctrl2
Ctrl3
PD
iCtrl

Supplementary Figure 7 Midbrain organoids day 30 gene expression and day 20 immunostaining support specific loss of dopaminergic neurons within p.R272Q Miro1 mutant conditions. (**A**) Significant differentially expressed genes contributing to the deregulation of the process "*Apoptosis_Apoptotic mitochondria*" (**Ai**) and the pathway "*Apoptosis and survival_NGF/TrkA PI3K−mediated signaling*" (**Aii**) found within the dopaminergic neuron clusters (dopaminergic neurons 1 and dopaminergic neurons 2) of the midbrain organoids. Graph displays gene fold-changes comparing p.R272Q Miro1 midbrain organoids (PD-R272Q) and healthy (Ctrl) or isogenic (iCtrl) controls (i.e. PD-R272Q vs Ctrl and PD-R272Q vs iCtrl). (**B-C**) Graphic displays quantification of the total neurons (MAP2-positive signal) (**B**), and total dopaminergic neurons (TH-positive cells) (**C**) normalized for total nuclei in 20-day-old midbrain organoids. (**D**) Box plot depicts volume of TH neurons undergoing apoptosis (TH+TUNEL+) normalized by the total TH in 20-day-old organoids. (**E**) Immunofluorescent TH-based morphometric feature fragmentation index assessed in midbrain organoids at 20 days of culture. (**B-E**) n = 6-17 from 3 independent derivations, *$P < 0.05$ using non-parametric multiple comparison Kruskal-Wallis test.

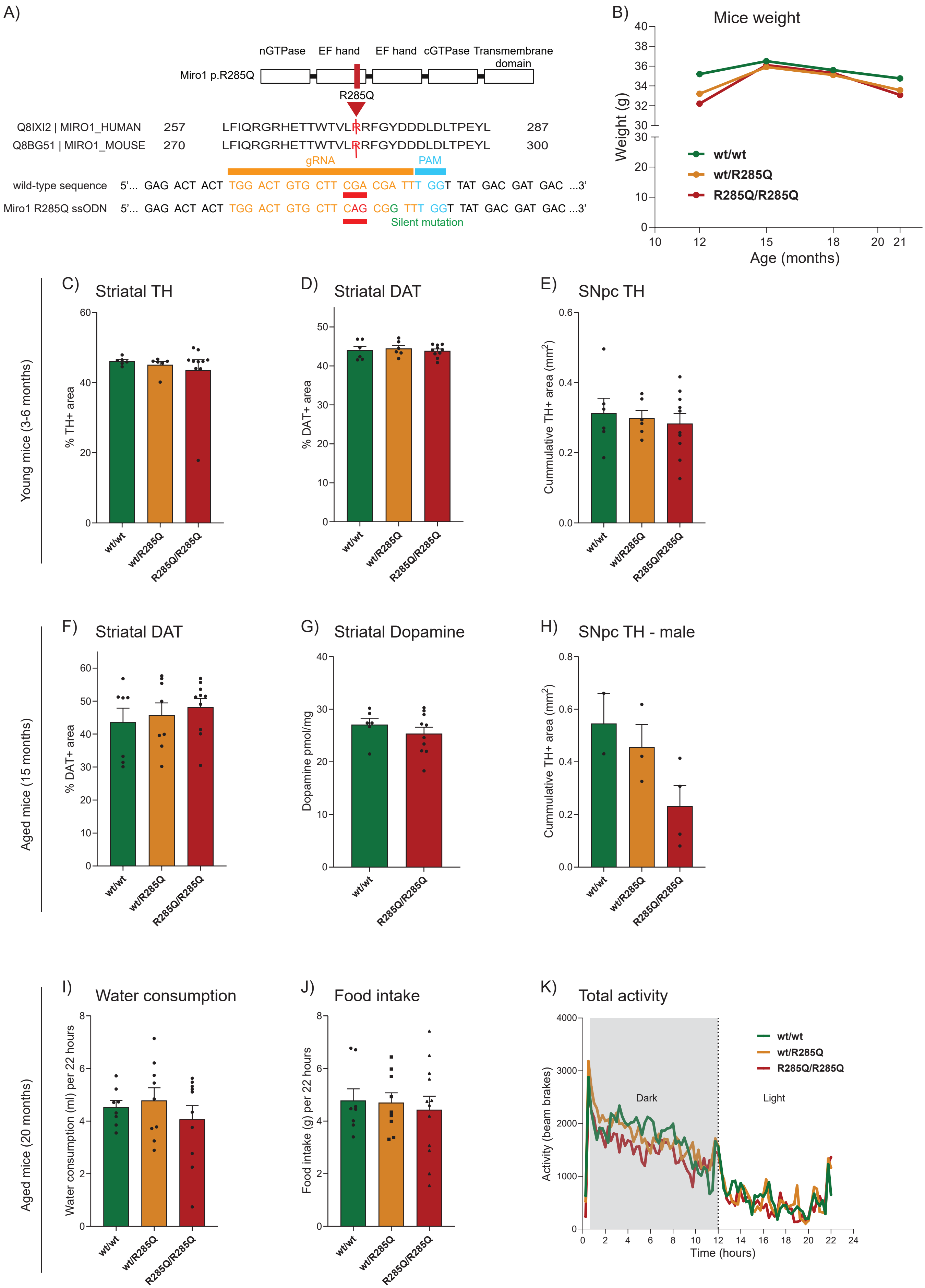
A)
nGTPase
EF hand
EF hand
cGTPase
Transmembrane domain
Miro1 p.R285Q
R285Q
Q8IXI2 | MIRO1_HUMAN 257 LFIQRGRHETTWTVLRRFGYDDDLDLTPEYL 287
Q8BG51 | MIRO1_MOUSE 270 LFIQRGRHETTWTVLRRFGYDDDLDLTPEYL 300
gRNA
PAM
wild-type sequence 5'... GAG ACT ACT TGG ACT GTG CTT CGA CGA TTT GGT TAT GAC GAT GAC ...3'
Miro1 R285Q ssODN 5'... GAG ACT ACT TGG ACT GTG CTT CAG CGG TTT GGT TAT GAC GAT GAC ...3'
Silent mutation
B)
Mice weight
Weight (g)
Age (months)
wt/wt
wt/R285Q
R285Q/R285Q
Young mice (3-6 months)
C)
Striatal TH
% TH+ area
D)
Striatal DAT
% DAT+ area
E)
SNpc TH
Cummulative TH+ area (mm²)
Aged mice (15 months)
F)
Striatal DAT
% DAT+ area
G)
Striatal Dopamine
Dopamine pmol/mg
H)
SNpc TH - male
Cummulative TH+ area (mm²)
Aged mice (20 months)
I)
Water consumption
Water consumption (ml) per 22 hours
J)
Food intake
Food intake (g) per 22 hours
K)
Total activity
Activity (beam brakes)
Time (hours)
Dark
Light
wt/wt
wt/R285Q
R285Q/R285Q

Supplementary Figure 8 Features of p.R285Q Miro1 mutant C57BL/6 mice.

(**A**) Protein amino acid sequencing and CRISPR/Cas9 guided RNA sequences used in the generation of p.R285Q Miro1 knock-in mice. (**B**) Body weight of wilde-type (wt/wt, green), p.R285Q Miro1 mutant heterozygous (wt/R285Q, orange) and homozygous (R285Q/R285Q, red) mice at months 12, 15, 18, and 21 of age. (**C, D**) Quantification of tyrosine hydroxylase (TH) (**C**) and dopamine transporter (DAT) (**D**) terminals in the striatum of 3 to 6-month-old mice. (**E**) TH cumulative area in the substantia nigra par compacta (SNpc) of 3 to 6-month-old mice. (**C-E**) wt/wt: *N* = 6F; wt/R285Q: *N* = 6F; R285/R285Q: *N* = 10F. (**F**) DAT quantification in the striatum of 15-month-old mice. (**G**) Striatal dopamine quantification (pmol/mg) in wt/wt and R285Q/R285Q 15-month-old mice using gas chromatography-mass spectrometry (GC-MS). wt/wt: *N* = 2M + 5F; wt/R285Q: *N* = 3M + 5F; R285/R285Q: *N* = 4M + 6F. (**H**) Quantification of SNpc TH-positive area in 15-month-old male mice. (**I, J**) Consumption of water (**I**) and food (**J**) for 22 hours (h) in 20-month-old mice measured with the Phenomaster Cage. (**K**) Total activity, expressed as number of beam brakes every 15 minutes, for 22h, in 20-month-old mice measured with the Phenomaster Cage. Data are presented as mean ± SEM. Statistical analysis was performed using non-parametric multiple comparison Kruskal-Wallis test (**C-J**) or two-way ANOVA (**B, K**). Abbreviation: F, female; M, male.

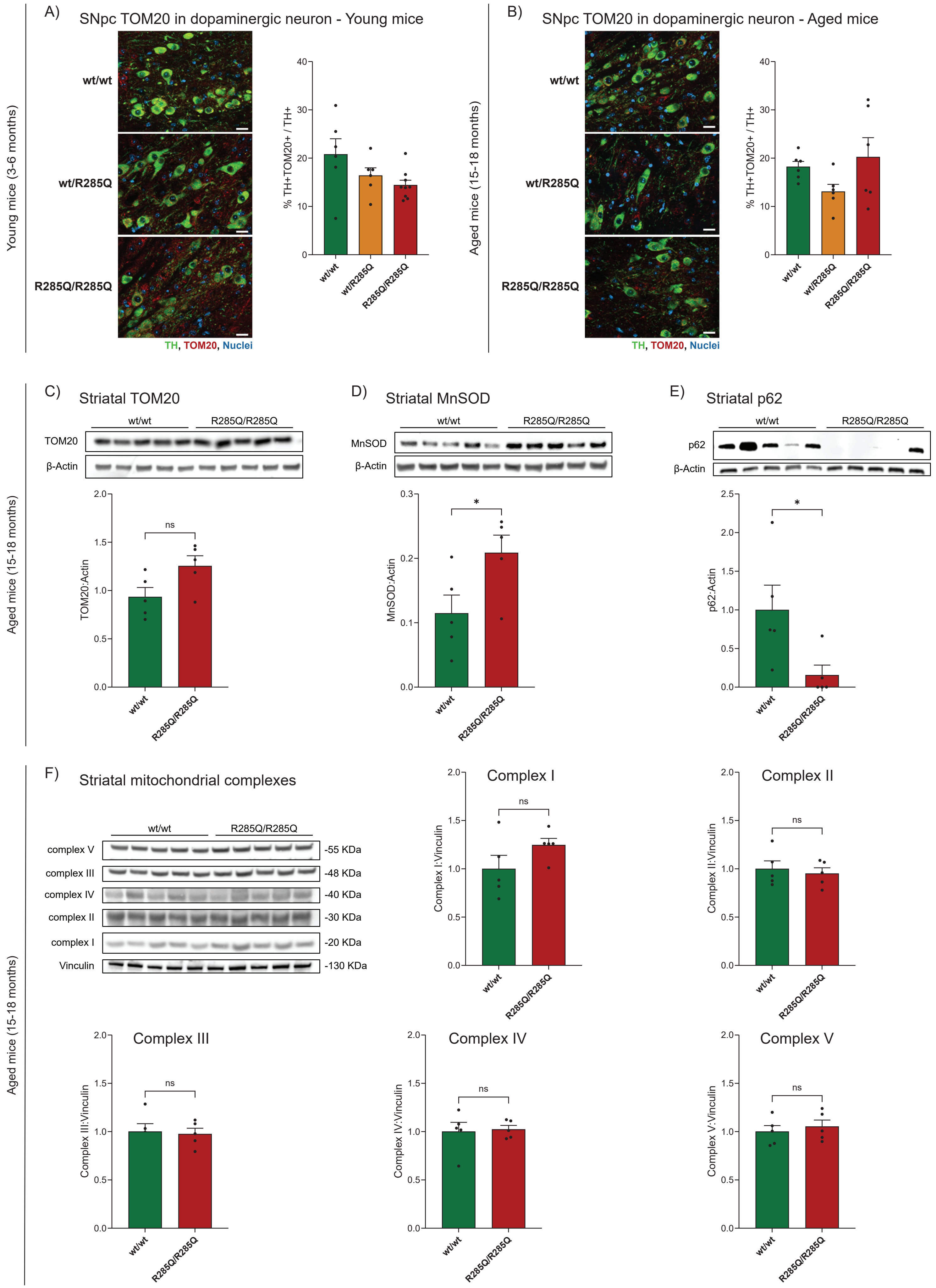
A)
SNpc TOM20 in dopaminergic neuron - Young mice
Young mice (3-6 months)
wt/wt
wt/R285Q
R285Q/R285Q
TH, TOM20, Nuclei
% TH+TOM20+ / TH+
B)
SNpc TOM20 in dopaminergic neuron - Aged mice
Aged mice (15-18 months)
C)
Striatal TOM20
TOM20
β-Actin
TOM20:Actin
ns
D)
Striatal MnSOD
MnSOD
MnSOD:Actin
*
E)
Striatal p62
p62
p62:Actin
F)
Striatal mitochondrial complexes
complex V
complex III
complex IV
complex II
complex I
Vinculin
-55 KDa
-48 KDa
-40 KDa
-30 KDa
-20 KDa
-130 KDa
Complex I
Complex I:Vinculin
Complex II
Complex II:Vinculin
Complex III
Complex III:Vinculin
Complex IV
Complex IV:Vinculin
Complex V
Complex V:Vinculin

Supplementary Figure 9 Mitochondria alterations in p.R285Q Miro1 mutant Mice.

(A, B) Representative images of TOM20, TH and DNA (DAPI) immunoreactivity in the SNpc (left) and respective quantification (right) in young (3 to 6-month-old) (**A**) and old mice (15 to 18-month-old) (**B**). Graphic displays the percentage (%) of TOM20 area occupied within the TH signal (TOM20+TH+/TH+). Young mice, wt/wt: *N* = 6F; wt/R285Q: *N* = 6F; R285/R285Q: *N* = 9F. Aged mice, wt/wt: *N* = 6F; wt/R285Q: *N* = 6F; R285/R285Q: *N* = 6F. (**C-E**) Representative Western blotting images of mitochondrial proteins (**C**) TOM20 (18 kDa) and (**D**) MnSOD (25 kDa), as well as autophagy maker (**E**) p62 (62 kDa), and housekeeping protein β-actin (42 kDa) (Top), and respective quantification (bottom). (**F**) Quantification and representative images of Western blotting against mitochondrial electron transport chain complex I (20 kDa), complex II (30 kDa), complex III (48 kDa), complex IV (40 kDa), and complex V (55 kDa) normalized for the housekeeping protein vinculin (130 kDa). (**C-F**) wt/wt: *N* = 5F; R285/R285Q: *N* = 5F aged mice. Full membranes at Supplementary Western blotting data. All data are presented as mean ± SEM. $*P < 0.05$ using ANOVA with post-hoc Tukey HSD test (**A-B**), non-parametric Mann-Whitney test (**E**) or parametric unpaired T test (**C-D, F**). Abbreviation: F, female.
