# Supplementary material for: Parkinson’s disease mutant Miro1 causes mitochondrial dysfunction and dopaminergic neuron loss
Source: Brain. 2025 Feb 6;148(10):3607–22. doi: 10.1093/brain/awaf051 (PMC12493065; doi:10.1093/brain/awaf051)
Supplement: awaf051_Supplementary_Data [file awaf051_supplementary_data.zip › brain-2024-01618-File011.pdf]

Supplementary methods

Parkinson's disease mutant Miro1 causes mitochondrial dysfunction and dopaminergic neuron loss

Axel Chemla[1], Giuseppe Arena[1], Ginevra Sacripanti[1], Kyriaki Barmpa[1], Alise Zagare[1], Pierre Garcia[1,2], Vyron Gorgogietas[1], Paul Antony[1], Jochen Ohnmacht[3], Alexandre Baron[1], Jaqueline Jung[4], Frida Lind-Holm Mogensen[3,5], Alessandro Michelucci[3], Anne-Marie Marzesco[1,§], Manuel Buttini[1,2], Thorsten Schmidt[4], Anne Grünewald[1,6], Jens C. Schwamborn[1,*], Rejko Krüger[1,3,7,*], Cláudia Saraiva[1,*]

Affiliations:

[1]Luxembourg Centre for Systems Biomedicine (LCSB), University of Luxembourg, L-4362 Esch-sur-Alzette, Luxembourg

[2]Luxembourg Center of Neuropathology (LCNP), Laboratoire National de Santé, L-3555, Dudelange, Luxembourg

[3] Luxembourg Institute of Health (LIH), L-1445 Luxembourg, Luxembourg

[4]Institute of Medical Genetics and Applied Genomics, University of Tübingen, 72076 Tübingen, Germany

[5]Faculty of Science, Technology and Medicine, University of Luxembourg, L-4365 Esch-sur-Alzette, Luxembourg

[6]Institute of Neurogenetics, University of Lübeck, 160 Lübeck, Germany

[7]Centre Hospitalier de Luxembourg, L-1210, Luxembourg, Luxembourg

[§] Current address: University Medical Center Hamburg-Eppendorf, Center for Molecular Neurobiology Hamburg, 20246 Hamburg, Germany

[*] Senior authors

Correspondence to: jens.schwamborn@uni.lu (JCS), rejko.krueger@lih.lu (RK) and claudia.saraiva@uni.lu (CS)

Supplementary methods

1. Maintenance of NESC and midbrain organoids generation

Midbrain organoids were generated following our previously described procedure.[1,2] Briefly, N2B27 medium, composed of 50:50 DMEM-F12 (Thermo Fisher Scientific 21331046) and Neurobasal (Thermo Fisher Scientific 10888022), 1% penicillin/streptomycin (Thermo Fisher Scientific 15140122), 1% GlutaMAX (Thermo Fisher Scientific 35050061), 1:100 B27 supplement without vitamin A (Life technologies 12587001) and 1:200 N2 supplement (Thermo Fisher Scientific 17502001), was used as the base culture media. NESC were cultured in Geltrex-coated (1:90 dilution in media, Thermo Fisher Scientific A1413302) 6-well plates in maintenance media (N2B27 media further supplemented with 150 µM ascorbic acid (AA, Sigma A4544), 3 µM CHIR-99021 (Axon Medchem CT99021) and 0.75 µM purmorphamine (PMA, Enzo Life Science ALX-420-045) until reaching 80% confluency. NESC cell suspension was then obtained using accutase (Sigma A6964) and cell density and viability assessed using Trypan Blue (Invitrogen T10282) in an automated cell counter (Countess II, Invitrogen). A total of 9000 cells per well, diluted in 150 µl of maintenance media, were plated in 96-well ultra-low attachment plates (faCellitate F202003). Plates were then centrifuged at 100g for 3 minutes at room temperature to promote cell contact and facilitate organoid formation. Organoids were cultured in maintenance media for 10 days with media changes every 2 days. At day 8, organoids were embedded in Geltrex droplets and kept in 24-well plates in dynamic conditions (80 rpm). From day 10 to 16, organoids were cultured in maturation media (N2B27 media further supplemented with 200 µM ascorbic acid, 500 µM Dibutyryl- cyclic adenosine monophosphate (dbcAMP, STEMCELL Technologies 100-0244), 10 ng/ml human brain-derived neurotrophic factor (hBDNF, Peprotech 450-02), 10 ng/ml glial-derived neurotrophic factor (hGDNF, Peprotech 450-10), 1 ng/ml transforming growth factor-beta 3 (TGF-β3, Peprotech 100-36E) in the presence of 1 µM purmorphamine, with media changes performed every 2 days. From day 16 onward, organoids were cultured in maturation media with media changes occurring every 3 to 4 days. Embedded organoids were mostly used. However, due to size limitations, non-embedded organoids were used for assays such as Seahorse. Organoids were kept in maintenance media for 2 days, then cultured for 6 days in maturation media containing 1 µM purmorphamine, and finally kept in maturation media until the assay day. All measures were done around day 30 of organoid culture, except for the experiments in

Supplementary Fig. 7B-E, where 20-day-old organoids were used. Cultures were tested monthly for mycoplasma contamination using the LookOut Mycoplasma PCR Detection Kit (Sigma MP0035-1KT).

2. Single cell RNA sequencing (scRNAseq) in midbrain organoids

a. Midbrain organoids dissociation and single-cell isolation

30-day-old midbrain organoids from one healthy control (Ctrl2), the PD patient carrying the p.R272Q Miro1 mutation (PD-R272Q), and the respective gene-corrected line (iCtrl) were used for scRNAseq analysis (GEO: GSE237133). A total of 30 embedded organoids per condition were pooled for the analysis. Midbrain organoids were collected from their culture medium and washed with 1x PBS (phosphate-buffered saline, Gibco 10010-23). Organoids were digested in 1 ml sCelLive™ Tissue Dissociation Solution (Singleron Biotechnologies 1190062) in a 15 ml conical tube (Sarstedt 62.5544.003) and placed in a thermal shaker at 50 rpm at 37°C for 30 minutes. The state of dissociation was checked at regular intervals under a light microscope. Following digestion, the suspension was filtered using a 40 µm sterile strainer (Greiner 542040). Cells were then centrifuged at 350g for 5 minutes at 4 °C, and the pellets were resuspended in 500 µl PBS. Cells were stained with Acridine Orange/Propidium Iodide Stain (Logos Biosystems F23001), and the cell number and viability were calculated using LUNA-FX7™ Automated Cell Counter (Logos Biosystems).

b. scRNAseq library preparation

The scRNAseq libraries were constructed using GEXSCOPE™ Single Cell RNAseq Library Kit (Singleron Biotechnologies 4161031) according to the manufacturer´s instructions. Briefly, for each library, the concentration of the single-cell suspension was adjusted to $3x10^5$ cells/ml with PBS, and the suspension was loaded onto an SD microfluidic chip to capture 6000 cells. Paramagnetic beads conjugated to oligodT probes carrying a unique molecular identifier (UMI) and a barcode unique to each bead (from the same kit) were loaded, followed by cell lysis. The beads bound to polyadenylated mRNA were extracted from the chip and reverse transcribed into cDNA at 42 °C for 1.5 hours, followed by cDNA amplification by PCR. The cDNA was then fragmented and ligated to indexed Illumina adapters. The fragment size distribution of the final

amplified library was obtained using the Agilent Fragment Analyzer.

c. Library sequencing

The library concentration was calculated using the Qubit 4.0 fluorometer and the libraries were pooled in an equimolar fashion. The single cell libraries were sequenced on the Illumina NovaSeq 6000 using a 2x150-bp (base pair) approach to a final depth of 90 GB per library. The reads were demultiplexed according to the multiplexing index sequencing on Illumina's BaseCloud platform.

d. Transcriptome data pre-processing

Pre-processing of Fastq data was conducted using CeleScope® (v.1.3.0; www.github.com/singleron-RD/CeleScope; Singleron Biotechnologies GmbH, RRID SCR_023553) to generate raw data, using default parameters. Low-quality reads were removed. Sequences were mapped using STAR (https://github.com/alexdobin/STAR), and the human reference GRCh38 and genes were annotated using Ensembl 92. The reads were assigned to genes using featureCount (https://subread.sourceforge.net/; RRID SCR_009803) and the cell calling was performed by fitting a negative bimodal distribution and by determining the threshold between empty wells and cell-associated wells, to generate a count matrix file containing the number of Unique Molecular Identifier (UMI) for each gene within each cell.

e. Transcriptome data processing and plotting

scRNAseq data was analyzed using Seurat R toolkit for single cell genomics version 4.2.0[3] on R version 4.2.2. Cells with unique feature counts lower than 300 and higher than 7000 were removed as low-quality, empty droplets or probable doublets, respectively. Cells with mitochondrial genes higher than 10 or 15% were filtered out as an additional measure for low-quality cells. Datasets were merged, log-normalized, and integrated based on the 20 principal component analysis (PCA) components using Seurat integration workflow for better identification of shared cellular populations across the datasets.[4] After integration, merged data were scaled to reduce the variance in gene expression across cells. Seven distinct cell populations were identified by applying Louvain algorithm modularity optimization with a resolution of 0.15, based on the top 20 principal components. Visualization was done based on their transcriptomic similarities according to the

uniform manifold approximation and projection (UMAP) technique.[5] Cell identities were determined using the online tool GeneAnalytics,[6] where marker genes of each cell cluster (identified using the *FindAllMarkers* function of Seurat) were given in the tool for cell type identification choosing *in vitro* parameter for brain cells. The identity of each cluster was further validated by the expression pattern of known cell type-specific markers. Differentially expressed genes (DEG) were detected using the *FindMarkers* function of Seurat, comparing the PD-R272Q midbrain (ident.1) against the control (Ctrl) midbrain (ident.2) or PD-R272Q midbrain (ident.1) against the isogenic control (iCtrl) midbrain (ident.2) in the whole dataset and for each cellular population separately. Significantly differentially expressed genes (p.adjust < 0.05 and logfc threshold = 0.25) were selected for further enrichment analysis using MetaCore (version 2022 Clarivate; RRID SCR_008125), and Rstudio (R version 4.2.2) used to visualize genes fold change from the most relevant enriched pathways.

3. Midbrain organoids flow cytometry

Five to ten non-embedded 30-day-old organoids, per cell line, per replicate, were pooled and dissociated into single cells with accutase for 1 hour at 37°C under dynamic conditions, followed by mechanical dissociation. Cell suspension was centrifuged at 400g for 5 minutes and washed once with assay media or PBS. For reactive oxygen species (ROS) measurement, samples were stained with 1 nM MitoSOX (Invitrogen M36008) in phenol-red-free DMEM-F12 in combination with Zombie NIR (1:10,000 dilution; Biolegend 423106) and incubated for 25 minutes. For mitochondrial membrane potential (MMP) assessment, cells were incubated for 30 minutes in N2B27 media containing 1 nM tetramethylrhodamine (TMRM; Invitrogen I34361), 100 nM MitoTracker Green (Invitrogen M7514), and 1:10,000 Zombie NIR. Cells were then rinsed twice in PBS followed by centrifugation at 400g for 5 minutes. A BD LSRFortessa (BD Biosciences) running the BD FACSDiva™ Software (BD Biosciences, RRID SCR_001456) was used to record 10,000 single-cell events per sample. Event counts and intensity were analyzed with FlowJo software (v.10.8.1; RRID SCR_008520).

4. Midbrain organoids Mito stress test (Seahorse XF)

Seahorse XF Cell Mito Stress Test (Agilent) was performed in 35-day-old non-embedded

organoids using Seahorse XFe96 Spheroid FluxPak (Agilent 102905-100) according to the manufacturer's instructions. Organoids were seeded on spheroids microplates previously coated with Corning Cell-Tak Cell and Tissue Adhesive (1:60 dilution in 0.1 M sodium bicarbonate; Corning 354240) for 1 hour at 37°C in Seahorse XF-DMEM medium, pH 7.4 (Agilent 103575-100), further supplemented with 1 mM L-glutamine (Gibco 25030-024), 1 mM pyruvate (Thermo Fisher Scientific 11360070), and 21.25 mM glucose (Sigma G7021-100g), in a non-CO_2 incubator. Oxygen consumption rates (OCR) were measured over time in the Seahorse XFe96 analyzer under basal conditions and after injection of different drugs: 5 µM oligomycin (Sigma 75351-5mg); 1 µM FCCP (Abcam ab120081); and 1 µM antimycin A (Abcam ab141904) with 1 µM rotenone (Sigma R8875-1G). Data analysis was done using Seahorse Wave Desktop software (Agilent, RRID SCR_014526). OCR results were normalized by the respective area of each organoid, obtained using a Cytation5M reader (BioTek). Datapoints that did not respond to drugs or presented abnormal OCR values were excluded. The mean value of at least three organoids per cell line from each batch was used.

5. Midbrain organoids metabolomics

Polar intracellular metabolites were analyzed in 30-day-old organoids. Five embedded organoids per line were pooled into Precellys tubes, washed 3 times in miliQ sterile water, snap frozen, and kept at -80 °C until processed. A total of five independent samples coming from five pooled organoids from two independent organoid derivations (batches) were analyzed. Metabolite extraction and relative quantification were done in a blind way.

a. Metabolite extractions

Metabolite extraction of batch 1 and batch 2 were done separately. Snap-frozen organoids were transferred to 2 ml Precellys tubes and 1600 µl (batch 1) or 1500 µl (batch 2) of cold extraction fluid was used. Precellys tubes were prefilled with 600 mg of ceramic beads (1.4 mm, Qiagen 1103955) to facilitate metabolite extraction. Pre-cooled (4°C) extraction fluid was composed of 4:1 ratio of methanol (Carl Roth AE71.1; Rotisolv, purity ≥ 99.95%) and Mill-Q water (in-house, Milli-Q Advantage A10, 18.2 MΩ•cm, <3 ppb TOC) with 0.8 µg/ml internal standards: internal standards, [$_{13}C^{10}$,$_{15}N^{5}$] AMP sodium (Sigma-Aldrich 650676), 6-chloropurine riboside (Sigma-

Aldrich 852481), 2-Chloroquinoline-3-carboxylic acid (Sigma-Aldrich 688517), 4-Chloro-DL-phenylalanine salt (Sigma-Aldrich C6506), Nε-Trifluoroacetyl-L-lysine (Sigma-Aldrich 53604), sucralose (Sigma-Aldrich 69293) and $^{13}C_3$-caffeine-trimethyl (Eurisotop CLM-514-1).

Precellys tubes were homogenized with a 30 second cycle at 6000 rpm (0 to 5 °C). For batch 1, the total volume was divided into 2 Precellys tubes followed by the addition of 800 µl of extraction fluid in each followed by a second round of homogenization. A total of 1800 µl of solution from both tubes (batch 1) was transferred to a 2 ml Eppendorf and vortexed (Eppendorf Thermomixer C) for 15 minutes at 2000 rpm (4 °C). For batch 2, after homogenization, 1400 µl of solution was transferred to a 1.5 ml Eppendorf. In both cases, Eppendorf tubes were centrifuged at 21,000g for 10 minutes at 4 °C. After, either two times 500 µl (batch 1) or 800 µl (batch 2) supernatant were transferred to new 1.5 mL Eppendorf tubes, which were evaporated overnight at -4 °C on a refrigerated centrifugal vacuum concentrator (CentriVap 7310000, Labconco). The dried metabolite pellets were stored at -80 °C until LC-MS analysis.

b. Hydrophilic Interaction Liquid Chromatography- Mass Spectrometry (HILIC-MS) measurements

Targeted HILIC-MS measurement was done using a Themo UHPLC Vanquish UHPLC equipped with a binary pump and coupled to a Thermo Exploris 240 mass-spectrometer. Dried metabolite pellets were reconstituted in 50% ACN and filtered using PHENEX-RC 4 mm syringe filter (Phenomenex AF0-3203-52). Metabolites were separated using a Hydrophilic Interaction Liquid Chromatography (HILIC) column (SeQuant ZIC pHILIC, 5 µm particles, 2.1 x 150 mm) protected with a guard column (SeQuant ZIC-pHILIC Guard 20 x 2.1 mm). Detailed LC-MS settings are provided below (section 5.b.i and Supplementary Table I). Peak areas were integrated and exported to Microsoft Excel via the Thermo TraceFinder software (version 5.1; RRID SCR_023045). Metabolite identification confidence was level 1 with all metabolites being verified by an in-house library of standards. Raw values were exported using the sum of all peak areas within a sample. Normalized data was further analyzed using RStudio software (Version 4.3.0; RRID SCR_000432) and further normalized between the different cell lines, to account for cell number differences between conditions, using the Perform Probabilistic Quotient Normalization (PQN).

i. Detailed LC-MS setting

Analytical column: SeQuant® ZIC-pHILIC 5µm polymer 150 x 2.1 mm

Guard column: SeQuant® ZIC-pHILIC Guard 20 x 2.1 mm

Mobile phase A: 20 mmol/L ammonium acetate in H2O (pH 9.2, + 5 µM MA)

Mobile phase B: ACN (pH unadjusted, 5 µM MA)

Supplementary Table 1 Gradient parameters used in midbrain organoid LC-MS.

Time (minutes)	% B	Flow rate (µl/minute)
0.00	80	200
3.00	80	200
18.00	20	200
19.00	80	200
24.5	80	200
25.5	80	400
29.5	80	400
30.00	80	200

Flow rate: 0.20-0.40 ml/min; *Column temp.:* 45 °C & 4 °C; *Injection volume:* 5 µl; *Autosampler temp.:* 4 °C.

MS parameters: Instrument: Exploris 240; Ion mode: Polarity Switching (positive/negative ESI in Full scan).

ESI Source: Sheath gas flow rate: 35; Aux gas flow rate: 7; Sweep gas flow rate: 0; Spray voltage kV: 3.5; Capillary temp.: 400 °C; S-lens RF level: 70.0; Aux gas heater temp. (°C): 275.

Scan parameters Full MS: MS1 Scan range: 75-1000 m/z; Polarity: POS/NEG; Spray voltage of 3 kV in both positive and negative mode; MS1 Resolution: 60,000; MS1 AGC target: “standard”; MS1 Maximum injection time: 100 ms; MS2 Resolution: 30,000; MS2 AGC target: “standard”; MS2 Maximum injection time: “auto”; MS2 isolation window: 0.4 m/z; Collision energy: 30 V; Loop count: 3; Dynamic exclusion time: 3 s with exclusion after 1 acquisition.

6. Bulk RNA sequencing (RNAseq) in dopaminergic neurons

30-day-old dopaminergic neurons from four batches, with three technical replicates each, were used. RNA extraction was performed using the RNeasy Kit (Qiagen 74104) according to the manufacturer’s instructions using 600 µl RLT lysis buffer containing 1:1,000 2-mercaptoethanol (Sigma Aldrich M3148). After RNA extraction, eluted RNA concentration was assessed using a

NanoDrop™ spectrophotometer. RNA quality was further assessed using the Agilent 2100 Bioanalyzer showing RNA integrity (RIN) values > 8.

RNA library preparation was performed using TruSeq Stranded mRNA library prep kit (Illumina 20020594) according to the manufacturer's protocol and then sequenced using NextSeq2000 (Illumina) at the LCSB Genomics platform (RRID SCR_021931). For all samples paired end reads of 51 bp length were generated.

Data (GEO: GSE238129) was processed using an in-house snakemake workflow (https://git-r3lab.uni.lu/aurelien.ginolhac/snakemake-rna-seq; release v0.2.3 and singularity image v0.4). Snakemake pipeline is under license https://git-r3lab.uni.lu/aurelien.ginolhac/snakemake-rna-seq/-/blob/main/LICENSE. Raw read quality was assessed by FastQC (v0.11.9; RRID SCR_014583).[7] Adapters were removed using AdapterRemoval (v2.3.2; RRID:SCR_011834),[8] with a minimum length of the remaining reads set to 35 bp. Reads were mapped to hg38 (GRCh38.p13) using STAR (v.2.7.9a),[9] and reads were counted using featureCounts from the R package Rsubread (v2.8.1; RRID SCR_016945).[10] All transcripts with counts > 10 were used for differential gene expression analysis using the R package DESeq2 (v1.34.0; RRID SCR_015687).[11] apeglm was used for normalization in DEseq2 (v.1.16.0).[12] FPKM (fragments per kilobase of exon per million mapped fragments) were calculated using DESeq2 package. Pathway analysis on DEG with minimum FPKM of > 1, false discovery rate < 0.05, and a minimum log2-fold change cut-off of +/- 1.5 was performed using Ingenuity Pathway Analysis tool (Qiagen, version: 60467501) and the EnrichR online tool (https://maayanlab.cloud/Enrichr; RRID SCR_001575).

7. Dopaminergic neurons imaging

iPSC-derived dopaminergic neurons were re-plated on day 15 of differentiation at a density of 100,000 cells per well into PerkinElmer Phenoplate 96-well plates (PerkinElmer 6055300) and kept in maturation media until imaged (day of differentiation 30, approximately). Yokogawa CV8000 standalone high-content screening microscope using a 60x (intracellular ROS and calcium imaging) or 20x (mitochondrial membrane potential) objective was used for dopaminergic neurons live-cell imaging experiments. To maintain cell integrity during image acquisition, neurons were kept under controlled conditions: 5 % CO_2, 37 °C temperature, and 80% humidity. A total of 17 fields for intracellular ROS, 1 field for calcium imaging, and 21 fields in the x, y, z

axis were acquired per well. MATLAB scripts (v.2021a) developed in-house were further used for image analysis.

a. Intracellular ROS (CellROX Deep Red & CellTracker Green)

The cell-permeant CellROX Deep Red dye (Thermo Fisher Scientific C10422), which allows for live assessment of intracellular ROS, was used in combination with the general cellular marker CellTracker Green (Thermo Fisher Scientific C7025), and nuclei dye Hoechst-33342 (Invitrogen 21492) to evaluate intracellular ROS using live imaging. Two days before the assay cells were changed to maturation media without antioxidants (without B27 and ascorbic acid). On the day of the assay, dopaminergic neurons were incubated for 30 minutes with 10 µM CellROX Deep Red, 0.5 µM CellTracker Green, and 1 µg/ml Hoechst-33342 at 37 °C with 5% CO_2, washed once with PBS and kept in maturation media during imaging acquisition.

b. Mitochondrial membrane potential (TMRE & Mitotracker Green)

Dopaminergic neurons were stained with the specific mitochondria membrane potential maker TMRE (Invitrogen T669) at a concentration of 20 µM, combined with 0.1 nM MitoTracker Green (Invitrogen M7514) and 1 µg/ml Hoechst-33342 in N2B27 media for 30 minutes in a cell culture incubator (37 °C with 5% CO_2). Cells were washed once in PBS and further re-incubated in N2B27 culture media with 20 µM TMRE followed by live imaging.

c. Calcium imaging

For calcium imaging, dopaminergic neurons were incubated for 1 hour with 50% maturation media 50% of 2X calcium indicator Fluo4-Direct (Thermo Fisher Scientific F10471) following the manufacturer's instructions, and Hoechst-33342 (1 µg/ml). Imaging was done for a total of 10 minutes at 0.5 Hz. For the first minute, imaging was done at basal conditions to establish baseline calcium levels (F0). At minute 1, dopaminergic neurons were exposed to the calcium ionophore ionomycin (10 µM; Sigma-Aldrich I0634-1MG), dispensed in an automated way by Yokogawa CV8000, allowing to understand dopaminergic neurons response to calcium influx increase (F1).

8. Dopaminergic neurons Mito stress test (Seahorse XF)

OCR and pH were measured in whole dopaminergic neurons using the Seahorse XFe96 Cell Metabolism Analyzer and Seahorse FluxPak (Agilent 103775-100), following the manufacturer's instructions on the mito stress test. A total of 100,000 cells/well were seeded into Geltrex-coated Seahorse XFe96 well plates 24 hours prior to the assay. On the assay day (30 days of culture), neurons were incubated with 175 µl/well of Seahorse base media supplemented with 382 µg/ml D-glucose (Sigma D8375), 2 mM L-glutamine (Gibco 35050061) and 40 µg/ml sodium pyruvate (Sigma P5280) for 1 hour at 37°C in a non-CO_2 incubator. OCR were measured over time under basal conditions and upon injection of 1 µM oligomycin, 1 µM FCCP (Sigma C2920), and 0.5 µM antimycin A (Sigma A8674) with 0.5 µM rotenone. Normalization was done using DNA quantification with the CyQUANT® assay (Invitrogen C7026). Seahorse Wave Desktop software was used for data analysis.

9. Dopaminergic neurons NAD(P)/NAD(P)H and ATP measurement

At day of differentiation 30, dopaminergic neurons were harvested with accutase for measuring ATP (40,000 cells per replicate) and NAD(P)/NAD(P)H (25,000 cells per replicate). Measurements were done using commercially available kits from Promega, following the manufacturer's protocol: CellTiter-Glo (Promega G7570), NAD/NADH-Glo (Promega G9071) and NADP/NADPH-Glo (Promega G9081). Luminescence was read using the Cytation5M reader. All values were normalized to protein amount quantified with the Pierce™ BCA Protein Assay Kit according to the manufacturer's instructions. For all assays, a total of 4 independent batches (derivations) with 2 technical replicates per batch were performed.

10. Extracellular metabolomics on dopaminergic neurons

a. Sample collection and metabolite extraction

At day 30 of differentiation, maturation media that has been in contact with dopaminergic neurons for the previous 48h was collected, filtered using Phenex Regenerated Cellulose (RC) syringe filters to remove any cells or debris, and frozen at -80 °C until extraction and gas chromatography (GC)-MS measurement. Metabolite derivatization was performed by using a multi-purpose sample preparation robot (Gerstel). Dried medium extracts were dissolved in 30 µl pyridine, containing

20 mg/ml methoxyamine hydrochloride (Sigma-Aldrich 89803), for 120 minutes at 45 °C under shaking. After adding 30 µl of N-methyl-N-trimethylsilyl-trifluoroacetamide (Macherey-Nagel 701270.510) samples were further incubated for 30 minutes at 45 °C under continuous shaking followed by GC-MS analysis.

b. Gas chromatography – Mass spectrometry (GC-MS)

GC-MS analysis was performed on an Agilent 8890 GC coupled to an Agilent 5977B MS (Agilent Technologies). A sample volume of 1 µl was injected into a Split/Splitless inlet, operating in split mode (20:1) at 270 °C. The gas chromatograph was equipped with a 30 m (I.D. 0.25 mm, film 0.25 µm) ZB-5MSplus capillary column (Phenomenex 7HG-G030-11-GGA) with 5 m guard column in front of the analytical column. Helium was used as carrier gas with a constant flow rate of 1.4 ml/minute. The GC oven temperature was held at 90 °C for 1 minute and increased to 220 °C at 10 °C/minute. Then, the temperature was increased to 300 °C at 20 °C/minute followed by 4 minutes post run time at 325 °C. The total run time was 22 minutes. The transfer line temperature was set to 280 °C. The MSD was operating under electron ionization at 70 eV. The MS source was held at 230 °C and the quadrupole at 150 °C. Mass spectra were acquired in selected ion monitoring (SIM) mode for precise quantification of medium components. Supplementary Table II shows the masses used for quantification and qualification of the derivatized target analytes (dwell times between 20 and 70 ms).

c. Data processing and normalization

All GC-MS chromatograms were processed using MetaboliteDetector (v3.2.20190704).[13] Compounds were annotated by retention time and mass spectrum using an in-house mass spectral (SIM) library (overall similarity > 0.80). The following deconvolution settings were applied: peak threshold: 2; minimum peak height: 2; bins per scan: 10; deconvolution width: 8 scans; no baseline adjustment; minimum 1 peak per spectrum; no minimum required base peak intensity. The internal standards (U-13C5-ribitol and pentanedioic-d6 acid) were added at the same concentration to every sample to correct for uncontrolled sample losses, and analyte degradation during metabolite extraction and sensitivity drifts during measurements. The dataset was normalized by using the response ratio of the integrated peak area of the analyte and the integrated peak area of the internal standard. Further normalization based on the total cell number that the media was in contact with

was performed and the relative abundance of metabolites was plotted in GraphPad Prism v10 (RRID SCR_002798) using negative values for metabolites consumed and positive values for metabolites released by the dopaminergic neurons.

Supplementary Table II Extracellular metabolomics on dopaminergic neurons. Masses used for quantification and qualification of the derivatized target analytes (dwell times between 20 and 70 ms).

Analyte Name	Quantification Ions (m/z)	Qualification Ion I (m/z)	Qualification Ion II (m/z)
**Pyruvic acid 1MEOX 1TMS**	174-179	158.1	189.1
**Lactic acid 2TMS**	219.1-224.1	190.1	117.1
**Alanine 2TMS**	218.1-223.1	190.1	116.1
**Valine 2TMS**	144.1	218.1	246.2
**Urea 2TMS**	189.1	103.1	171.1
**Leucine 2TMS**	158.1	218.1	232.2
**Isoleucine 2TMS**	158.1	218.1	232.2
**Glycine 3TMS**	276.1-280.1	248.1	174
**Serine 3TMS**	306.1-311.1	218.1	204.1
**Threonine 3TMS**	218.1	291.2	320.2
**IS Pentanedioic acid-D6 2TMS**	267.1	163.1	239.1
**Methionine 2TMS**	176.1	250.1	293.1
**Glutamic acid 3TMS**	363.2-371.2	246.1	348.2
**Phenylalanine 2TMS**	192.1	218.1	266.1
**Asparagine 3TMS**	231.1-236.1	132.1	348.1
**IS [UL-13C5]-Ribitol 5TMS**	220.1	310.2	323.2
**Glutamine 3TMS**	347.2-354.2	245.1	156.1
**Fructose 1MeOX 5TMS**	307.2, 310.2	217.1, 220.1	
**Glucose 1MEOX 5TMS**	319.2, 323.2	217.1, 220.1	
**Lysine 4TMS**	174.1	317.2	434.3
**Tyrosine 3TMS**	218.1	179.1	280.2
**Inositol 6TMS**	305.1	318.1	507.3
**Tryptophan 2TMS**	218.1-223.1	130	348.2
**Alanyl-glutamine 3TMS**	418.2	347.2	216.1
**Cystine 4TMS**	218.1-223.1	297.1	411.2

11. *In vivo* - Mice

All mouse experiments were performed according to the European FELASA guidelines for animal experimentation (see ethical approval at *Materials and methods* Mice section). Mice weight was assessed throughout the study at months 12, 15, 18, and 21.

a. Housing

Mice were housed and bred in dedicated facilities under specific pathogen-free (SPF) conditions, then transferred to a conventional mouse facility at least one week before any manipulation or measurement. Both facilities had 12 hours of alternating dark/light cycles. Mice always had *ad libitum* access to standard mouse food (Sniff V 1534-300) and water. Health monitoring was performed quarterly and yearly, according to the FELASA 2014 pathogen list.

b. Generation of p.R285Q Miro1 knock-in mice

p.R285Q Miro1 knock-in mice (which represent the human p.R272Q Miro1 ortholog mutation) were generated by CRISPR/Cas9-mediated gene editing in mouse zygotes. Briefly, pronuclear stage zygotes were harvested from C57BL/6N mice super ovulated C57BL/6N females mated with C57BL/6N males. The pronucleus of embryos were microinjected with a mix containing 25 ng/µl Cas9 mRNA, 60 ng/µl Recombinant S. pyogenes Cas9 nuclease, 0.6 pmol/µl Alt-R® CRISPR-Cas9 crRNA (protospacer TGGACTGTGCTTCGACGATT; IDT, Inc.): 0.6 pmol/µl Alt-R® CRISPR-Cas9 tracrRNA duplex (IDT, Inc.) and 50 ng/µl of specific mutagenic ssODN Miro1 R285Q (5' - GGTTTTCTCTTTTTACATACACTTTTTATCCAGAGGGGGAGGCATGAGACTACTTGGACTGTGCTTCAGCGGTTTGGTTATGACGATGACCTGGACCTGACGCCTGAGTATTTATTCCCCCTGTATGTACCTCAGCGCTC - 3', synthesized by Metabion international AG), comprising the R285Q substitution and an additional silent mutation for genotyping purposes. After microinjections, zygotes were transferred into pseudopregnant CD-1 foster mice.

To identify p.R285Q Miro1 knock-in founder mice (F0 generation) derived from microinjected zygotes, genomic (g)DNA was isolated from tissue biopsies from mice at the age of 3 to 4 weeks and used to assess Miro1 p.R285Q substitution by PCR (see *Mice genotyping* section 11c below). The knock-in founder mice (F0) were generated at the Institute of Developmental Genetics (Helmholtz Zentrum München, Germany), according to protocols approved by the government of Upper Bavaria and in accordance with the guidelines of the European Community Council Directives.

The animals of p.R285Q Miro1 knock-in mouse line (B6.Miro1^{tmR285QHmgu}) were raised and handled at the University of Luxembourg following the European Union directive 2010/63/EU. Wild-type (wt/wt) were used to generate heterozygous (wt/R285Q) and homozygous (R285Q

/R285Q) p.R285Q Miro1 mutant mice (B6.Miro1^{tmR285QHmgu}), which represent the human p.R272Q Miro1 ortholog mutation, using an heterozygous x heterozygous and homozygous x homozygous breeding strategy.

c. Mice genotyping

Genomic DNA from biopsies was extracted using the Nucleospin DNA RapidLyse kit (Macherey Nagel 740100.250) and quantified using a NanoDrop™ spectrophotometer. DNA was then used to assess p.R285Q Miro1 point mutation integration by PCR. 300 ng of gDNA and 0.2 µM Miro1 forward and reversed primers (Supplementary Table III) were used in a total of 50 µl reaction. The PCR reaction was done in Biometra Thermocycler T Professional Basic 96 (Montreal Biotech 846-070-701) for 5 minutes at 94 °C, followed by 35 cycles of 1 minute at 94 °C denaturation step, 1 minute at 60 °C annealing and 3 minutes 72 °C elongation step, and a final extension step of 7 minutes at 72 °C. PCR products were further digested by TAQI (GoTaq® G2 Flexi DNA Polymerase; Promega M7805) to assess if the point mutation was or not present since its restriction site will be only present in wt Miro1 protein. For that, 12 µl of ddH2O, 10 µl PCR product (no purification is necessary), 0.5 µl NEB Taq1 enzyme, and 2.5 µl 10x smart cut NEB Buffer (Bioke R0149S) were amplified by PCR using the thermocycling program: 40 minutes at 65 °C digestion, 20 minutes 80 °C inactivation step, and a final hold at 7 °C. PCR products and the GeneRuler™ 100 bp Plus DNA ladder were then run in 1.5% agarose gel at 130 V for 45 minutes and revealed in a Biodoc Analyse GBOX machine (Syngene) under UV light using Midori Green advance (Biozyl 617004). Considering the primers used, wt Miro1 will present 2 bands at 334 bp and 569 bp, while homozygous Miro1 p.R285Q mutant will only present one band with 903 bp.

Supplementary Table III PCR primers used for PCR of mice.

Name:	Nucleotide Sequence (5' - 3')
**Miro1R285Q forward**	AAGCCAGGAGACTTGTCCAC
**Miro1R285Q reverse**	TGACCTGCTCCGTACAGTAAG

12. Mouse immunocytochemistry and quantification

a. Immunocytochemistry

Female and male young (3 to 6 month-old) and aged (15 to 18 month-old) mice were used for analyzing the striatal area occupied by the dopamine transporter (DAT) and tyrosine hydroxylase (TH) as well as the substantia nigra pars compacta (SNpc) area occupied by TH and TOM20 within TH, following a previously published protocol.[14]

Serialized parasagittal free-floating, 50 µm-thick sections were obtained using a Leica VT-1000S vibratome and collected in cryoprotective medium composed of a 1:1 ethylene glycol (Sigma Aldrich 61941) and PBS solution, supplemented with 1% w/v polyvinyl pyrrolidone (Sigma Aldrich PVP40).

For staining, sections were washed twice in washing buffer (PBS with 0.1% Triton X-100) with agitation, and permeabilized for 30 minutes in PBS containing 1.5% Triton X-100 and 3% hydrogen peroxide, to deactivate endogenous peroxidases. Sections were further washed twice for five minutes in washing buffer, blocked in PBS with 5% BSA and 0.1% Triton X-100 for 30 minutes, and incubated overnight with primary antibodies (Table 2) in antibody buffer (PBS with 0.3% Triton X-100 and 2% BSA) under strong shaking at room temperature. After three 10 minutes washing steps, sections were incubated with appropriate secondary antibodies (Table 2) in the dark, on shaking conditions for 2 hours at room temperature. For some cases, sections were further incubated with DAPI (Fisher Scientific 622248) 1:1000 for 10 minutes. Finally, sections were washed and mounted on glass slides using Dako fluorescent mounting medium (DAKO S302380).

b. Imaging acquisition and quantification

For quantification of TOM20 within the TH signal, two to four 50 µm brain sections per mouse were used. Images were acquired using a Zeiss Axio Imager Z1 fluorescence microscope with Apotome 2 using the 40x objective with z-projection of 5 µm with stacks of 1 µm. The obtained pictures were converted into optical sectioning using the Zeiss Efficient Navigation (ZEN) Blue Edition software correcting for local bleaching, followed by maximum intensity on the z-projection. Images were then converted to the TIFF format and analyzed using Fiji/ImageJ (RRID SCR_002285).

For DAT and TH, imaging was done using a Zeiss *AxioImager Z1* upright microscope, coupled to a "*Colibri*" LED system to generate fluorescence light of defined wavelengths, an *Mrm3* digital camera for image capture, and equipped with a PRIOR motorized slide stage. The complete Zeiss imaging system was controlled by Zeiss Blue Vision software. To measure TH and DAT, 2-3 striatal sections per mouse containing 3 fields in the dorsal striatum were taken in a 40X objective using the Apotome. Apotome captured images were then modified with the parameters "display optical sectioning" to correct phase errors. Obtained images were converted to TIFF. Then, after thresholding, the percent area occupied by TH and DAT staining were determined using the publicly available imaging software FIJI, using the "restrict to threshold" parameter. Qualitative assessment of phosphorylated S129 α-synuclein was performed on 15 µM deep stacks at a 40 % magnification, followed by maximum intensity projection.

For TH quantification, we first carry out a careful anatomical observation to distinguish SNpc-specific TH-positive neurons from the ones present in other anatomical regions. We recognized the SNpc at four anatomically distinguishable levels using 6-10 sections of 50 µm (spaced 200 µm) per mouse, covering the entire space occupied by the SNpc in each mouse brain. Then, for each section, tiled pictures (2x2) were taken at 10x magnification using the above-mentioned Zeiss imaging system. Next, the region-of-interest (ROI) tool in the FIJI software was used to segment the area occupied only by SNpc TH-positive neurons within each section. After thresholding, the area occupied (in pixels) by TH-positive neurons was measured. The four distinguishable anatomical levels of the SNpc were measured using 2-3 sections/level in each mouse. Values for each level were averaged separately. Thus, 4 final area values, representative of the areas occupied by TH-positive neurons in each anatomical level of the SN, were obtained for each mouse. To obtain one representative value for each brain hemisphere, these 4 values were summed up ("cumulated SN surface"), and converted to mm^2 for graphical representation. Detailed information on the method and its correlation with stereological cell counts can be found in Ashrafi et al.[14]

The mouse genotype was blinded for the experimenter during staining, acquisition and analysis.

13. Mouse behavior assay - Home cage activity

The LabMaster system (TSE Systems) was used to record and analyze the spontaneous home cage activity of the mice. The mouse cage was surrounded by sensor frames with which the number of

beam brakes was quantified and analyzed in 15 minutes intervals. The recorded data provided information on total activity, movement, and rearing. Additionally, the volume of consumed food and water was quantified. The data was recorded over a period of 22 hours, which started with about 30 minutes of light phase before a 12 hour phase of darkness. The measurement happened following the same day and night light cycle that mice were used to. All mice were analyzed in individual cages and afterward reunited with their cage mates. Measurements were done in 20-month-old mice.

14. Mouse striatal dopamine measurement

Striatum, dissected as explained in *Materials and methods* Mice Brain processing section, was used to quantify the abundance of the neurotransmitter dopamine in 15-month-old mice. Tissue was used to extract and measure dopamine by GS-MS based on a previously published method.[15,16] Briefly, 500 µl of methanolic extraction fluid (4:1, methanol/water mixture, v/v) was added to 50 mg mouse brain (striatum). The extraction fluid contained an internal standard mix, consisting of U-13C5 ribitol (c = 2 µg/ml; Omicron Biochemicals ALD-062) and pentanedioic-d6 acid (c = 2 µg/ml; C/D/N Isotopes Inc. D-5227). Samples were subsequently homogenized using a Precellys24 homogenizer (Bertin Technologies) using 600 mg ceramic beads (1.4 mm) and one 30 seconds cycle at 6,000 rpm at 0 to 5 °C. Then, 250 µl of a 0.1 mol/l hydrochloric acid solution, including dopamine-D4 (c = 15 µmol/l) was added to the homogenate. Polar metabolites were extracted by adding 400 µl of chloroform. After incubation under shaking for 15 minutes at 2,000 rpm at 4 °C (Eppendorf ThermoMix Comfort), samples were centrifuged for 5 minutes at 21,000g at 4 °C. 80 µl of the upper phase containing the polar metabolites were transferred into a GC glass vial with micro insert and evaporated at -4 °C for 4 hours, followed by an adaptation phase to room temperature for 25 minutes (Labconco CentriVap). Samples were submitted to subsequent GC-MS analysis.

Supplementary References:

1. Monzel AS, Smits LM, Hemmer K, et al. Derivation of Human Midbrain-Specific Organoids from Neuroepithelial Stem Cells. *Stem Cell Reports*. 2017;8(5):1144-1154. doi:10.1016/j.stemcr.2017.03.010

2. Zagare A, Gobin M, Monzel AS, Schwamborn JC. A robust protocol for the generation of human midbrain organoids. *STAR Protoc*. 2021;2(2):100524. doi:10.1016/j.xpro.2021.100524
3. Hao Y, Hao S, Andersen-Nissen E, et al. Integrated analysis of multimodal single-cell data. *Cell*. 2021;184(13):3573-3587.e29. doi:10.1016/j.cell.2021.04.048
4. Stuart T, Butler A, Hoffman P, et al. Comprehensive Integration of Single-Cell Data. *Cell*. 2019;177(7):1888-1902.e21. doi:10.1016/j.cell.2019.05.031
5. Becht E, McInnes L, Healy J, et al. Dimensionality reduction for visualizing single-cell data using UMAP. *Nat Biotechnol*. 2019;37(1):38-44. doi:10.1038/nbt.4314
6. Ben-Ari Fuchs S, Lieder I, Stelzer G, et al. GeneAnalytics: An Integrative Gene Set Analysis Tool for Next Generation Sequencing, RNAseq and Microarray Data. *Omi A J Integr Biol*. 2016;20(3):139-151. doi:10.1089/omi.2015.0168
7. Andrews S. FASTQC. A quality control tool for high throughput sequence data. Published online 2010. https://www.bibsonomy.org/bibtex/2b6052877491828ab53d3449be9b293b3/ozborn
8. Schubert M, Lindgreen S, Orlando L. AdapterRemoval v2: rapid adapter trimming, identification, and read merging. *BMC Res Notes*. 2016;9(1):88. doi:10.1186/s13104-016-1900-2
9. Dobin A, Davis CA, Schlesinger F, et al. STAR: ultrafast universal RNA-seq aligner. *Bioinformatics*. 2013;29(1):15-21. doi:10.1093/bioinformatics/bts635
10. Liao Y, Smyth GK, Shi W. The R package Rsubread is easier, faster, cheaper and better for alignment and quantification of RNA sequencing reads. *Nucleic Acids Res*. 2019;47(8):e47-e47. doi:10.1093/nar/gkz114
11. Love MI, Huber W, Anders S. Moderated estimation of fold change and dispersion for RNA-seq data with DESeq2. *Genome Biol*. 2014;15(12):550. doi:10.1186/s13059-014-0550-8
12. Zhu A, Ibrahim JG, Love MI. Heavy-tailed prior distributions for sequence count data: removing the noise and preserving large differences. Stegle O, ed. *Bioinformatics*. 2019;35(12):2084-2092. doi:10.1093/bioinformatics/bty895
13. Hiller K, Hangebrauk J, Jäger C, Spura J, Schreiber K, Schomburg D. MetaboliteDetector: comprehensive analysis tool for targeted and nontargeted GC/MS based metabolome

analysis. *Anal Chem*. 2009;81(9):3429-3439. doi:10.1021/ac802689c

14. Ashrafi A, Garcia P, Kollmus H, et al. Absence of regulator of G-protein signaling 4 does not protect against dopamine neuron dysfunction and injury in the mouse 6-hydroxydopamine lesion model of Parkinson's disease. *Neurobiol Aging*. 2017;58:30-33. doi:10.1016/j.neurobiolaging.2017.06.008
15. Jaeger C, Glaab E, Michelucci A, et al. The mouse brain metabolome: region-specific signatures and response to excitotoxic neuronal injury. *Am J Pathol*. 2015;185(6):1699-1712. doi:10.1016/j.ajpath.2015.02.016
16. Jäger C, Hiller K, Buttini M. Metabolic Profiling and Quantification of Neurotransmitters in Mouse Brain by Gas Chromatography-Mass Spectrometry. *Curr Protoc Mouse Biol*. 2016;6(3):333-342. doi:10.1002/cpmo.15
