# Supplementary material for: Parkinson’s disease mutant Miro1 causes mitochondrial dysfunction and dopaminergic neuron loss
Source: Brain. 2025 Feb 6;148(10):3607–22. doi: 10.1093/brain/awaf051 (PMC12493065; doi:10.1093/brain/awaf051)
Supplement: awaf051_Supplementary_Data [file awaf051_supplementary_data.zip › brain-2024-01618-File012.pdf]

Supplementary Western blotting data

Parkinson's disease mutant Miro1 causes mitochondrial dysfunction and dopaminergic neuron loss

Axel Chemla[1], Giuseppe Arena[1], Ginevra Sacripanti[1], Kyriaki Barmpa[1], Alise Zagare[1], Pierre Garcia[1,2], Vyron Gorgogietas[1], Paul Antony[1], Jochen Ohnmacht[3], Alexandre Baron[1], Jaqueline Jung[4], Frida Lind-Holm Mogensen[3,5], Alessandro Michelucci[3], Anne-Marie Marzesco[1,§], Manuel Buttini[1,2], Thorsten Schmidt[4], Anne Grünewald[1,6], Jens C. Schwamborn[1,*], Rejko Krüger[1,3,7,*], Cláudia Saraiva[1,*]

Affiliations:

[1]Luxembourg Centre for Systems Biomedicine (LCSB), University of Luxembourg, L-4362 Esch-sur-Alzette, Luxembourg

[2]Luxembourg Center of Neuropathology (LCNP), Laboratoire National de Santé, L-3555, Dudelange, Luxembourg

[3] Luxembourg Institute of Health (LIH), L-1445 Luxembourg, Luxembourg

[4]Institute of Medical Genetics and Applied Genomics, University of Tübingen, 72076 Tübingen, Germany

[5]Faculty of Science, Technology and Medicine, University of Luxembourg, L-4365 Esch-sur-Alzette, Luxembourg

[6]Institute of Neurogenetics, University of Lübeck, 160 Lübeck, Germany

[7]Centre Hospitalier de Luxembourg, L-1210, Luxembourg, Luxembourg

[§] Current address: University Medical Center Hamburg-Eppendorf, Center for Molecular Neurobiology Hamburg, 20246 Hamburg, Germany

[*] Senior authors

Correspondence to: jens.schwamborn@uni.lu (JCS), rejko.krueger@lih.lu (RK) and claudia.saraiva@uni.lu (CS)

Supplementary Western blotting data

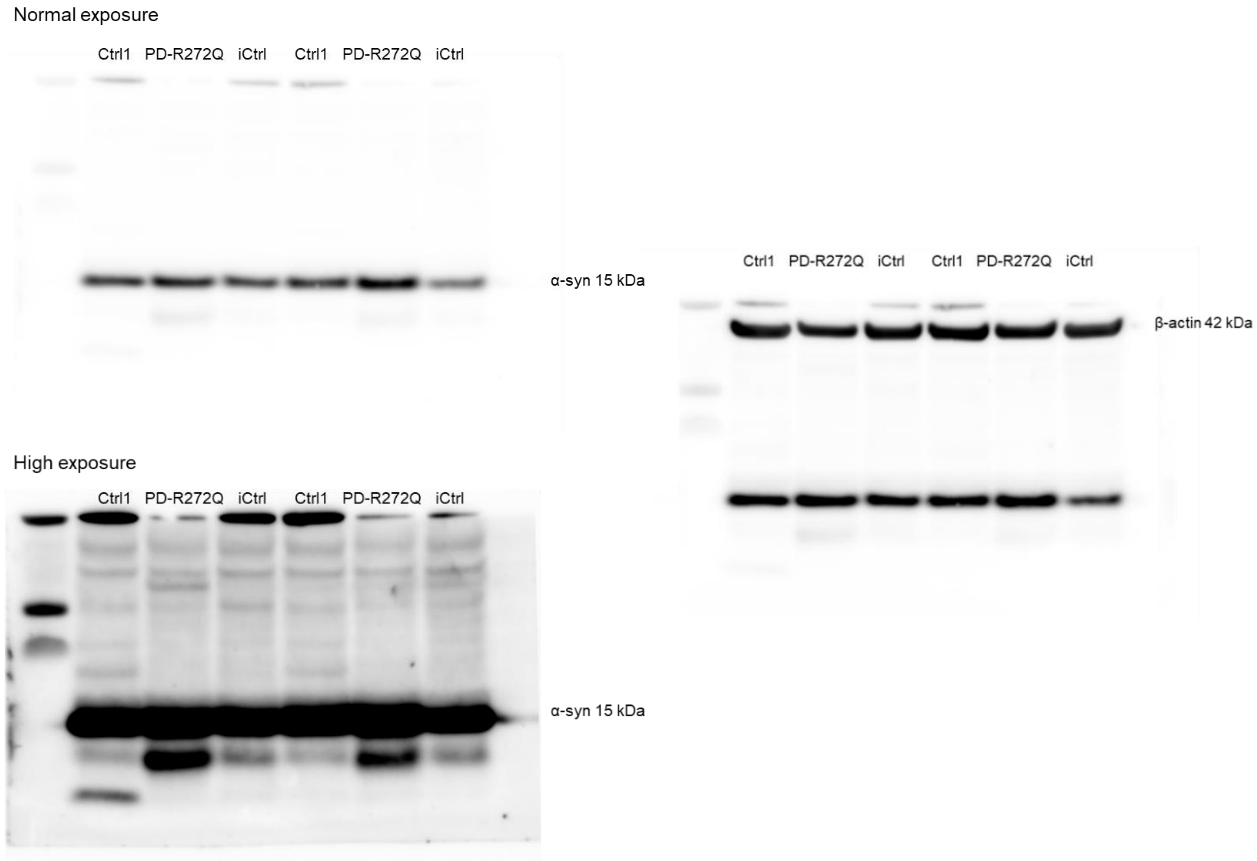

WB1. Original full length membrane referent to the data displayed at Main Figure 4B. Left: α-synuclein normal (top) and high (bottom) exposures. Right: full membrane showing β-actin.

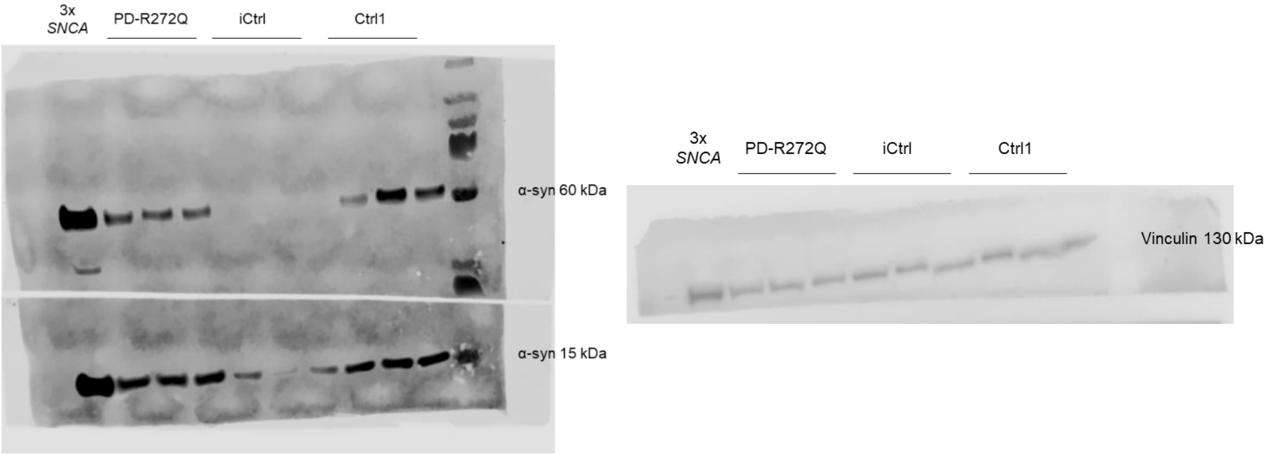

WB2. Original membrane shown at Main Figure 4F, blotted for α-synuclein (left) and Vinculin (right).

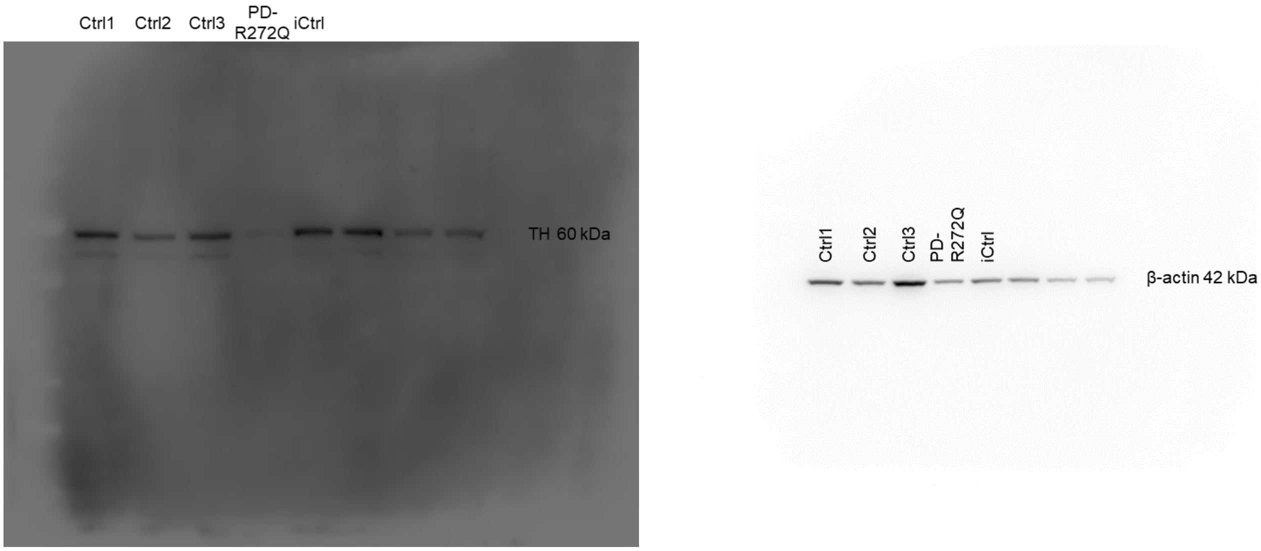

WB3. Original membrane shown at Main Figure 5A, blotted for tyrosine hydroxylase (TH, left) and β-actin (right).

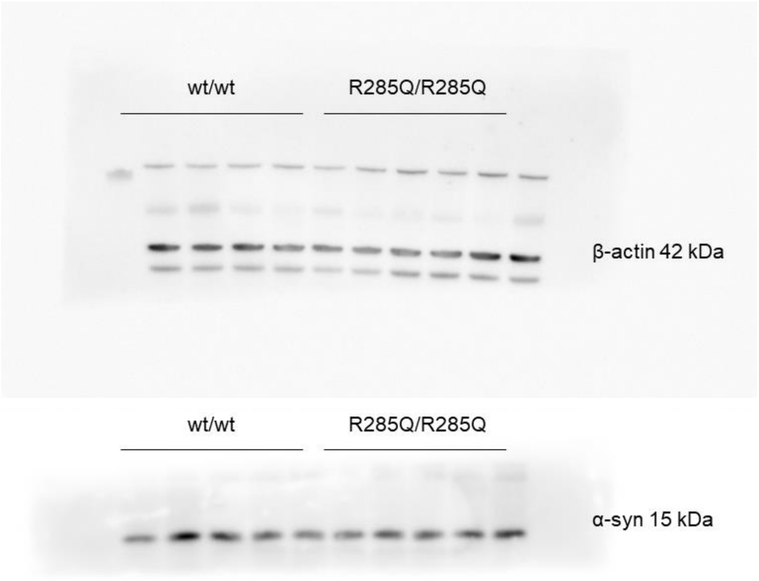

WB4. Original membrane shown at Main Figure 6E, blotted for β-actin (top) and α-synuclein (α-syn, bottom).

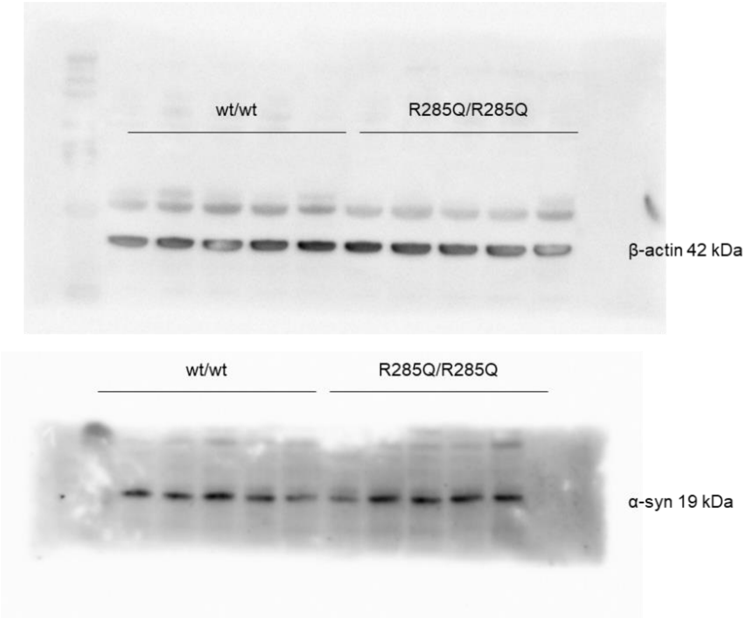

WB5. Original membrane shown at Main Figure 6F, blotted for β-actin (top) and phosphorylated S129 α-synuclein (α-syn, bottom).

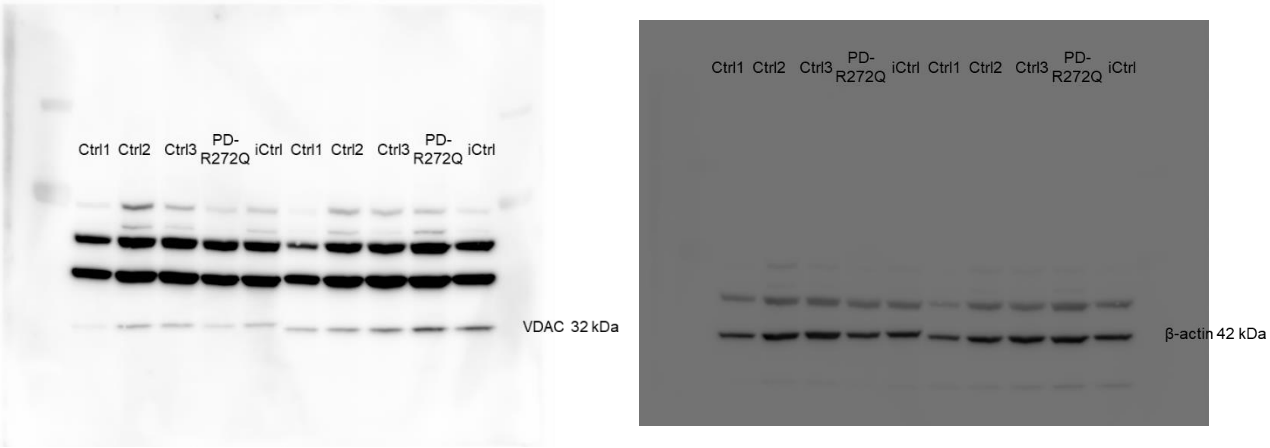

WB6. Original membrane shown at Supplementary Figure 4B, blotted for VDAC (left) and β-actin (right).

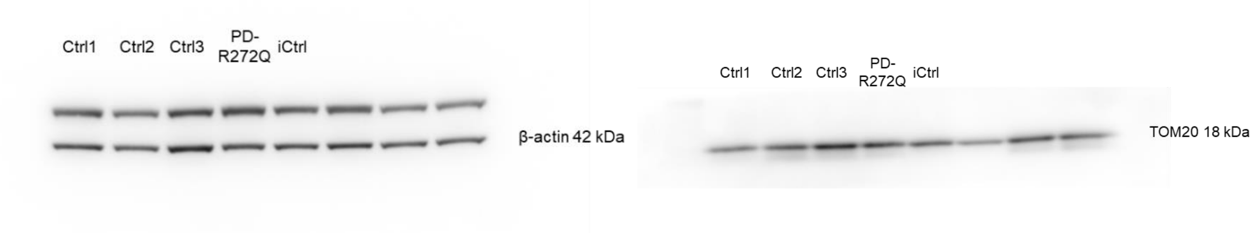

WB7. Original membrane shown at Supplementary Figure 4C, blotted for TOM20 (right) and β-actin (left).

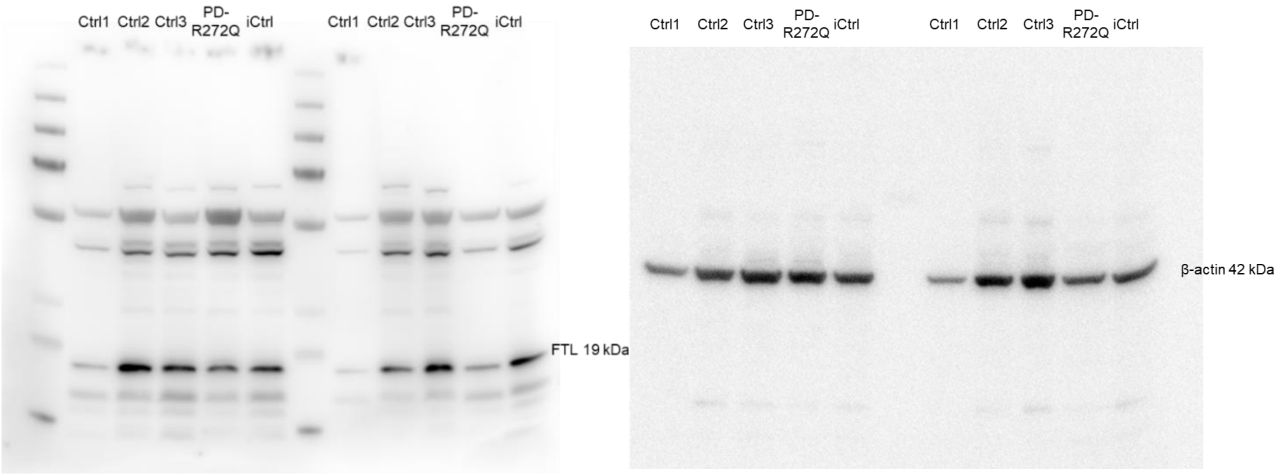

WB8. Original membrane shown at Supplementary Figure 4D, blotted for FTL (left) and β-actin (right).

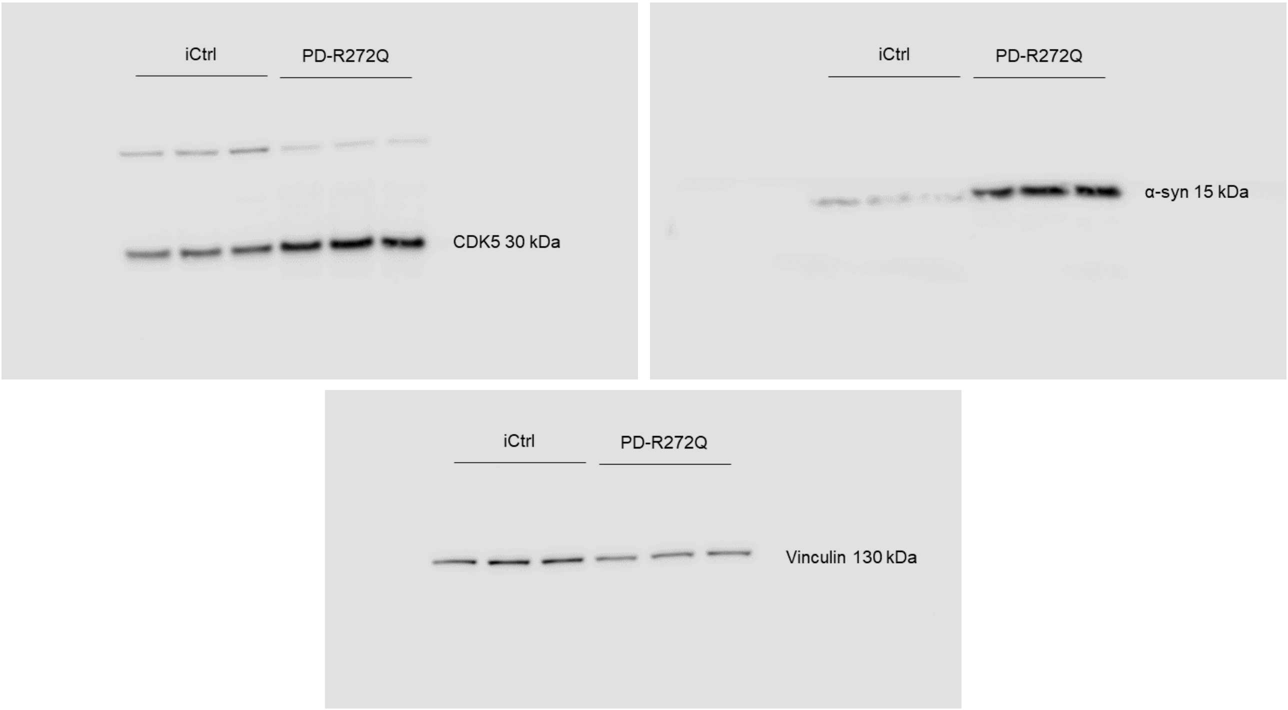

WB9. Original membrane shown at Supplementary Figure 6E, blotted for CDK5 (top left), α-synuclein (top right) and vinculin (bottom).

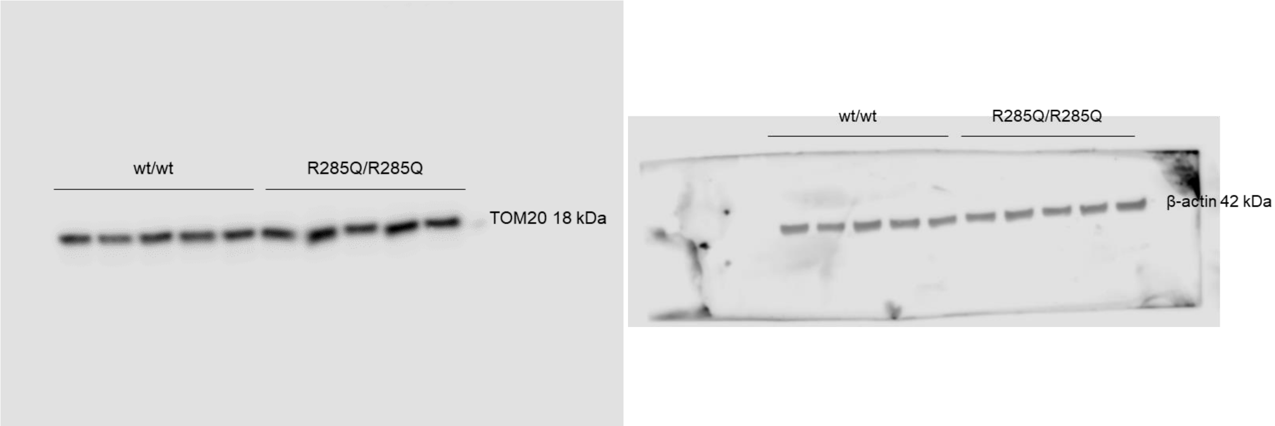

WB10. Original membrane shown at Supplementary Figure 9C, blotted for TOM20 (left) and β-actin (right).

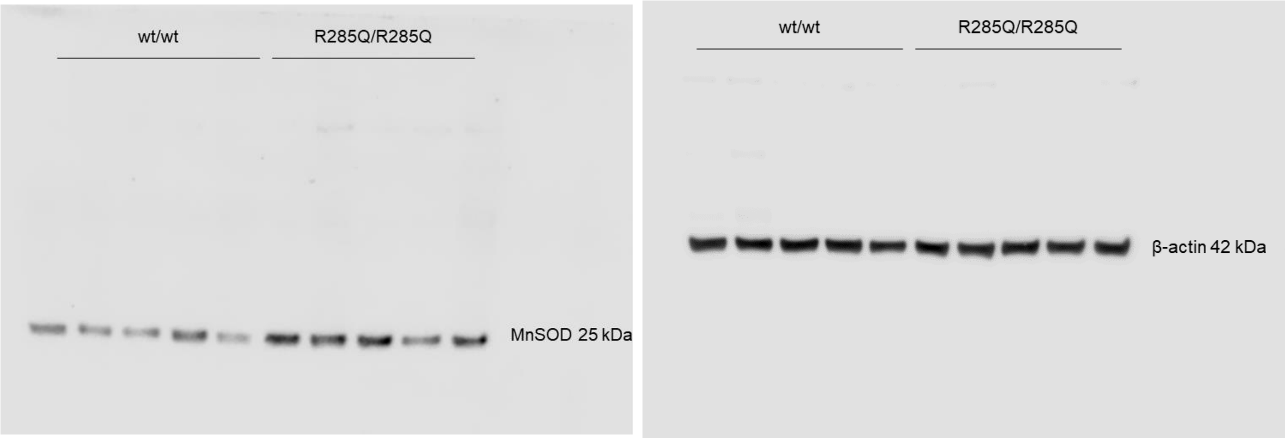

WB11. Original membrane shown at Supplementary Figure 9D, blotted for MnSOD (left) and β-actin (right).

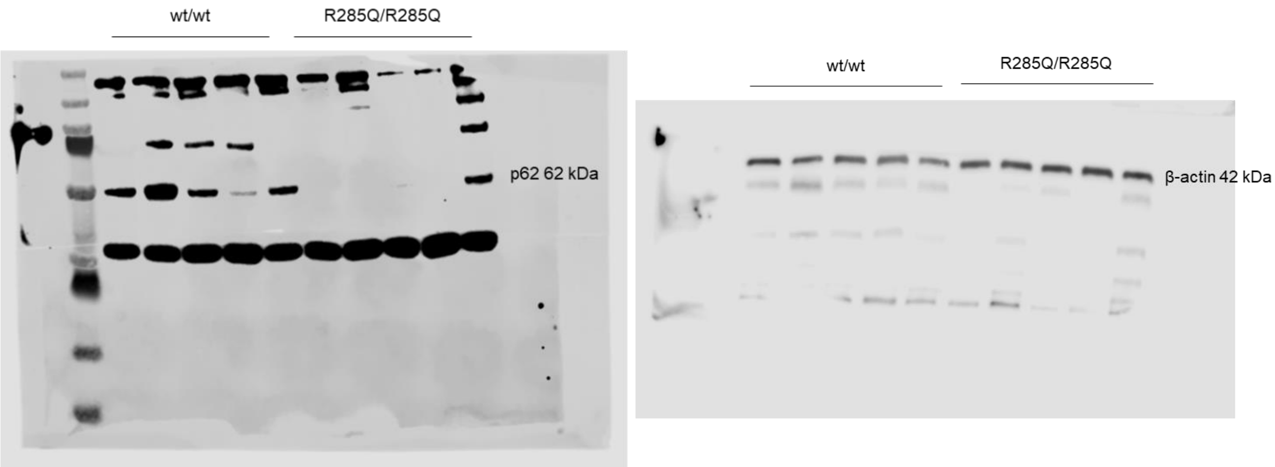

WB12. Original membrane shown at Supplementary Figure 9E, blotted for p62 (left) and β-actin (right).

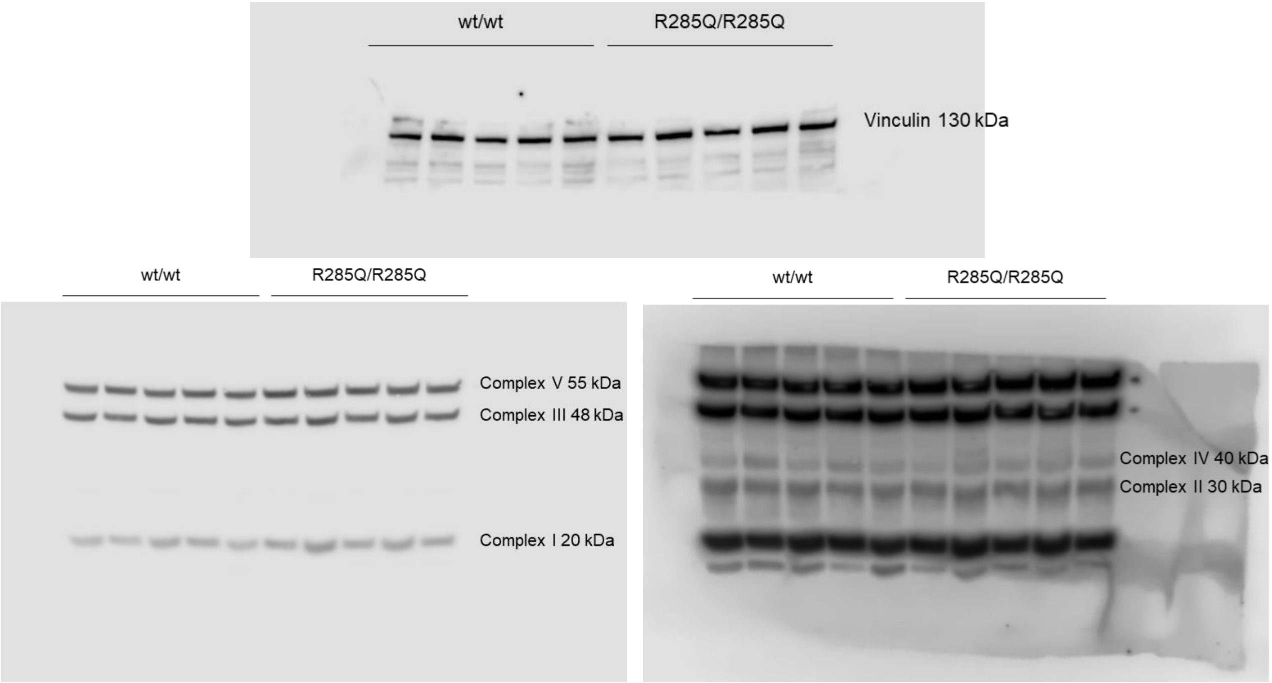

WB13. Original membrane shown at Supplementary Figure 9F, blotted for Vinculin (top) and mitochondrial complex V, III, and I (bottom left) as well as mitochondrial complex IV and II (bottom right).
